# ESIPT fluorescence turn-on sensors for detection of short chain inorganic polyphosphate in water†

**DOI:** 10.1039/d4ob01926a

**Published:** 2024-12-18

**Authors:** Subhra Kanti Roy, Sandra Moser, Tobias Dürr-Mayer, Rahel Hinkelmann, Henning J. Jessen

**Affiliations:** a Institute of Organic Chemistry, Albert-Ludwigs-Universität Freiburg Albertstraße 21 79104 Freiburg im Breisgau Germany henning.jessen@oc.uni-freiburg.de; b CIBSS—Centre for Integrative Biological Signalling Studies, University of Freiburg 79104 Freiburg Germany

## Abstract

We introduce two water-soluble excited state intramolecular proton transfer (ESIPT) based fluorescent turn-on probes responding to inorganic polyphosphates. These ESIPT probes enable specific detection of short-chain inorganic polyphosphates over a range of different condensed phosphates. The probes are weakly emissive in their off-state due to the blocking of ESIPT by Cu^2+^ coordination. Removal of the copper ion through decomplexation by the analyte accesses the on-state. The probes detect polyphosphates over other biologically occurring phosphates, pyrophosphate, and nucleotides such as ATP, ADP, GTP. An optimal fluorescence response is observed with the short-chain polyphosphate polyP_8_. Furthermore, the probe shows selectivity towards linear polyphosphates over cyclic metaphosphates. The rapid ‘turn-off–turn-on’ fluorescence responses upon consecutive addition of Cu^2+^ and polyP_8_ are reversible, further highlighting sensor performance in an aqueous environment. One of the sensors is then used to monitor polyP digestion by an exopolyphosphatase (PPX).

## Abstract 2

We introduce two water-soluble excited state intramolecular proton transfer (ESIPT) based fluorescent turn-on probes responding to inorganic polyphosphates.

## Graphical abstract

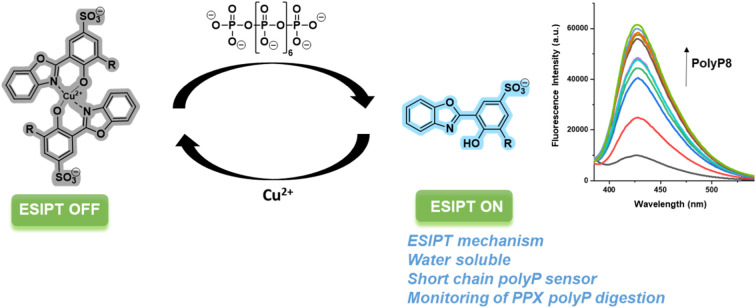

## Introduction

Inorganic polyphosphate (polyP), an ancient macromolecule also considered to be a ‘molecular fossil’, has been detected in all living organisms.^1^ The linear anionic polymer consists of three to several thousands of units of orthophosphate (P_*i*_) monomers, interconnected by high-energy phosphoanhydride bonds.^2^ PolyP plays diverse roles in both prokaryotic and eukaryotic cells.^3^ For example, it is involved in energy storage,^4^ molecular chaperoning, regulating apoptosis,^5^ hemostatis and thrombosis,^6^ bone mineralization and proinflammatory functions,^7^ and oxidative stress response.^8^ Moreover, polyP has been linked to cognitive performance as well as neurodegenerative conditions such as Parkinson's disease, Alzheimer's disease, and amyotrophic lateral sclerosis.^9^ Short-chain polyPs are found in parasites such as *Trypanosoma cruzi*, *Trypanosoma brucei*, and *Leishmania major* where they are important for parasite metabolism.^10^ Besides its biological roles, polyP is also important in biotechnological applications such as ATP regeneration systems using polyP kinases.^11^ Furthermore, the ability of microorganisms to accumulate polyP is used to remove phosphates from wastewater.^12^

Given the diverse areas in which polyP plays an important role, there is a significant demand for simple chemical sensors that enable the detection of polyP, ideally with selectivity regarding chain length. For biological applications, such detection would benefit from selectivity over other abundant condensed phosphates, such as *e.g.* ATP. Currently, there is only a very limited number of colorimetric and fluorescent probes available to visualise polyPs. The use of 4′,6-diamidino-2-phenylindole (DAPI), a yellow fluorescent compound in complex with polyP, is the most popular approach, owing to its significant fluorescence shift when binding to polyP.^13^ In addition to DAPI, toluidine blue has also been used for the detection of polyPs. However, literature reports that only polyPs with a chain length longer than 15 units are detected by DAPI and toluidine blue.^1*d*,14^ Also, some interferences with other phosphate containing molecules have been reported. For example, characteristic yellow fluorescence is also emitted from DAPI-inositol phosphate complexes.^15^ As an alternative to DAPI-polyP emission, Pavlov's group reported fluorescent benzimidazolinium dyes, but these also require longer chain polyPs.^16^ Thus, currently the detection of polyPs with fluorescent sensors is not covering polyPs with comparatively short-chains in the single digit range.

**Scheme 1:**
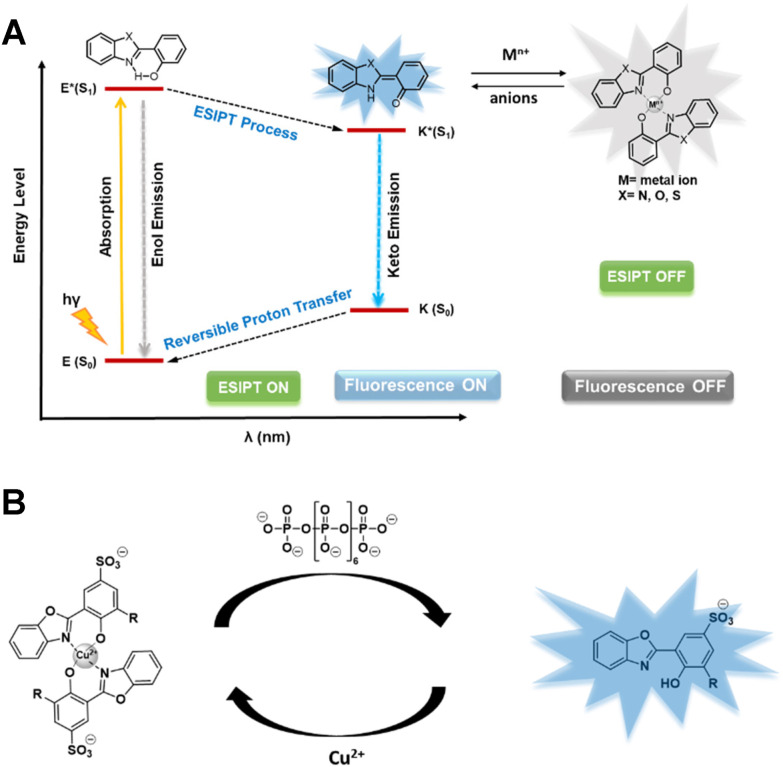
(a) Schematic diagram of photophysical processes involved in ESIPT based sensors, (b) 2-hydroxy phenyl benzoxazole-Cu^2+^ complex (square planar geometry is assumed) as a decomplexation based sensor for polyphosphate with an ESIPT fluorophore.

Selective fluorescent sensors of important (condensed) phosphates, such as P_*i*_ and pyrophosphate (PP_*i*_), have been developed using different strategies, including hydrogen bonding interaction, coordination chemistry, displacement assay/decomplexation, and aggregation-induced emission/quenching.^17^ Over the past few decades, the design of fluorogenic sensors based on Excited-State Intramolecular Proton Transfer (ESIPT) has gained considerable attention due to their useful emissive properties including very large Stokes shifts (∼200 nm). Moreover, such sensors often combine ultra-fast rates with little reabsorption.^18*a*^ In the ESIPT process, fluorophore tautomers in the ground state and excited states of enol- (E and E*) and keto-forms (K and K*) generate four photochemical states (Scheme 1a). Because of its unique ^‘^turn-off–turn-on’ photoisomerization feature and low background interference, an ESIPT-active molecule provides sensing opportunities in diverse applications. ESIPT based sensors have been used for the detection of a variety of anions, most notably in the context of this study: PP_*i*_.^18^

The choice of using an ESIPT-based strategy is promising due to its current success in detection of numerous environmentally and biologically significant analytes.^18*a*,19^ However, achieving selective detection of polyP over PP_*i*_ and other anions like nucleotides is not a trivial task. Moreover, the sensor should rapidly respond in an aqueous environment. This adds a solubility problem, as a majority of the known ESIPT based fluorescence sensors are only sparingly soluble in aqueous solution, which limits their widespread applicability.^20^ Herein, we report the first chemoselective, reversible, water-soluble, short chain inorganic polyP sensors that work in an aqueous environment and rely on the ESIPT mechanism providing large Stokes shifts for diverse applications.

## Results and discussion

### Ligand design and synthesis

**Scheme 2:**
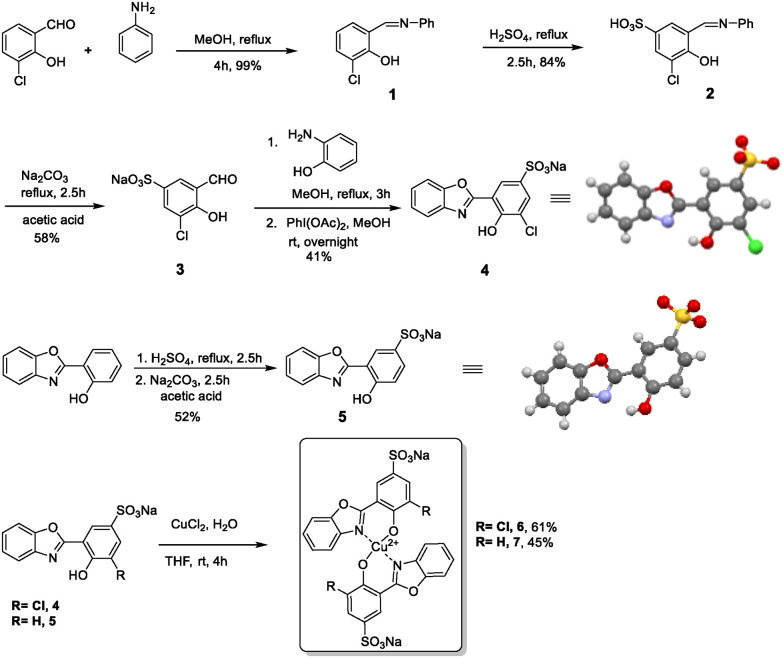
Synthesis of 2-hydroxy-phenyl-benzoxazole derivatives and their Cu-complexes. The counter ions of the crystal structures of 4 and 5 were omitted for clarity.

The ESIPT phenomenon is linked to the presence of an intramolecular hydrogen bond, usually between a proton donor (–OH/–NH) and a proton acceptor (–C=O/heterocyclic N). The fluorophore 2-hydroxy phenyl benzoxazole and its derivatives could provide on–off switching properties of the ESIPT signal through metal complexation, leading to metal induced fluorescence quenching. Decomplexation of the metal through the analyte would then lead to recovery of the ESIPT signal. Provided the decomplexation can be selective, as previously shown in disassembly strategies, *e.g.* by the Zelder group for PP_*i*_,^21^ one could design a polyphosphate responsive ESIPT sensor. To confer solubility in aqueous medium, a charged group, such as a sulfonate, would be beneficial. Based on the above considerations, fluorophore 4 was synthesized in four steps starting from 3-chloro-2-hydroxybenzaldehyde (Scheme 2). Condensation of aniline with 3-chloro-2-hydroxybenzaldehyde afforded imine 1.^22^ The imine intermediate 1 was refluxed with conc. H_2_SO_4_ to introduce a sulfonate group at the 5 position in 84% yield. Treatment with Na_2_CO_3_ gave the sodium salt 3 in 58% yield. Afterwards, condensation of 3 with 2-aminophenol followed by *in situ* cyclization afforded the ESIPT fluorophore 4 in 41% yield (Scheme 2). Another ESIPT fluorophore 5 was synthesized in two steps in 52% yield (Scheme 2). These two fluorophores with slightly modulated photophysical properties served as basis for our follow-up studies.

The main challenge for designing fluorescence sensors for negatively charged species is that its hydration tendency reduces the sensors’ ability to interact with the target analyte.^23^ One potential solution to surmount this challenge involves the employment of a displacement strategy where initially the fluorescent probe is complexed to a metal ion that quenches the fluorescence (metal-ion induced fluorescence quenching).^24^ Then, decomplexation of the metal ion by the negatively charged analyte leads to a restored ESIPT fluorophore. Copper complexes have been used for P_*i*_ and PP_*i*_ sensing,^25^ however no other condensed phosphate chains have been examined with related complexes.

To generate a metal quenched ESIPT-OFF probe 6, we reacted probe 4 with 0.5 equiv. of CuCl_2_ at room temperature (61% yield). The formation of this complex in a 2 : 1 stoichiometry was confirmed by HRMS data (Fig. S7†). Furthermore, we confirmed the correct stoichiometry by changes in photophysical properties and Job's plot analysis (see next section). While the Cl substituent in 6 was mainly installed to enable further potential modifications, we also generated a non-chlorinated version to study and compare the impact of substitution on the photophysical properties and selectivity regarding different anions. An identical complexation of 5 with Cu^2+^ in a 2 : 1 stoichiometry was conducted, resulting in complex 7.

### Photophysical studies of the fluorophores in the presence of Cu^2+^

**Fig. 1:**
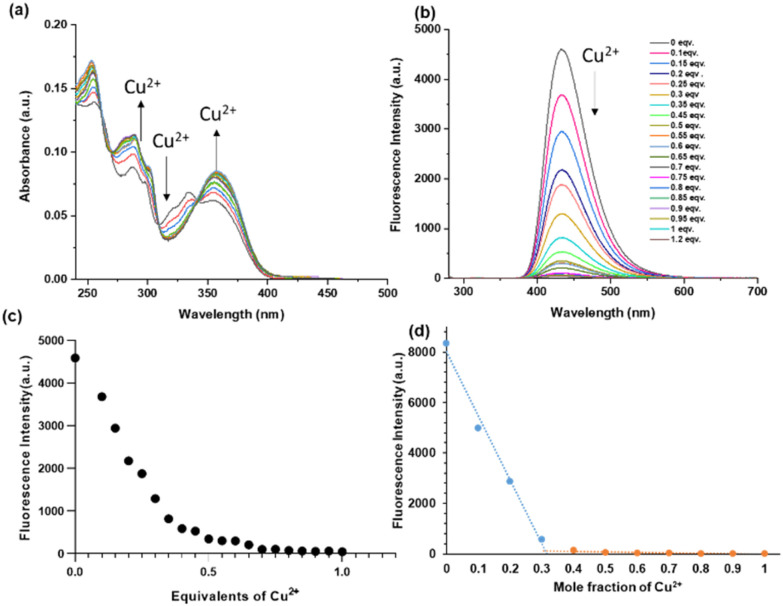
(a) Absorption titration spectra of 4 (10 μM) in a HEPES-buffer (10 mM, pH = 7.4) in the presence of increasing concentrations of Cu^2+^ (0–1 equiv.). (b) Fluorescence titration spectra of 4 (5 μM) in a HEPES-buffer (10 mM, pH = 7.4) in the presence of increasing concentrations of Cu^2+^ (0–1.2 equiv.). (c) Emission intensity at 434 nm in the presence of increasing concentrations of Cu^2+^ (0–1 equiv.), (d) Job's plot of compound 4 with Cu^2+^ according to the method of continuous variation. [4] + [Cu^2+^] = 10 μM. Excitation and emission were recorded at 254 nm and 434 nm, respectively.

Spectrophotometric titration was used to examine the UV-Vis absorption spectrum of ligand 4 in 10 mM HEPES buffer solution at pH 7.4 (Fig. 1a). A prominent band with an absorption peak at 368 nm was visible. With increasing addition of Cu^2+^ (from 0.1–0.5 equiv.) the absorption maximum at 368 nm increased, and adding more equivalents did not elicit additional changes indicating a 2 : 1 complexation with Cu^2+^.

The fluorescence spectra of ligand 4 in an aqueous solution (10 mM HEPES buffer, pH = 7.4) are shown in Fig. 1b (see ESI Fig. S2 and S3† for 5) along with their changing intensities upon addition of Cu^2+^ ion in the solution. While excited at 254 nm, 4 exhibited a strong emission band centred at 434 nm in the buffer solution with a large Stokes shift of 180 nm, typical for an ESIPT fluorophore. Upon addition of different equivalents of Cu^2+^ (0 to 1 equivalent) into the solution of 4, the fluorescence intensity gradually decreased and a quench in fluorescence was observed at *ca.* 0.5 equivalents (Fig. 1c). The fluorescence intensity decreased linearly until 0.5 equivalents Cu^2+^, at which point it reached a plateau. Increasing from 0.5 to 1 equivalent of Cu^2+^ did not lead to further changes, suggesting that 4 is complexed with Cu^2+^ in a 2 : 1 stoichiometric ratio. Further, the 2 : 1 stoichiometry was confirmed by a Job's plot (Fig. 1d) and the HRMS data for 6 and 7 (Fig. S7 and S8, respectively†).

**Fig. 2:**
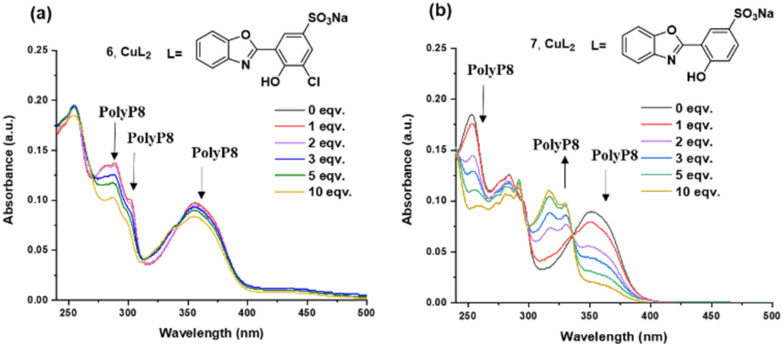
Absorption titration spectra of (a) 6 (2.5 μM) in a HEPES-buffer (10 mM, pH = 7.4), (b) 7 (5 μM) in HEPES-buffer (10 mM, pH = 7.4), subjected to increasing concentration of polyP_8_. Spectra were recorded after 2 minutes of incubation.

Next, we investigated the decomplexation of Cu^2+^ from the probes 6 and 7 with different condensed phosphates, which would release the ESIPT fluorophore. Long polyphosphates are obtained as polydisperse mixtures of varying chain length, rendering analytics more difficult. Initially, we therefore looked into the decomplexation of the sensor with polyP_8_, which is currently the longest monodisperse polyP available by synthesis. Also shorter polyphosphates are accessible in monodisperse form through chemical synthesis (see ESI section 12†).^26^ Addition of polyP_8_ to complex 6 (in 10 mM HEPES buffer at pH = 7.4) led to a gradual decrease of the absorption peaks at 288 nm, 300 nm and 356 nm (Fig. 2a). In case of complex 7, the absorption peaks at 254 nm, 284 nm and 351 nm also decreased while absorption increased at 317 nm with increasing polyP_8_ concentration (Fig. 2b). Interestingly, the decrease in absorption intensities was much higher with non-chlorinated 7 compared to chlorinated 6. The structures of complexes 6 and 7 are tentatively assigned to be square planar, but we could not obtain crystal structures of the compounds. The very different behaviour might be a result of distortions of the geometry based on steric constraints potentially exerted by the chlorine atom.

**Fig. 3:**
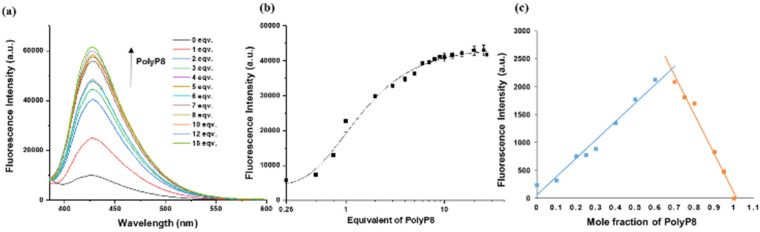
(a) Fluorescence emission spectra (excitation, 254 nm) of 6 (2.5 μM) with 0–15 equivalents of polyP_8_ in 10 mM HEPES (pH 7.4). (b) Plot of the change of emission intensity at 434 nm against polyP_8_ concentration. Number of replicates *n* = 3. (c) Job's plot of compound 6 with polyP_8_ according to the method of continuous variation. [6] + [polyP8] = 5 μM. Excitation = 254 nm, emission = 434 nm.

Both complexes were further studied regarding their ESIPT-ON state upon titration. PolyP_8_ sensing of weakly emissive 6 was investigated by using fluorescence spectroscopy. Upon addition of polyP_8_ sodium salt into a solution of 6 (2.5 μM in 10 mM HEPES buffer at pH = 7.4), the fluorescence emission intensity (*λ*_ex_ = 254 nm) centred at *λ*_em_ = 434 nm was progressively enhanced within 2 minutes of polyP_8_ addition. An eightfold enhancement in the fluorescence intensity was observed when 10 equiv. of polyP_8_ were added (Fig. 3a). We attribute these significant changes in the spectral properties to the removal of Cu^2+^ from the ligand's coordination sphere by polyP_8_, releasing the ESIPT fluorophore characterized by its large Stokes shift.

During excitation at 254 nm, we observed a polyP_8_ concentration-dependent fluorescence increase at 434 nm (Fig. 3b). The titration resulted in a sigmoidal curve, by using nonlinear regression analysis of the polyP_8_ induced emission data. Notably, the fluorescence intensity did not decrease with time (Fig. S5†), demonstrating stability of the released fluorophore. To determine the complexation stoichiometry between 6 and polyP_8_, Job's plot analysis was conducted on the basis of the emission intensity at 434 nm (Fig. 3c). The result suggested that a 1 : 2 stoichiometry was found between 6 and polyP_8_, while [6] + [polyP8] = 5 μM was maintained throughout the experiment.^24^ Probe 7 (5 μM concentration) behaved in a similar way and showed ESIPT-ON under titration with polyP_8_ (excitation at 250 nm, emission at 430 nm; see Fig. S4†).

### Selectivity studies of the probes over other phosphates

**Fig. 4:**
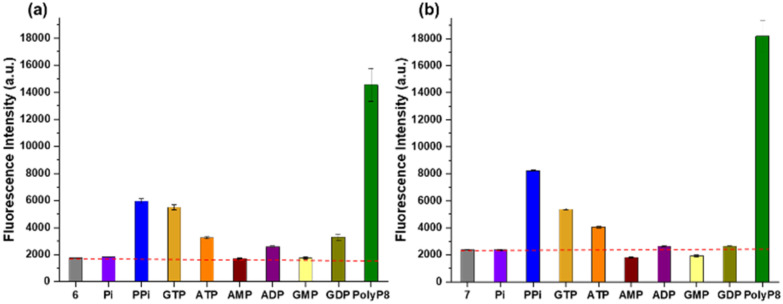
(a) Emission intensity of 6 (2.5 μM) at 434 nm and (b) emission intensity of 7 (5 μM) at 430 nm 2 minutes after addition of 10 equivalents of the different anions in 10 mM HEPES buffer solution at pH = 7.4. Excitation at 254 nm and 250 nm, respectively. Number of replicates *n* = 3. Red dashed line: background emission intensity of the sensors.

Given the excellent ESIPT-ON properties of the sensors in response to polyP_8_, we were now interested in profiling the selectivity of 6 and 7 with respect to other condensed phosphates. Both probes 6 and 7 were incubated with a wide range of biologically relevant (condensed) phosphates, like monophosphate (P_*i*_), pyrophosphate (PP_*i*_), several abundant nucleotides such as ATP, AMP, ADP, GTP, GMP and GDP under the same conditions of 10 mM HEPES at pH = 7.4 with 2 minutes of incubation time. The emission intensities elicited by all these analytes were measured. Fig. 4a shows that the decomplexation and resulting ESIPT fluorescence of 6 reached a maximum in presence of polyP_8_ as compared to other tested anions. Both sensors 6 and 7 had a similar selectivity profile regarding anions. However, the emission intensity was higher for the released ESIPT fluorophore 4, enabling its application at twofold lower concentrations. Quantum yields for 4 and 5 were determined to be 0.33 and 0.16 respectively (Experimental details in the ESI†), directly explaining the higher sensitivity of 6 in the assays. Such values are comparably high for ESIPT fluorophores and must be an effect of the sulfonate group we introduced. The limit of detection (LOD) of polyP_8_ with 6 and 7 were found to be 97 nM and 110 nM, respectively (details in ESI Section 9†).^27^

Both sensors did not respond to phosphate (P_*i*_) and monophosphate esters, such as GMP or AMP. In contrast, both sensors did respond to pyrophosphate (PP_*i*_), but the increment in fluorescence gain is small in both cases. Diphosphate esters like *e.g.* ADP and GDP also elicited a response, but reduced when compared to free PP_*i*_. Nucleoside triphosphates like ATP or GTP also released some of the fluorophore, but by far the largest increment of *ca.* 8-fold was achieved with polyP_8_ in both cases. Consequently, sensors 6 and 7 represent the first selective short-chain polyP sensors that work in an aqueous environment based on an ESIPT-ON mechanism (Fig. 4a and b).

**Fig. 5:**
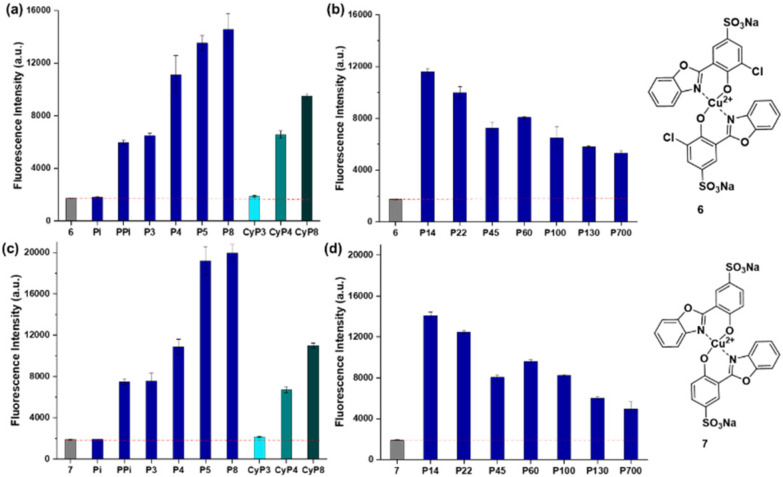
Emission intensity at 434 nm of 6 (2.5 μM) with (a) 25 μM (10 equivalents) of each polyP of different chain length, (b) 125 μM (50 equivalents based on P_*i*_ units) of each polyP of different chain length in 10 mM HEPES buffer solution at pH = 7.4. Excitation at 254 nm. Emission intensity at 430 nm of 7 (5 μM) with (c) 50 μM (10 equivalents) of each polyP of different chain length, (d) 250 μM (50 equivalents based on P_*i*_ units) of each polyP of different chain length in 10 mM HEPES buffer solution at pH = 7.4. Excitation at 250 nm. Number of replicates *n* = 3. Red dashed line: background emission intensity of the sensors.

Next, we investigated the dependence of the signal of sensors 6 and 7 on polyP chain lengths using several synthetic standards and commercial polydisperse references. Interestingly, we observed that with increasing chain length the signal intensity increased, reaching a plateau around polyP_8_,^28^ when incubated under the same conditions (10 mM HEPES, pH = 7.4). Fig. 5 depicts this trend in fluorescence intensity gain from monophosphate to pyrophosphate to polyP_8_ (10 equivalents of each different polyP with 6 or 7). We also examined the fluorescence response of probes 6 and 7 towards different cyclic metaphosphates like cyP_3_, cyP_4_ and cyP_8_.^29^ cyP_4_ and cyP_8_ showed a considerable fluorescence response, unlike cyP_3_. Yet, linear polyP_8_ still elicited a 1.5 times higher response as compared to its cyclic analogue cyP_8_ (Fig. 5a and c).

We also profiled longer polyphosphate chains, which are commercially available. These are polydisperse mixtures of different chain length and so it is difficult to obtain their real concentration. Usually, the phosphate content in thus indicated in terms of P_*i*_ monomers after digestion with exopolyphosphatase (PPX).^1*d*^ After increasing the concentration of the longer chain polyPs (polyP_14_–polyP_700_) to 125 μM for 6 and 250 μM for 7 (in terms of P_*i*_ residues), we observed a trend of reduced response from both sensors 6 and 7 with increasing chain length from polyP_14_ (7-fold increase) to polyP_130_ and polyP_700_ (4-fold increase), indicating that our sensors are most responsive to short-chain polyP with an average chain length of around 8 (Fig. 5b and d).

### Probe 6 is an efficient reversible fluorescent sensor

**Fig. 6:**
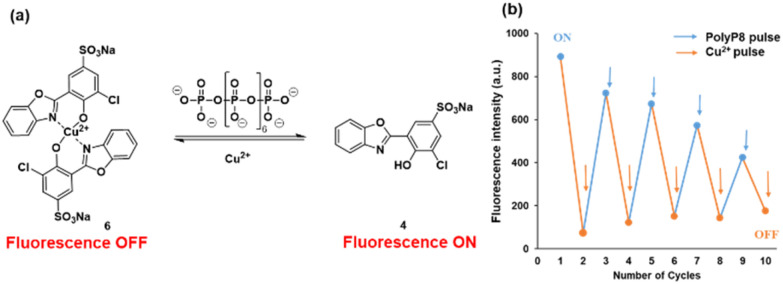
(a) Reversibility of probe 6 with pulses of Cu^2+^ and polyP_8_; (b) emission intensity at *λ*_em_ = 434 nm with alternate addition of Cu^2+^ and polyP_8_ into a solution of 4 (5 μM). Excitation at *λ*_ex_ = 254 nm. Emission was recorded after 2 minutes of addition of the respective compound.

In order to assess the reversibility of probe 6, we investigated its fluorescence emission in response to alternating pulses of polyP_8_ and CuCl_2_. Addition of Cu^2+^ into a solution of 4 leads to a quench of fluorescence at 434 nm due to the formation of 6. The fluorescence was recovered by the addition of polyP_8_ into the solution of 6, leading to the release of 4. Five alternating additions of Cu^2+^ and polyP_8_ showed interconversion of 4 to 6 and back, which indicates that 4 can be used as a reversible fluorescent ‘turn-off–turn-on’ probe for Cu^2+^ and polyP_8_ (Fig. 6b). The concentration of Cu^2+^ and polyP_8_ had to be adjusted in each cycle to obtain reversibility (see details in ESI Section 4†).

### Detection of polyP digestion by probe 6

**Fig. 7:**
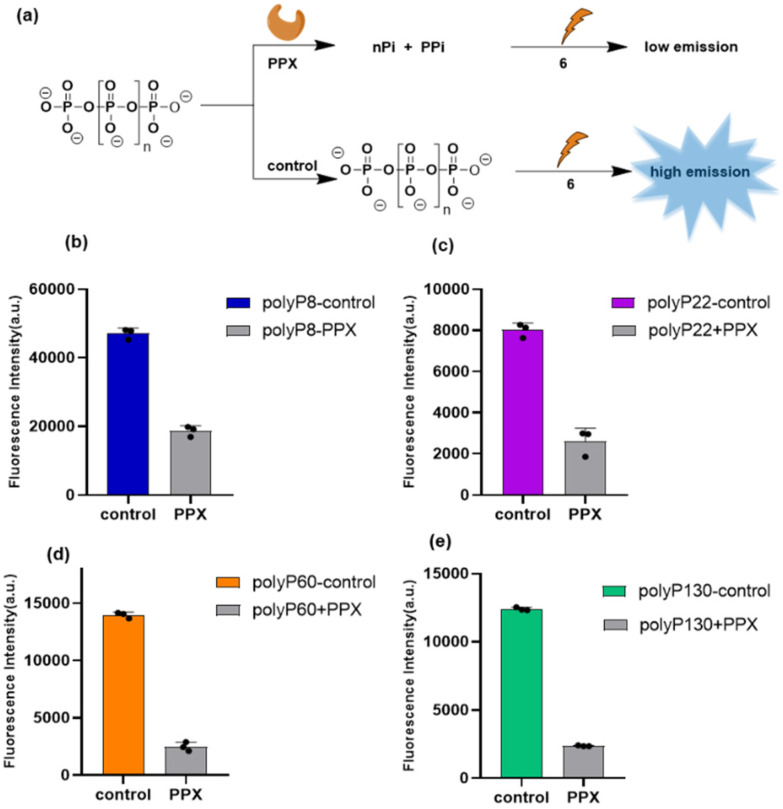
(a) Schematic presentation of monitoring polyphosphate digestion by PPX with sensor 2.5 μM of 6 (10 mM HEPES buffer at pH = 7.4). Emission intensity of 6 (2.5 μM) at 434 nm of polyP digestion by PPX of polyP_8_ (b), polyP_22_ (c), polyP_60_ (d) and polyP_130_ (e). Excitation at 254 nm. Incubation time 2 minutes. Number of replicates *n* = 3. Control: no PPX was added in the reaction mixture, PPX = reaction of corresponding polyP with PPX. For polydisperse polyphosphates, the applied concentration is indicated based on P_*i*_ units.

Next, we investigated, if ESIPT sensors would enable us to follow polyP digestion by the enzyme exopolyphosphatase (PPX) *in vitro*. PPX assays are widely applied in polyP research. PPX digests long-chain polyphosphate releasing P_*i*_ units and one equivalent of PP_*i*_ per chain.^1*d*^ Thus, the digestion of polyP chains can potentially be monitored using the sensors 6 or 7, as P_*i*_ and PP_*i*_ only elicited no or only a little response by releasing 4 or 5, respectively (Fig. 4). Using this rationale, we investigated the application of the more sensitive probe 6, due to its higher response, towards monitoring polyP digestion by PPX. PPX (2.9 mg mL^−1^, 40 mM Tris-acetate, 10 mM Mg(OAc)_2_, 30 mM NH_4_OAc, 0.2 mM EDTA at pH = 8) was added to an aqueous solution of polyphosphates of varying chain length (2–10 mM, for polydisperse polyphosphates the concentration is based on P_*i*_ units) and incubated at 37 °C for 21 h. The enzyme reaction mixture as well as the control (no PPX) were added separately to a solution of 2.5 μM of 6 (in 10 mM HEPES buffer at pH = 7.4), and the ensuing fluorescence intensity at 434 nm was measured as the mean of three replicates in a 96 well-plate (details in ESI Section 5†). When the control mixture (only polyP_8_, no PPX) was incubated with probe 6, a clear increase in emission was observed. However, no significant increase in the emission intensity was observed when incubated with the PPX reaction mixture, because of the digestion of polyP_8_ by PPX to 6 P_*i*_ units and one PP_*i*_ (Fig. 7a and b).

PolyP digestion was also monitored for polydisperse polyphosphates (polyP_22_, polyP_60_, and polyP_130_). A significant decrease in fluorescence emission after digestion was observed (Fig. 7c–e). It is apparent that for polyP_8_ the difference of emission between control and PPX digestion is lower than that obtained for longer polyPs. This can be rationalized by the fact that digestion of polyP_8_ results in a higher PP_*i*_ : P_*i*_ ratio as compared to the digestion of longer chain-length polyPs that release less PP_*i*_ overall. Since the sensor shows no response to P_*i*_, but a small response to PP_*i*_, the larger difference can therefore be rationalised.

## Conclusions

In summary, two novel fluorescent ESIPT turn-on sensors have been successfully designed. They specifically detect short-chain inorganic polyphosphates over a range of different other condensed phosphates in aqueous solution. They operate through release of a metal ion quenched fluorophore by decomplexation of Cu^2+^ with the analyte. The probe preferentially detects polyPs over other relevant biologically occurring phosphates, pyrophosphates and nucleotides like ATP, ADP, GTP. An optimal fluorescence response has been achieved specifically in presence of polyP_8_. Additionally, the probe showed selectivity towards linear polyPs over other comparable cyclic metaphosphates. The sensor can cycle in ‘turn-off–turn-on’ fluorescence experiments upon consecutive additions of Cu^2+^ and polyP_8_, underscoring its reversible and sensitive performance in an aqueous environment. Furthermore, sensor 6 is suitable to monitor polyP digestion by exopolyphosphatase PPX in an endpoint experiment. Sensors 6 and 7 are the first tailored ESIPT-ON decomplexation sensors that operate in water to selectively detect short-chain polyphosphates. Their further development into vacuolar sensors thus seems feasible and will be the topic of follow-up studies. Short-chain polyphosphates may have escaped previous detection approaches, as the usually applied DAPI and JC-D dyes are only responsive to chain lengths beyond 15.^1*d*^ These novel sensors may thus close an important gap in current polyP research, where detection can still be quite limiting,^9^ and inspire the generation of tailored derivatives with improved sensing properties.

## Author contributions

S. K. R. synthesized the probes and evaluated their photophysical properties. S. K. R. performed the PPX assay. S. K. R. wrote the first draft of the manuscript and prepared the figures. S. M. synthesized the linear polyphosphates. T. D. M. synthesized the metaphosphates. R. H. developed initial sensor concepts. H. J. J. conceived the project and provided feedback on the draft manuscript. All authors contributed to finalizing the manuscript.

## Data availability

Additional datasets are available in the ESI and from the corresponding author upon request. The X-ray crystallography data have been deposited at the Cambridge Crystallographic Data Centre (CCDC), under accession number CCDC 2352054 and 2361756.†

## Conflicts of interest

There are no conflicts to declare.

## Supplementary Material

OB-023-D4OB01926A-s001

OB-023-D4OB01926A-s002
