# Supplementary material for: ESIPT fluorescence turn-on sensors for detection of short chain inorganic polyphosphate in water
Source: Org Biomol Chem. 2024 Dec 18;23(6):1373–9. doi: 10.1039/d4ob01926a (PMC11665774; doi:10.1039/d4ob01926a)

ESIPT fluorescence turn-on sensors for detection of short chain inorganic polyphosphate in water

Subhra Kanti Roy, Sandra Moser, Tobias Dürr-Mayer, Rahel Hinkelmann, and Henning J. Jessen

Table of Content:

Abbreviations 2

1. General experimental remarks 3
2. Spectroscopic studies: PolyP_8 sensing
   - 2.1 Absorption measurement 4
   - 2.2 Fluorescence measurement 4
   - 2.3 Fluorescence quantum yield 4
3. Emission measurement with different phosphate analytes on plate reader 6
4. Reversibility experiment 6
5. Polyphosphate digestion by exopolyphosphatase enzyme reaction monitoring 6
6. Supporting data for compound **5**
   - 6.1 Absorption titration spectra of **5** with Cu^{2+} and excitation spectra of **4** and **5** 7
   - 6.2 Fluorescence titration spectra of **5** with Cu^{2+} 7
7. Change of emission from fluorescence titration of **7** with polyP_8 8
8. Change of emission of **6** in presence of polyP_8 with time 8
9. Limit of Detection 9
10. HRMS spectra of **6** and **7** 10
11. Synthesis of the ESIPT probes 11
12. Synthesis of the polyphosphates 13
13. Crystallographic data 16
14. Supporting references 54
15. NMR and HRMS spectra 55

Abbreviations:

Å	Angstrom
aq.	aqueous
c-PyPA	*cyclic* pyrophosphoryl phosphoramidite
DBU	1,8-diazabicyclo[5.4.0]undec-7-ene
DMSO	dimethylsulfoxide
DCM	dichloromethane
EDTA	ethylenediaminetetraacetic acid
equiv.	equivalent
EtOH	ethanol
ESI	electrospray ionization
ETT	5-(ethylthio)-1*H*-tetrazole
Fm	(9*H*-fluoren-9-yl)methyl
HEPES	4-(2-hydroxyethyl)-1-piperazineethanesulfonic acid
HRMS	high resolution mass spectrometry
nm	nanometre
m	multiplet (NMR), milli
M	molar
µM	micromolar
*m*CPBA	*meta*-chloroperoxybenzoic acid
MeCN	acetonitrile
NMR	nuclear magnetic resonance
Pi	phosphate
PPi	pyrophosphate
polyP	polyphosphate(s)
ppm	parts per million
rpm	revolutions per minute
RP-MPLC	reverse phase medium pressure liquid chromatography
TBA	tetrabutylammonium
THF	tetrahydrofuran

1. General experimental remarks

Reactions were carried out using glassware magnetically stirred, unless noted otherwise. Air-and moisture-sensitive liquids and solutions were transferred via syringe. Reagents were purchased from commercial suppliers (Sigma Aldrich, Acros, TCI, Roth, ChemPur, Alfa Aesar, VWR/Merck) and used as received unless noted otherwise.

Solvents were obtained in analytical grade and used as received for extractions, precipitation and solid washing.

Dry solvents for reactions were purchased in a dry form from Sigma and stored over molecular sieves as well as under the atmosphere of dry N_2.

Deuterated solvents for NMR and reactions were obtained from Armar Chemicals, Switzerland and Euriso-top, Germany, in the indicated purity grade and used as received for NMR spectroscopy.

Phosphates: Pi, PPi, P_3, trimetaphosphate were commercially bought from Sigma-Aldrich and Acros. $PolyP_{14}$, $polyP_{60}$, $polyP_{100}$ and $polyP_{130}$ were obtained from RegeneTiss Inc. $polyP_{22}$, $polyP_{45}$, where obtained from Aldrich (polyP glass). $PolyP_{700}$ was bought from kerafast**.**

Lyophilizations were done with Christ Freeze Dryer Alpha 1-4 LDplus and Christ Freeze Dryer Alpha 1-2 LDplus.

Preparative RP-MPLC was performed using an automated Interchim® - system. The C18AQ-solid phase was purchased from Interchim.

1H-NMR spectra were recorded on Bruker 300 MHz spectrometers, Bruker 400 MHz (with cryoprobe) and Bruker 500 MHz spectrometers in the indicated deuterated solvent. Data are reported as follows: chemical shift (δ, ppm), multiplicity (s, singlet; d, doublet; t, triplet; q, quartet; m, multiplet; br. s, broad signal), coupling constant(s) (J, Hz), integration. All signals were referenced to the internal solvent signal as standard (D_2O: δ0= 4.79, DMSO-d_6: δ= 2.50, $CDCl_3$: δ= 7.26 ppm).

13C{1H}-NMR spectra were recorded with 1H-decoupling on Bruker 126 MHz, Bruker 101 MHz (with cryoprobe) spectrometers at 298K in the indicated deuterated solvent. If possible, signals were referred to the internal solvent signal as standard (DMSO-d6: δ= 39.52, $CDCl_3$: δ = 77.23 ppm).

X-ray crystallography: Crystals were mounted on a MiTeGen micromount with perfluoroether oil. Data were collected from a shock-cooled single crystal at 100(2) K on a Bruker D8 VENTURE dual

wavelength Mo/Cu three-circle diffractometer with a microfocus sealed X-ray tube using a mirror optics as monochromator and a Bruker PHOTON III detector. The diffractometer was equipped with an Oxford Cryostream 800 low temperature device and used MoKα radiation (λ = 0.71073 Å). All data were integrated with SAINT and a multi-scan absorption correction using SADABS was applied. The structure was solved by direct methods using SHELXT and refined by full-matrix least-squares methods against F2 by SHELXL-2019/2. All non-hydrogen atoms were refined with anisotropic displacement parameters. All C-bound hydrogen atoms were refined with isotropic displacement parameters. Some of their coordinates were refined freely and some on calculated positions using a riding model with their Uiso values constrained to 1.5 times the Ueq of their pivot atoms for terminal sp^3 carbon atoms and 1.2 times for all other carbon atoms.

UV/Vis-Absorption spectra were recorded on UV Vis Spectrometer Shimadzu 1900i.

Fluorescence Spectra were recorded on a JASCO FP-8200 Spectrofluorometer or on a Tecan Spark 10M Microplate Topreader using 96-well plates (Thermo Fisher Scientific-Nunclon 96 Flat Black).

2. Spectroscopic studies: PolyP$_8$ sensing:

2.1 Absorption measurement experiments. All absorption measurements were performed from a 100 µM stock solution of the probes (final concentration 5 µM in 10 mM HEPES buffer; pH =7.4) with addition of sodium salt of the polyP$_8$ from a stock solution of 2 mM (aq.). Total volume of the measurement solutions were 2 ml. The absorptions were recorded after 2 minutes of the addition of the analytes into the solution of the probes.

2.2 Fluorescence measurement experiments: All fluorescence measurements were performed from a 100 µM stock solution of the probes (final concentration 2.5/5 µM in 10 mM HEPES buffer, pH=7.4) with addition of sodium salt of the polyP$_8$ a stock solution of 2 mM (aq.). Total volume of the measurement solutions were 1.5 ml. The fluorescence were recorded after 2 minutes of the addition of the analytes into the solution of the probes.

2.3 Fluorescence quantum yield:

Fluorescence Quantum Yield was measured on JASCO Spectrofluorometer FP-8300 using integration shepre ILF-835 with liquid sample cell (1 mm thickness, 1 x 10 x 25 mm). In this program, the quantum yield is determined using the excitation light spectrum (incident light spectrum) and the

sample emission spectrum measured under identical conditions. (From [Quantum Yield Calculation] Program Software Manual).

$$\text{Internal quantum efficiency} = \frac{\text{The number of photons in emission}}{\text{The number of photons in the excitation light absorbed by the sample}}$$

To calculate the fluorescence quantum efficiency of a compound three emission spectra were measured. (1) Incident light spectrum, i.e. emission spectrum by excitation light irradiated to the inner surface of the integrating sphere without the sample, (2) Direct spectra i.e. sample spectrum, emission spectrum by excitation light directly irradiate to the sample. The spectrum contains both excitation light and emission light. (3) Indirectly excited sample spectrum, i.e. emission spectrum by excitation light irradiated to the inner surface of the integrating sphere. The sample is indirectly excited by scattered light. This spectrum is used for indirect excitation correction.

These three spectra were utilized in the Quantum Yield Calculation Program provided by the instrument's software.

Table S1: Fluorescence quantum yield.

Compound	Absorptance (%)	Ext. Quantum Efficiency (%)	Int. Quantum Efficiency (%)
**4**	70.052	23.198	33.116
**5**	35.226	5.919	16.804

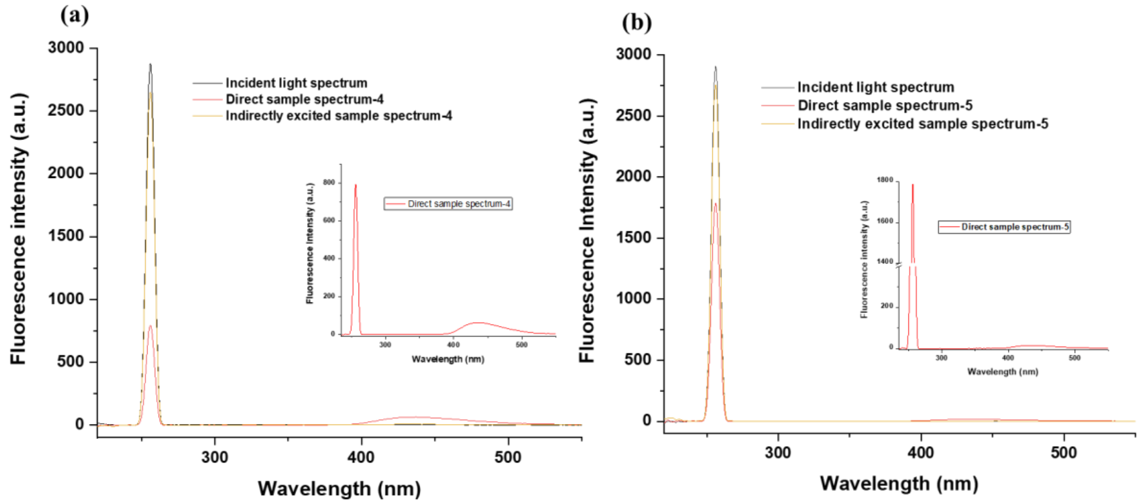

Figure S1: Emission spectra of (a) **4** and (b) **5** with Incident light spectrum, Direct sample spectrum and Indirectly excitated sample spectrum. Measurement range 220-700 nm, Ex wavelength 254 nm and 250 nm for **4** and **5** respectively.

3. Emission measurement with different phosphate analytes on a plate reader: Emission intensity of the probes with different analytes (from a stock solution of 2 mM in water) were recorded in 10 mM HEPES buffer solution at pH 7.4 on Thermo Scientific™ 96-well plates, in triplicates, with a total volume of 150 µL. The emission were recorded after 2 minutes of the addition of the analytes to the solution of the probes.

4. Reversibility experiment: Cycle 1 is the initial fluorescence of **4** (5 µM, in 10 mM HEPES buffer, at pH 7.4). Reversibility has been accomplished by adjustment of the alternative addition Cu (II) and polyP8 in different portions to the solution of **4** (5 µM, in 10 mM HEPES buffer, at pH 7.4) as depicted below. The emission were recorded after 2 minutes of the addition of the Cu^{2+}/polyP$_8$ to the solution of the probes.

Number of cycle	Equivalent
1	-
2	0.5 equiv. Cu^{2+}
3	1 equiv. polyP$_8$
4	1 equiv. Cu^{2+}
5	1.5 equiv. polyP$_8$
6	2.5 equiv. Cu^{2+}
7	2.5 equiv. polyP$_8$
8	4 equiv. Cu^{2+}
9	4 equiv. polyP$_8$
10	4 equiv. Cu^{2+}

5. Polyphosphate digestion by exopolyphosphatase enzyme reaction monitoring:
5.5 µL of 2.9 mg/ml Exopolyphosphatase in buffer solution of 40 mM Tris-acetate, 10 mM $Mg(OAc)_2$, 30 mM NH_4OAc, 0.2 mM EDTA, at pH 8 was added to the 50 µL different polyphosphate solutions in miliQ water (2 mM of polyP$_8$, 1 mg/mL of polyP$_{22}$, 4 mM of polyP$_{60}$ and 10 mM of polyP$_{130}$ were used for digestion). The solution was incubated for 21 h at 37 ^{0}C using a shaker at 600 rpm. The reaction mixture was quenched by freezing the solution with liquid nitrogen followed by centrifugation at 4 ^{o}C for 5 minutes with a speed of 13500 g.
Total well volume of all emission measurements were 75 µL. For polyP8-PPX and polyP22-PPX 10 µL, and for polyP60-PPX and polyP130-PPX reaction 20 µL reaction mixture were added into probe

6 (2.5 μM, 10 mM HEPES buffer, at pH 7.4). The consequent fluorescence intensities at 434 nm were measured as the mean of three replicates on a black 96 well- plate after 2 minutes the additions.

6.1 Absorption titration spectra of 5 with Cu^{2+} and excitation spectra of 4 and 5:

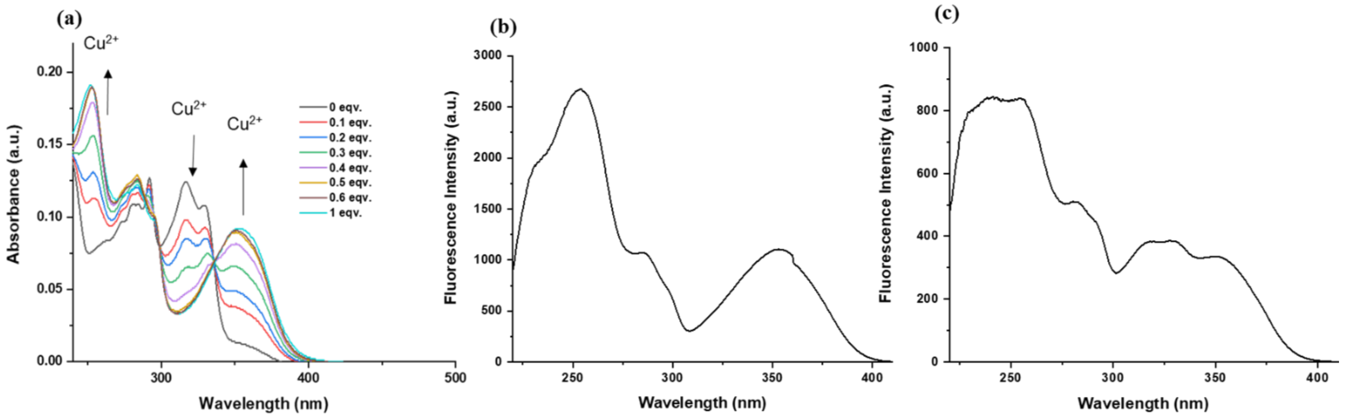

Figure S2: (a) Absorption titration spectra of **5** (10 μM) in HEPES-buffer (10 mM, pH= 7.4) in the presence of increasing concentrations of Cu^{2+} (0–1 equiv.). Excitation spectra of (b) **4** (2.5 μM) and (c) **5** (5μM) in HEPES buffer (10 mM, pH= 7.4).

6.2 Fluorescence titration spectra of 5 with Cu^{2+}:

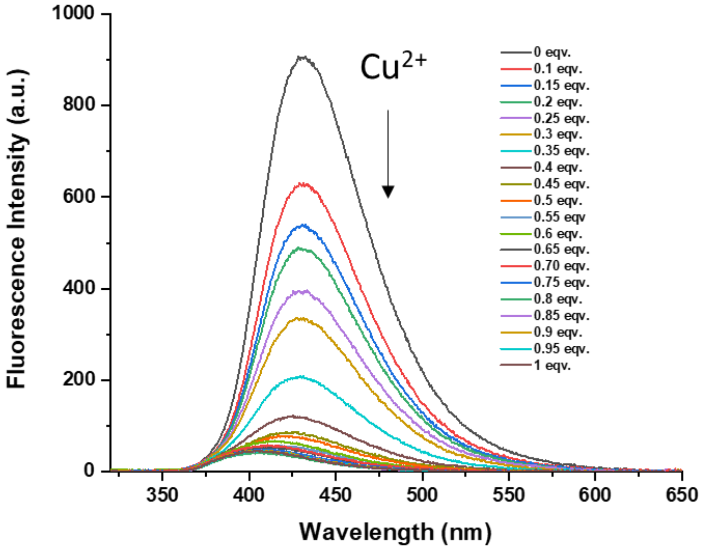

Figure S3: Fluorescence titration spectra of **5** (5 μM) in HEPES-buffer (10 mM, pH= 7.4) in the presence of increasing concentrations of Cu^{2+} (0–1.2 equiv.). Excitation wavelength= 250 nm.

7. **Change of emission from fluorescence titration of 7 with polyP$_8$:**

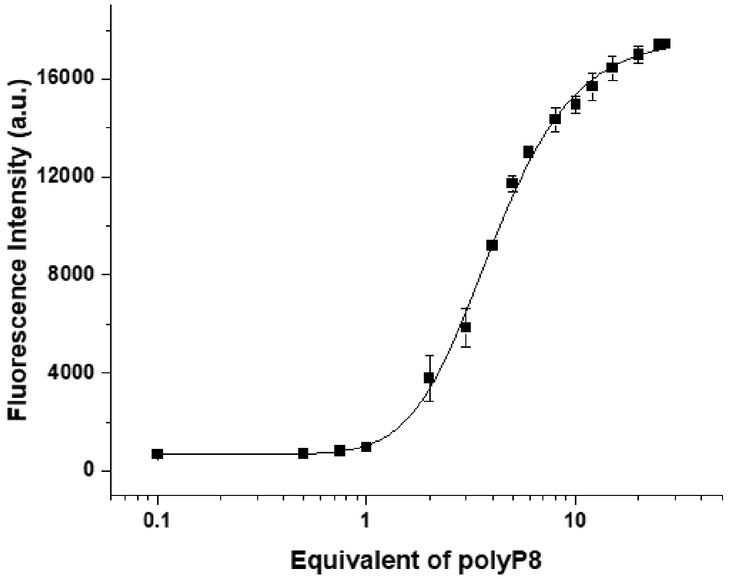

Figure S4: Change of emission intensity of 5 µM of **7** (10 mM HEPES-buffer, pH= 7.4) at 430 nm with increasing polyP$_8$ concentration. Excitation wavelength= 250 nm. Number of replicates is three.

8. Change of emission of 6 in presence of polyP$_8$ with time:

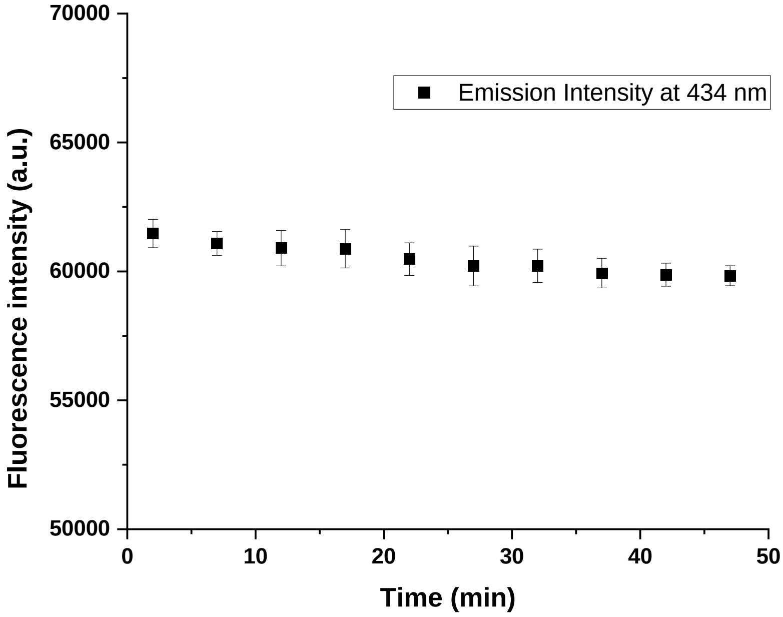

Figure S5: Change of emission intensity of 2.5 µM of **6** (10 mM HEPES-buffer, pH= 7.4) at 434 nm in presence of 10 equiv. of polyP$_8$ with time. Excitation wavelength= 254 nm. Number of replicates is three.

9. Limit of Detection: The detection limit (LOD) was determined from the fluorescence titration data based on a reported method.[1] The fluorescence emission of probes were measured in a triflicate and the standard deviation of blank measurement was calculated. The emission intensity data at 434 nm for **6** and 430 nm for **7** were plotted as concentration of polyP8 to get the slope. The LODs were calculated with the following equation:

Limit of detection = 3σ/K

σ= standard deviation of blank measurement, and K = slop between the fluorescence and polyP8 concentration.

LOD of $polyP_8$ with **6**= 97 nM; LOD of $polyP_8$ with **7**= 110 nM.

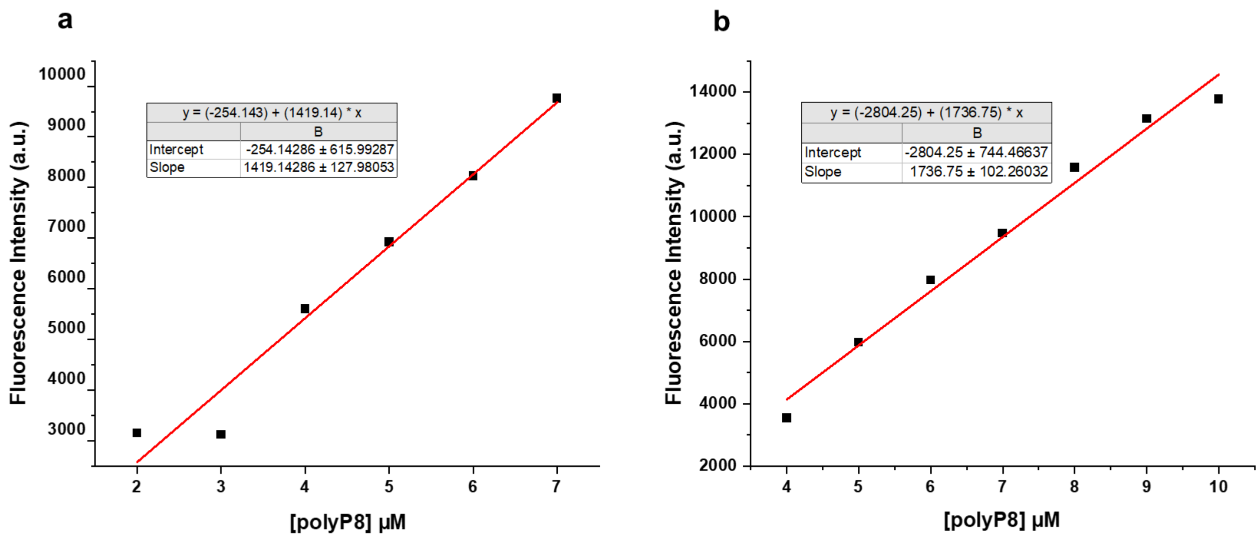

Figure S6: Emission intensity of (a) 2.5 µM **6** at 434 nm (Ex= 254 nm) and (b) 5 µM **7** at 430 nm (Ex= 250 nm) in10 mM HEPES-buffer, pH= 7.4 with $polyP_8$ concentration.

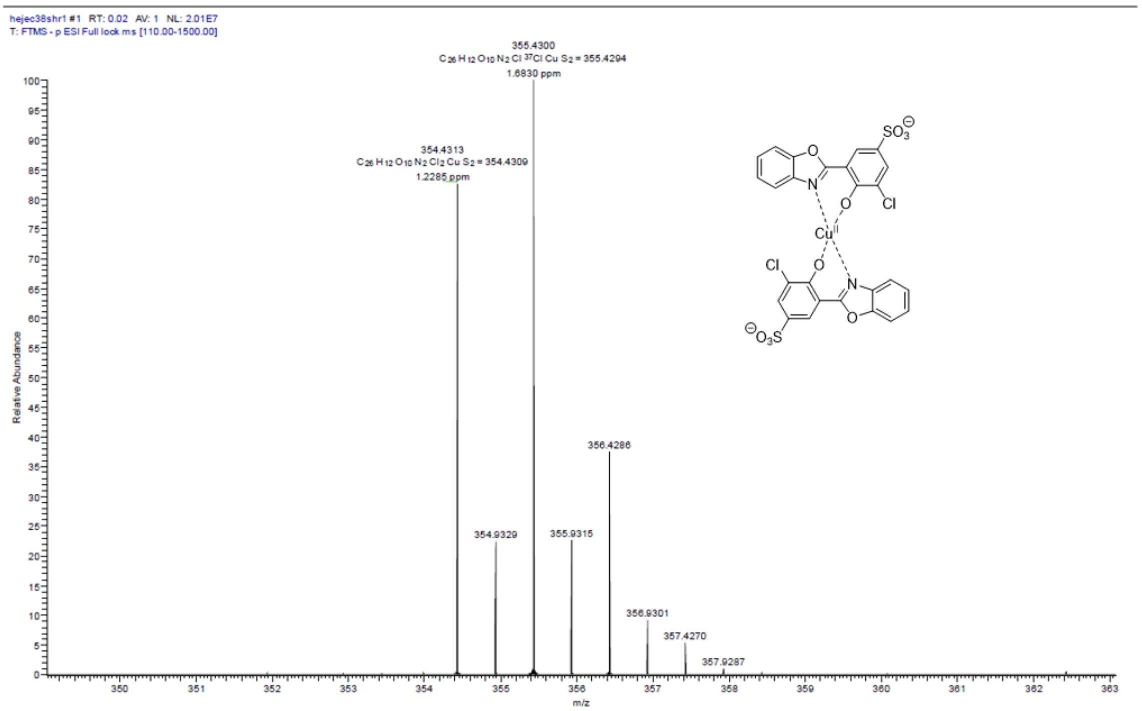

Figure S7: HRMS spectrum of **6**. HRMS (ESI) exact mass calculated for $C_{26}H_{12}Cl_2CuN_2O_{10}S_2^{2-}$ ([M-Na]$^{2-}$): 354.4309; Found: 354.4313.

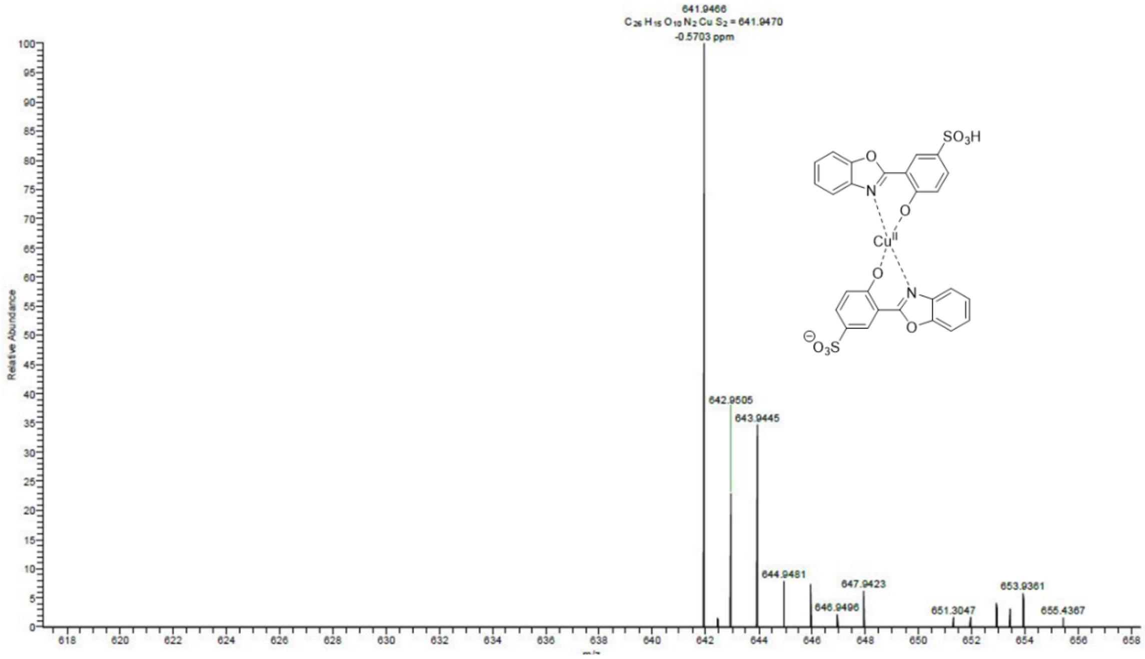

Figure S8: HRMS spectrum of **7**. HRMS (ESI) exact mass calculated for $C_{26}H_{15}CuN_2O_{10}S^-$ ([M+H-Na]): 641.9470; Found: 641.9466.

11. Synthesis of the ESIPT probes 6 and 7:

2-chloro-6-((phenylimino)methyl)phenol (1): A solution of aniline (0.16 mL, 1.92 mmol) in methanol (1 ml) was added to a solution of 3-chloro-salicylaldehyde (0.3 g, 1.92 mmol) in methanol (7.5 mL) and the reaction mixture was refluxed for 4 h. The reaction mixture was allowed to cool to room temperature and the solvent was evaporated under vacuum to obtain brown gummy residue which became solid after10-15 min at room temperature. The residue was washed with with cyclohexane and dried in under vacuum to get **1** as orange solid (0.44 g, 1.9 mmol, 99%) which was directly used for the next step without any purification. **^{1}H-NMR** (300 MHz, $CDCl_3$) δ= 8.62 (s, 1H), 7.48 – 7.42 (m, 3H), 7.35 – 7.29 (m, 4H), 6.89 (t, J = 7.8 Hz, 1H) ppm. **^{13}C-NMR** (101 MHz, $CDCl_3$) δ= 161.8, 157.4, 147.6, 133.4, 130.8, 129.8, 127.7, 122.1, 121.4, 120.2, 119.4 ppm. **HRMS** (ESI) exact mass calculated for $C_{13}H_{11}ClNO$ ($[M+H]^+$): 232.0524; Found: 232.0522.

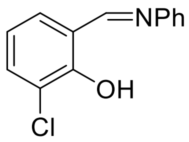

Sodium 3-chloro-5-formyl-4-hydroxybenzenesulfonate (3): Concentrated sulphuric acid (5 mL) (95 wt %, d = 1.84 g/mL) was added slowly to the imine (0.44 g, 1.9 mmol) and the mixture was heated to105 ^{o}C with a reflux condenser for 2.5 h. The reaction mixture was cooled and the solution was added slowly into a beaker containing ice water. Precipitation was observed after keeping the solution in an ice bath for 30-45 minutes. The brownish black precipitate was collected by filtration and dried under vacuum to get **2** (0.5 g, 1.6 mmol, 84%) as a brown solid which was used for the next step without any purification. *N*-Phenyl-5-sulfonato-salicylaldimine **2** was dissolved in H_2O (6 mL) and Na_2CO_3 (0.2 g, 1.92 mmol) was added slowly to the solution. The reaction mixture was heated vigorously in an open flask for 2.5 h (during the reaction time the water was replenished if required). The reaction mixture was cooled and kept in an ice bath. Glacial acetic acid (2 ml) was added to the cooled solution and afterwards EtOH (2 ml) was added to the solution. The solution was kept in an ice bath for several hours to obtain a yellow precipitation ,which was filtered and dried under vacuum. The yellow solid was finally purified by RP-MPLC (column: interchim® PF-30C18AQ, F0040; λ = 254 nm; with 1-10 % acetonitrile in water, flow rate 26 mL/min) to obtain **3** as a light yellow solid (0.24 g, 0.93 mmol, 58%). **^{1}H-NMR** (400 MHz, D_2O) δ= 10.05 (s, 1H), 7.83 – 7.80 (m, 2H) ppm. **^{13}C-NMR** (101 MHz, D_2O) δ= 191.7, 168.7, 129.8, 126.1, 124.1, 123.4, 121.7 ppm. **HRMS** (ESI) exact mass calculated for $C_7H_4ClO_5S^-$ ($[M-Na]^-$): 234.9473; Found: 234.9475.

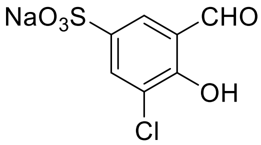

Sodium 3-(benzo[*d*]oxazol-2-yl)-5-chloro-4-hydroxybenzenesulfonate (4): *Sodium 3-chloro-5-formyl-4-hydroxybenzenesulfonate* **3** (0.19 g, 0.73 mmol) was dissolved in MeOH (8 ml) and 2-aminophenol (80 mg, 0.74 mmol) was added to the solution. The mixture was refluxed for 3 h and completion of the reaction was monitored by TLC. The reaction mixture was allowed to cool to room temperature and the solvent was evaporated to dryness under reduced pressure to get the intermediate imine as a red solid residue. The solid imine was dissolved in MeOH (8 ml) and stirred with $PhI(OAc)_2$ at room temperature overnight. The solvent was evaporated under vacuum to obtain a dark red solid residue which was purified by RP-MPLC (column: interchim® PF-30C18AQ, F0025; λ = 254 nm; with 1-10 % acetonitrile in water, flow rate 15 mL/min) to afford analytically pure **4** as a brown red solid (98 mg, 0.3 mmol 41%). **^{1}H-NMR** (400 MHz, DMSO-d_6) δ= 11.99 (brs, 1H), 8.24 (d, *J* = 2.0 Hz, 1H), 7.93 - 7.90 (m, 2H), 7.80 (d, *J* = 2.0 Hz, 1H), 7.56 - 7.48 (m, 2H) ppm. **^{13}C-NMR** (101 MHz, DMSO) δ= 161.4, 153.3, 148.9, 141.1, 138.9, 130.7, 126.4, 125.6, 123.1, 120.3, 119.4, 111.4, 110.7 ppm. **HRMS** (ESI) exact mass calculated for $C_{13}H_7ClNO_5S^-$ ($[M-Na]^-$): 323.9739; Found: 323.9740.

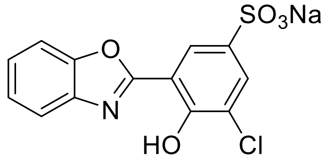

Sodium 3-(benzo[*d*]oxazol-2-yl)-4-hydroxybenzenesulfonate (5): Concentrated sulphuric acid (6 mL) (95 wt %, d = 1.84 g/mL) was added slowly into the 2-hydroxy-phenyl-benzoxazole (0.5 g, 2.36 mmol) and the mixture was heated at 105 ^{o}C with a reflux condenser for 2.5 h. The solution was cooled and added slowly into beaker containing ice water. Precipitation was observed after keeping the solution on ice bath for 30-45 minutes. The brown precipitate was collected by filtration and dried under vacuum to get **5** (0.68 g, 2.35 mmol) as a brown solid, which was dissolved in H_2O (10 mL) and Na_2CO_3 (0.3 g, 2.8 mmol) was added slowly to the solution. The reaction mixture was heated vigorously in an open flask for 2.5 h (During the reaction time the water was replenished if required). The reaction mixture was cooled and kept in an ice bath. Glacial acetic acid (6 mL) was added to the cooled solution and afterwards EtOH (2 mL) was added to the solution. The solution was kept in an ice bath for several hours to obtain a light yellow precipitate, which was filtered and dried on vacuum. The yellow solid was finally purified by RP-MPLC (column: interchim® PF-30C18AQ, F0040; λ = 254 nm; with 1-15 % acetonitrile in water, flow rate 26 mL/min) to obtain **5** as a colorless solid (0.38 g, 1.2 mmol, 52%). **^{1}H-NMR** (400 MHz, DMSO-d_6) δ= 11.24 (s, 1H), 8.29 (d, *J* = 2.4 Hz, 1H), 7.90 – 7.85 (m, 2H), 7.72 (dd, *J* = 8.8, 2.4 Hz, 1H), 7.51 – 7.45 (m, 2H), 7.08 (d, *J* = 8.4 Hz, 1H) ppm. **^{13}C-NMR** (101 MHz, DMSO) δ= 162.1, 157.6, 148.9, 140.6, 139.5, 131.3, 125.9, 125.3, 124.9, 119.2, 116.5, 111.1, 109.1 ppm. **HRMS** (ESI) exact mass calculated for $C_{13}H_8NO_5S$ ($[M-Na]^-$): 290.0129; Found: 290.0126.

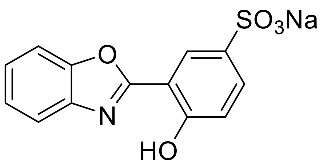

Synthesis of 3-(benzo[*d*]oxazol-2-yl)-5-chloro-4-hydroxybenzenesulfonate Cu (II) complex (6):

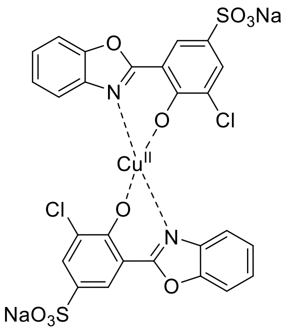

4 (30 mg, 0.09 mmol) was suspended in dry THF (1.5 mL) and an aqueous solution of $CuCl_2$ (6 mg in 1.5 mL H_2O) solution was added to the suspension. The reaction mixture was stirred at room temperature for 4 h. The suspension was filtered and the solid was washed with THF to obtain the complex **6** as a brown green solid (22 mg, 0.028 mmol, 61%). **HRMS** (ESI) exact mass calculated for $C_{26}H_{12}Cl_2CuN_2O_{10}S_2^{2-}$ ($[M-Na]^{2-}$): 354.4309; Found: 354.4313.

Synthesis of 3-(benzo[*d*]oxazol-2-yl)-4-hydroxybenzenesulfonate Cu (II) complex (7):

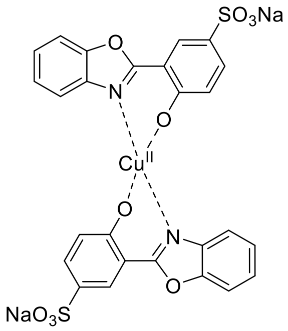

5 (44 mg, 0.14 mmol) was suspended in dry THF (2 mL) and an aqueous solution of $CuCl_2$ (9.4 mg in 2 mL H_2O) solution was added to the suspension. The reaction mixture was stirred at room temperature for 4 h. The suspension was filtered and the solid was washed with THF to obtain the complex **7** as a light green solid (24 mg, 0.32 mmol, 45%). **HRMS** (ESI) exact mass calculated for $C_{26}H_{15}CuN_2O_{10}S^-$ ([M+H-Na]): 641.9470; Found: 641.9466.

12. Synthesis of the polyphosphates:

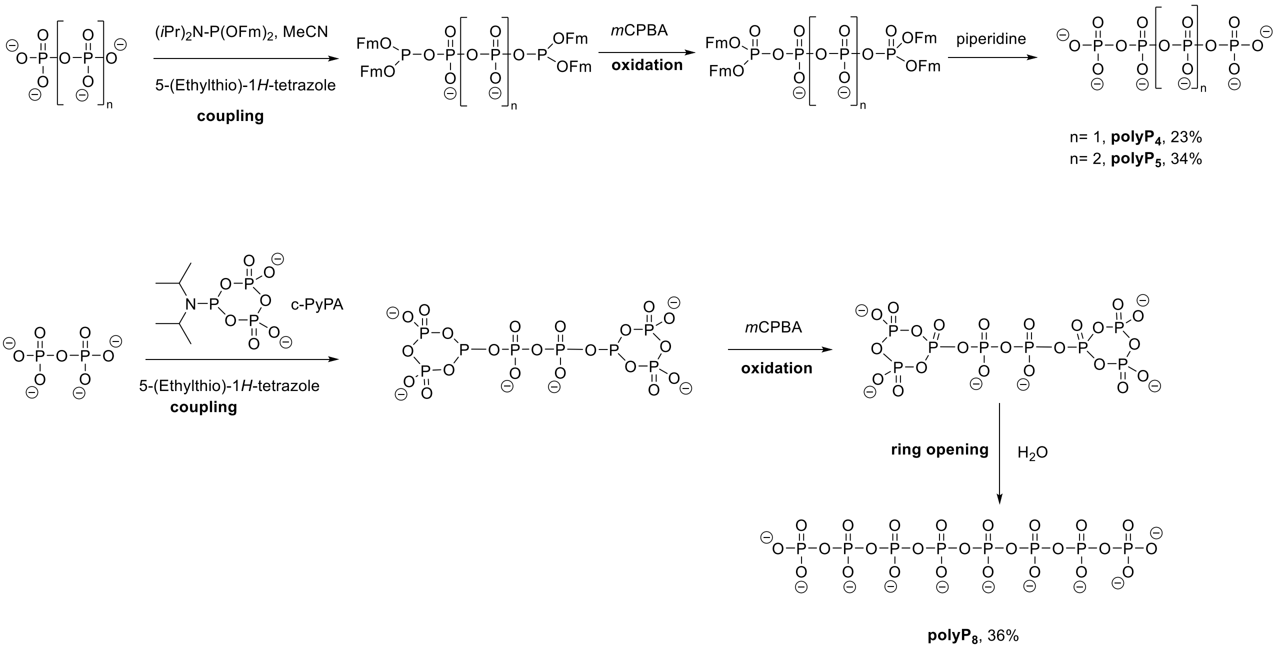

Scheme S1: Synthesis of polyphosphates with defined chain lengths.

PolyP$_4$:

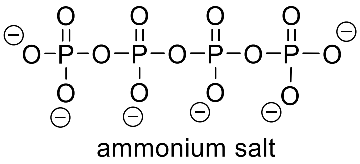

5-(Ethylthio)-1*H*-tetrazole (ETT) (294 mg, 2.26 mmol, 5.0 equiv.) and (*i*Pr)$_2$N-P(OFm)$_2$ (660 mg, 1.14 mmol, 2.5 equiv.) were separately coevaporated with dry MeCN (2 × 2 mL). Pyrophosphate × 2 TBA (0.075 M in MeCN, 6 mL, 0.450 mmol, 1.0 equiv.) was added to ETT followed by a solution of (*i*Pr)$_2$N-P(OFm)$_2$ in dry DCM (6 mL). The reaction mixture was stirred for 20 min. Subsequently the mixture was cooled to 0 °C and *m*CPBA (70%, 244 mg, 0.990 mmol, 2.2 equiv.) was added to the mixture. The reaction mixture was stirred for 20 min before piperidine (10V%, 1.2 mL) was added at 0 °C. The cooling bath was removed after 5 min and the mixture was stirred in total for 1 h. The crude product was precipitated by $NaClO_4$ (0.5 M in acetone), centrifuged, washed with acetone (3×10 mL) and purified by anion exchange chromatography (Q Sepharose® Fast Flow, increasing concentrations of aq. NH_4HCO_3). Fractions eluted with 300 mM aqueous NH_4HCO_3 were combined and lyophilized. To remove buffer, the product was dissolved again in water and lyophilized (2 ×10 mL). **PolyP$_4$** ammonium salt (45 mg, 0.102 mmol, 23%) was obtained as a white powder.

^{31}P-NMR (162 MHz, D_2O) δ = -8.20 – -8.61 (m, 2P), -22.28 – -22.73 (m, 2P) ppm. **HRMS CE-ESI** calculated for $H_5O_{13}P_4$ [M-H]$^-$: 336.8686, found: 336.8660.

PolyP$_5$:

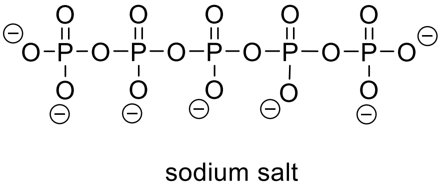

5-(Ethylthio)-1*H*-tetrazole (439 mg, 3.37 mmol, 5.0 equiv.) and (*i*Pr)$_2$N-P(OFm)$_2$ (880 mg, 1.43 mmol, 2.1 equiv.) were separately coevaporated with dry MeCN (2 × 2 mL). Triphosphate × 3 TBA (0.075 M in MeCN, 9 mL, 0.675 mmol, 1.0 equiv.) was added to ETT followed by a solution of (*i*Pr)$_2$N-P(OFm)$_2$ in dry DCM (20 mL). The reaction mixture was stirred for 20 min. Subsequently the mixture was cooled to 0 °C and *m*CPBA (70%, 366 mg, 1.49 mmol,

2.2 equiv.) was added to the mixture. The reaction mixture was stirred for 25 min before DBU (14 V%, 4 mL) was added at 0 °C. The mixture was stirred for 2 h and was allowed to reach room temperature. The crude product was precipitated by cold ether, centrifuged, washed with cold ether (2 × 15 mL) and purified by anion exchange chromatography (Q Sepharose® Fast Flow, increasing concentrations of aq. $NaClO_4$). Fractions eluted with 100-150 mM aq. $NaClO_4$ were combined and lyophilized, dissolved again in water and precipitated and washed with cold ether. **PolyP$_5$** sodium salt (130 mg, 0.228 mmol, 34%) was obtained as a white powder.

^{31}P-NMR (162 MHz, D_2O) δ = -9.07 – -9-64 (m, 2P), -21.99 – -22.48 (m, 3P) ppm. **HRMS CE-ESI** calculated for $H_7O_{16}P_5$ [M-H]$^-$: 416.8360, found: 416.8359.

PolyP$_8$:

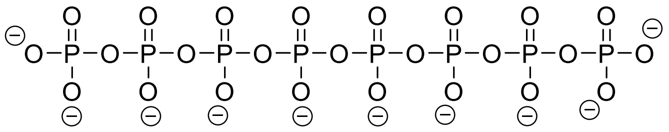

sodium salt

5-(Ethylthio)-1*H*-tetrazole (686 mg, 5.27 mmol, 7.0 equiv.) was coevaporated with dry MeCN (3 × 3 mL). Pyrrophosphate × 2 TBA in MeCN (0.15 M, 5.0 mL, 0.75 mM, 1.0 equiv.) was added and the reaction mixture was stirred for 5 min at room temperature. The reaction mixture was cooled to -20 °C, *c*-PyPA (0.11 M, 20 mL, 2.2 mmol, 2.9 equiv.) was added and stirred for 55 min (cooling bath removed after 10 min). *m*CPBA (70%, 737 mg, 2.99 mmol, 4.0 equiv.) was added at -10 °C in one portion and the reaction mixture was stirred for 40 min (cooling bath removed after 20 min). The reaction mixture was dropped into water (60 mL) at room temperature and the solution was stirred for 80 min. The reaction mixture was slowly dropped into cold 0.5 M $NaClO_4$ in acetone. The formed precipitate was isolated via centrifugation. The crude product was washed with cold acetone (3 × 15 mL) and dried. Purification was performed by anion exchange chromatography (QSepharose® Fast Flow, increasing concentration of aq. $NaClO_4$-solution (1M)). Product containing fractions were combined and lyophilized, precipitated with cold $NaClO_4$ acetone solution (0.5 M) and washed with cold acetone (3 ×15 mL). **PolyP$_8$** sodium salt (235 mg, 0.268 mmol, 36%) was obtained as a white powder.

^{31}P-NMR (162 MHz, D_2O) δ = -8.69 – -10.56 (m, 2P), -21.93 – -22.37 (m, 6P) ppm. **HRMS CE-ESI** calculated for $H_{10}O_{25}P_8$ [M-2H]$^{2-}$: 327.8639, found: 327.8637.

13. Crystallographic data:

Crystallographic data of 4:

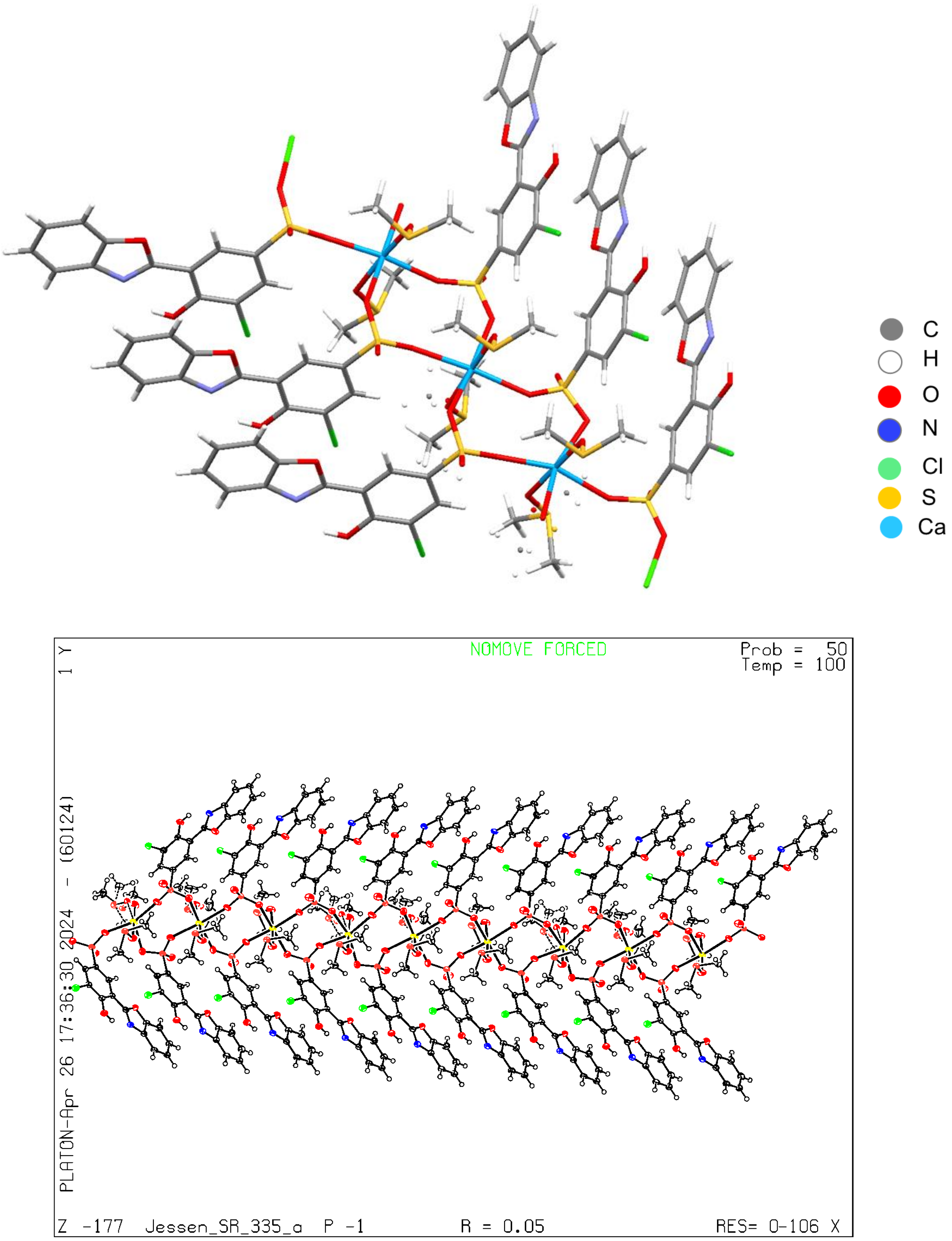

Figure S9: Crystal structure of **4**.

During crystallization process, crystal of Ca^{2+} salt of **4** was obtained. Crystals were obtained at room temperature by layering of a solution in acetonitrile with methanol. A colourless, needle-shaped crystal of **4** was mounted on a MiTeGen micromount with perfluoroether oil. Data were collected from a shock-cooled single crystal at 100(2) K on a Bruker D8 VENTURE dual wavelength Mo/Cu three-circle diffractometer with a microfocus sealed X-ray tube using a mirror optics as monochromator and a Bruker PHOTON III detector. The diffractometer was equipped with an Oxford Cryostream 800 low temperature device and used MoKα radiation (λ = 0.71073 Å). All data were integrated with SAINT and a multi-scan absorption correction using SADABS was applied.[2,3] The structure was solved by direct methods using SHELXT and refined by full-matrix least-squares methods against F2 by SHELXL-2019/2.[4,5] All non-hydrogen atoms were refined with anisotropic displacement parameters. All C-bound hydrogen atoms were refined isotropic on calculated positions using a riding model with their Uiso values constrained to 1.5 times the Ueq of their pivot atoms for terminal sp^3 carbon atoms and 1.2 times for all other carbon atoms. Disordered moieties were refined using bond lengths restraints and displacement parameter restraints. Some parts of the disorder model were introduced by the program DSR.[6,7] Crystallographic data for the structures reported here have been deposited with the Cambridge Crystallographic Data Centre.[8] CCDC 2352054 contain the supplementary crystallographic data for this paper. These data can be obtained free of charge from The Cambridge Crystallographic Data Centre via www.ccdc.cam.ac.uk/structures. This report and the CIF file were generated using FinalCif.[9]

Table S2. Crystal data and structure refinement for **4**

CCDC number	2352054
Empirical formula	$C_{90}H_{78}Ca_3Cl_6N_6O_{36}S_{12}$
Formula weight	2537.24
Temperature [K]	100(2)
Crystal system	triclinic
Space group (number)	$P\bar{1}$ (2)
a [Å]	15.316(4)
b [Å]	18.777(6)
c [Å]	21.258(6)
α [°]	104.191(7)
β [°]	108.995(12)
γ [°]	106.901(9)
Volume [Å^3]	5126(3)
Z	2
ρ_{calc} [gcm^{-3}]	1.644
μ [mm^{-1}]	0.651

$F(000)$	2604
Crystal size [mm^3]	0.198×0.029×0.029
Crystal colour	colourless
Crystal shape	needle
Radiation	MoK_α (λ=0.71073 Å)
2θ range [°]	2.45 to 61.29 (0.70 Å)
Index ranges	−21 ≤ h ≤ 21<br>−26 ≤ k ≤ 26<br>−30 ≤ l ≤ 30
Reflections collected	254457
Independent reflections	31429<br>R_{int} = 0.0924<br>R_{sigma} = 0.0754
Completeness to<br>θ = 25.242°	100.0 %
Data / Restraints / Parameters	31429/320/1480
Absorption correction<br>T_{min}/T_{max} (method)	0.6718/0.7461<br>(multi-scan)
Goodness-of-fit on F^2	1.011
Final R indexes<br>[I≥2σ(I)]	R_1 = 0.0548<br>wR_2 = 0.1319
Final R indexes<br>[all data]	R_1 = 0.1101<br>wR_2 = 0.1605
Largest peak/hole [eÅ^{-3}]	1.34/−0.72

Table S3. Atomic coordinates and U_{eq} [Å^2] for **4**

Atom	*x*	*y*	*z*	U_{eq}
Ca1	0.26506(4)	0.24400(3)	0.49221(3)	0.01861(11)
Ca2	0.60973(4)	0.25255(3)	0.49339(3)	0.02225(12)
Ca3	0.90553(4)	0.23486(3)	0.48812(3)	0.02230(12)
O1_1	0.6626(8)	0.2143(6)	0.5902(6)	0.0249(16)
S1_1	0.6681(4)	0.1357(4)	0.5904(4)	0.0217(8)
C1_1	0.6986(8)	0.1398(5)	0.6780(3)	0.060(2)
H1A_1	0.648596	0.150917	0.693369	0.090
H1B_1	0.765695	0.182537	0.709895	0.090
H1C_1	0.698690	0.088191	0.679887	0.090
C2_1	0.5461(5)	0.0566(4)	0.5471(4)	0.055(2)
H2A_1	0.513078	0.052202	0.497511	0.083
H2B_1	0.505741	0.067924	0.572726	0.083
H2C_1	0.551865	0.006036	0.547225	0.083
O1_2	0.6442(10)	0.2039(6)	0.5829(7)	0.041(2)
S1_2	0.6609(5)	0.1294(5)	0.5910(4)	0.0394(14)
C1_2	0.7687(5)	0.1679(4)	0.6751(3)	0.0391(17)
H1A_2	0.770583	0.124444	0.692629	0.059
H1B_2	0.764280	0.210209	0.710021	0.059
H1C_2	0.830203	0.190139	0.668875	0.059
C2_2	0.5742(6)	0.0985(4)	0.6277(5)	0.060(2)

H2A_2	0.505633	0.084575	0.592818	0.090
H2B_2	0.591361	0.142324	0.671748	0.090
H2C_2	0.577899	0.051437	0.638768	0.090
Cl1_3	0.35915(5)	0.30452(4)	0.80918(3)	0.02436(14)
O1_3	0.15244(14)	0.55465(11)	0.81085(9)	0.0247(4)
S1_3	0.17059(5)	0.37624(4)	0.58282(3)	0.01934(13)
O2_3	0.29980(15)	0.41561(11)	0.88821(9)	0.0244(4)
H2A_3	0.282(2)	0.4455(19)	0.9080(10)	0.037
N1_3	0.21147(17)	0.51665(13)	0.90323(11)	0.0217(5)
O3_3	0.21843(16)	0.45205(11)	0.57868(10)	0.0313(5)
O5_3	0.19900(14)	0.31372(11)	0.55212(9)	0.0254(4)
O6_3	0.06142(14)	0.34924(11)	0.55547(9)	0.0262(4)
C1_3	0.17428(19)	0.57393(15)	0.92441(13)	0.0210(5)
C2_3	0.1677(2)	0.60581(15)	0.98825(14)	0.0237(6)
H2_3	0.192735	0.591230	1.028090	0.028
C3_3	0.1225(2)	0.66016(15)	0.99054(14)	0.0249(6)
H3_3	0.114277	0.681840	1.032525	0.030
C4_3	0.0888(2)	0.68383(16)	0.93312(15)	0.0271(6)
H4_3	0.060325	0.722468	0.937683	0.033
C5_3	0.0956(2)	0.65251(16)	0.86945(15)	0.0284(6)
H5_3	0.072512	0.668114	0.829995	0.034
C6_3	0.1385(2)	0.59700(15)	0.86745(14)	0.0231(5)
C7_3	0.19609(19)	0.50820(15)	0.83738(13)	0.0205(5)
C8_3	0.22037(19)	0.45447(14)	0.79089(13)	0.0201(5)
C9_3	0.19086(19)	0.44384(14)	0.71861(13)	0.0197(5)
H9_3	0.154810	0.472616	0.699127	0.024
C10_3	0.21413(18)	0.39136(14)	0.67534(13)	0.0189(5)
C11_3	0.26956(19)	0.35039(14)	0.70360(13)	0.0197(5)
H11_3	0.288381	0.316585	0.674341	0.024
C12_3	0.29663(19)	0.35966(15)	0.77463(13)	0.0200(5)
C13_3	0.27250(19)	0.41065(15)	0.81996(13)	0.0201(5)
Cl1_4	0.68994(5)	0.29788(4)	0.81023(3)	0.02570(14)
O1_4	0.49009(14)	0.55239(11)	0.81415(9)	0.0234(4)
S1_4	0.51658(5)	0.38174(4)	0.58784(3)	0.01814(13)
O2_4	0.63017(15)	0.40822(11)	0.88927(9)	0.0244(4)
H2A_4	0.614(2)	0.4417(19)	0.9109(10)	0.037
N1_4	0.54627(16)	0.51253(12)	0.90584(11)	0.0206(4)
O3_4	0.57732(14)	0.45818(11)	0.59046(10)	0.0261(4)
O5_4	0.53516(14)	0.31438(11)	0.55348(9)	0.0254(4)
O6_4	0.40923(13)	0.36493(10)	0.55883(9)	0.0226(4)
C1_4	0.51144(19)	0.57117(15)	0.92777(13)	0.0198(5)
C2_4	0.5057(2)	0.60371(15)	0.99160(13)	0.0226(5)
H2_4	0.528890	0.587879	1.030905	0.027
C3_4	0.4645(2)	0.66049(16)	0.99523(14)	0.0250(6)
H3_4	0.458577	0.683530	1.037917	0.030
C4_4	0.4314(2)	0.68479(16)	0.93800(14)	0.0267(6)
H4_4	0.404097	0.724158	0.942905	0.032
C5_4	0.4375(2)	0.65261(16)	0.87367(15)	0.0278(6)

H5_4	0.415899	0.669117	0.834591	0.033
C6_4	0.4768(2)	0.59541(16)	0.87093(13)	0.0233(5)
C7_4	0.53209(19)	0.50466(15)	0.84033(13)	0.0202(5)
C8_4	0.55508(19)	0.45031(15)	0.79367(13)	0.0208(5)
C9_4	0.52856(18)	0.44196(15)	0.72179(13)	0.0194(5)
H9_4	0.494759	0.472342	0.702966	0.023
C10_4	0.55143(19)	0.38972(14)	0.67831(13)	0.0192(5)
C11_4	0.60194(18)	0.34489(15)	0.70511(13)	0.0202(5)
H11_4	0.618125	0.309403	0.675117	0.024
C12_4	0.62776(19)	0.35308(15)	0.77559(13)	0.0205(5)
C13_4	0.60474(19)	0.40458(15)	0.82144(13)	0.0199(5)
Cl1_5	1.02546(5)	0.30347(4)	0.81057(3)	0.02594(14)
O1_5	0.81933(14)	0.55430(11)	0.81799(9)	0.0238(4)
S1_5	0.85151(5)	0.39215(4)	0.59268(3)	0.01919(13)
O2_5	0.96374(14)	0.41150(11)	0.89186(9)	0.0242(4)
H2A_5	0.949(2)	0.4462(19)	0.9148(10)	0.036
N1_5	0.87526(16)	0.51331(12)	0.90903(11)	0.0211(4)
O3_5	0.91503(15)	0.46800(11)	0.59617(10)	0.0297(4)
O5_5	0.86599(15)	0.32386(11)	0.55657(10)	0.0288(4)
O6_5	0.74513(14)	0.37884(12)	0.56450(10)	0.0269(4)
C1_5	0.83847(19)	0.57099(15)	0.93047(13)	0.0210(5)
C2_5	0.8303(2)	0.60207(15)	0.99421(13)	0.0232(5)
H2_5	0.853090	0.585943	1.033376	0.028
C3_5	0.7875(2)	0.65735(15)	0.99721(14)	0.0242(6)
H3_5	0.779849	0.679117	1.039425	0.029
C4_5	0.7549(2)	0.68257(16)	0.94037(14)	0.0265(6)
H4_5	0.726581	0.721284	0.945187	0.032
C5_5	0.7631(2)	0.65204(16)	0.87692(14)	0.0261(6)
H5_5	0.741766	0.668909	0.838054	0.031
C6_5	0.8043(2)	0.59575(15)	0.87416(13)	0.0220(5)
C7_5	0.86187(19)	0.50683(15)	0.84385(13)	0.0203(5)
C8_5	0.88757(19)	0.45444(15)	0.79728(13)	0.0207(5)
C9_5	0.86115(19)	0.44788(15)	0.72610(13)	0.0206(5)
H9_5	0.826066	0.477878	0.707939	0.025
C10_5	0.88577(19)	0.39808(15)	0.68213(13)	0.0194(5)
C11_5	0.93768(19)	0.35366(15)	0.70787(13)	0.0210(5)
H11_5	0.955364	0.319924	0.677577	0.025
C12_5	0.96287(19)	0.35963(15)	0.77819(14)	0.0222(5)
C13_5	0.93816(19)	0.40900(15)	0.82426(13)	0.0204(5)
Cl1_6	1.01316(5)	0.20802(4)	0.18469(3)	0.02546(14)
O1_6	0.64122(13)	-0.04935(10)	0.19149(9)	0.0224(4)
S1_6	1.00876(5)	0.10587(4)	0.40354(3)	0.01887(13)
O2_6	0.80212(14)	0.10041(11)	0.11251(9)	0.0235(4)
H2A_6	0.743(2)	0.0679(19)	0.0925(10)	0.035
N1_6	0.63275(16)	-0.00559(12)	0.10069(11)	0.0201(4)
O3_6	0.96069(14)	0.02582(11)	0.40038(10)	0.0264(4)
O5_6	1.11483(13)	0.12903(11)	0.41711(9)	0.0236(4)
O6_6	0.99608(14)	0.16663(11)	0.45319(9)	0.0267(4)

C1_6	0.53786(19)	-0.06424(15)	0.08231(13)	0.0207(5)
C2_6	0.4464(2)	-0.09507(15)	0.02126(13)	0.0224(5)
H2_6	0.440738	-0.077812	-0.017778	0.027
C3_6	0.3644(2)	-0.15217(15)	0.02066(14)	0.0243(6)
H3_6	0.300842	-0.174303	-0.019684	0.029
C4_6	0.3730(2)	-0.17800(16)	0.07809(14)	0.0254(6)
H4_6	0.315138	-0.217391	0.075441	0.030
C5_6	0.4631(2)	-0.14795(16)	0.13837(14)	0.0262(6)
H5_6	0.469445	-0.165398	0.177339	0.031
C6_6	0.54344(19)	-0.09076(15)	0.13807(14)	0.0224(5)
C7_6	0.68898(19)	0.00016(14)	0.16441(13)	0.0195(5)
C8_6	0.79491(19)	0.05276(14)	0.20747(13)	0.0199(5)
C9_6	0.84543(18)	0.05504(14)	0.27615(13)	0.0194(5)
H9_6	0.810050	0.022516	0.295209	0.023
C10_6	0.94711(19)	0.10459(14)	0.31673(13)	0.0189(5)
C11_6	0.99957(19)	0.15281(15)	0.28886(13)	0.0203(5)
H11_6	1.069176	0.186920	0.316450	0.024
C12_6	0.94868(19)	0.15012(15)	0.22058(13)	0.0207(5)
C13_6	0.84586(19)	0.10043(15)	0.17846(13)	0.0201(5)
Cl1_7	0.66933(5)	0.20733(4)	0.17290(3)	0.02540(14)
O1_7	0.30252(13)	-0.04953(11)	0.18546(9)	0.0240(4)
S1_7	0.67428(5)	0.10761(4)	0.39289(3)	0.01887(13)
O2_7	0.45839(14)	0.09778(11)	0.10221(9)	0.0236(4)
H2A_7	0.396(2)	0.0628(18)	0.0817(10)	0.035
N1_7	0.29123(16)	-0.00826(13)	0.09273(11)	0.0220(5)
O3_7	0.61131(14)	0.03879(11)	0.39907(10)	0.0288(4)
O5_7	0.77116(14)	0.10817(11)	0.39676(10)	0.0250(4)
O6_7	0.69257(15)	0.18470(11)	0.44362(10)	0.0293(4)
C1_7	0.19667(19)	-0.06677(15)	0.07567(14)	0.0215(5)
C2_7	0.1049(2)	-0.09869(16)	0.01549(14)	0.0238(6)
H2_7	0.098128	-0.082514	-0.024215	0.029
C3_7	0.0237(2)	-0.15528(16)	0.01615(14)	0.0258(6)
H3_7	-0.040316	-0.177994	-0.023821	0.031
C4_7	0.0341(2)	-0.17968(16)	0.07454(15)	0.0277(6)
H4_7	-0.023095	-0.219043	0.072703	0.033
C5_7	0.1251(2)	-0.14824(16)	0.13483(15)	0.0282(6)
H5_7	0.132618	-0.164367	0.174607	0.034
C6_7	0.2040(2)	-0.09176(15)	0.13262(13)	0.0229(5)
C7_7	0.34871(19)	-0.00152(15)	0.15653(13)	0.0204(5)
C8_7	0.45445(19)	0.05154(15)	0.19869(13)	0.0211(5)
C9_7	0.50721(19)	0.05498(14)	0.26728(13)	0.0193(5)
H9_7	0.473404	0.022729	0.287271	0.023
C10_7	0.60782(19)	0.10474(15)	0.30618(13)	0.0207(5)
C11_7	0.65927(19)	0.15308(15)	0.27785(13)	0.0204(5)
H11_7	0.728843	0.187504	0.304950	0.024
C12_7	0.60666(19)	0.14968(14)	0.20953(13)	0.0201(5)
C13_7	0.50390(19)	0.09911(15)	0.16842(13)	0.0195(5)
Cl1_8	0.34857(5)	0.21101(4)	0.17852(3)	0.02457(14)

O1_8	-0.01547(13)	-0.04405(11)	0.19546(9)	0.0239(4)
S1_8	0.35564(5)	0.10965(4)	0.39926(3)	0.01900(13)
O2_8	0.13734(14)	0.10263(11)	0.10997(9)	0.0236(4)
H2A_8	0.080(2)	0.0711(19)	0.0920(10)	0.035
N1_8	-0.02904(16)	-0.00383(13)	0.10190(11)	0.0219(5)
O3_8	0.31379(15)	0.02800(11)	0.39425(11)	0.0339(5)
O5_8	0.46218(14)	0.13842(11)	0.41379(10)	0.0253(4)
O6_8	0.33740(15)	0.16468(12)	0.44927(10)	0.0294(4)
C1_8	-0.1230(2)	-0.06218(15)	0.08587(14)	0.0216(5)
C2_8	-0.2157(2)	-0.09526(15)	0.02618(14)	0.0243(6)
H2_8	-0.223805	-0.079956	-0.014080	0.029
C3_8	-0.2954(2)	-0.15127(16)	0.02818(15)	0.0258(6)
H3_8	-0.359843	-0.174444	-0.011475	0.031
C4_8	-0.2842(2)	-0.17488(16)	0.08672(15)	0.0280(6)
H4_8	-0.340973	-0.214030	0.085530	0.034
C5_8	-0.1921(2)	-0.14267(16)	0.14673(15)	0.0274(6)
H5_8	-0.183725	-0.158193	0.186885	0.033
C6_8	-0.1141(2)	-0.08654(15)	0.14353(14)	0.0226(5)
C7_8	0.0298(2)	0.00421(15)	0.16588(14)	0.0218(5)
C8_8	0.1357(2)	0.05705(15)	0.20691(13)	0.0216(5)
C9_8	0.18951(19)	0.06013(15)	0.27509(13)	0.0207(5)
H9_8	0.156049	0.028155	0.295424	0.025
C10_8	0.2909(2)	0.10917(15)	0.31340(13)	0.0207(5)
C11_8	0.34117(19)	0.15717(15)	0.28453(13)	0.0200(5)
H11_8	0.410876	0.191418	0.311101	0.024
C12_8	0.2875(2)	0.15397(15)	0.21632(13)	0.0204(5)
C13_8	0.18420(19)	0.10422(15)	0.17594(13)	0.0197(5)
O1_9	0.23771(14)	0.29012(10)	0.39887(9)	0.0255(4)
S1_9	0.29698(5)	0.37280(4)	0.40238(3)	0.02032(13)
C1_9	0.2805(2)	0.35748(17)	0.31192(14)	0.0293(6)
H1A_9	0.208302	0.331844	0.279627	0.044
H1B_9	0.313568	0.322849	0.298068	0.044
H1C_9	0.310671	0.409367	0.308623	0.044
C2_9	0.2194(2)	0.42598(17)	0.40669(17)	0.0317(6)
H2A_9	0.249949	0.478281	0.404223	0.047
H2B_9	0.212660	0.433994	0.451939	0.047
H2C_9	0.152487	0.395106	0.366307	0.047
O1_10	0.57285(15)	0.30290(11)	0.40305(9)	0.0271(4)
S1_10	0.63683(5)	0.38425(4)	0.40695(3)	0.02201(14)
C1_10	0.6096(2)	0.37102(17)	0.31535(14)	0.0314(7)
H1A_10	0.632151	0.330884	0.295226	0.047
H1B_10	0.644954	0.422215	0.312894	0.047
H1C_10	0.536682	0.352729	0.287750	0.047
C2_10	0.5711(2)	0.44581(17)	0.42256(16)	0.0312(6)
H2A_10	0.606047	0.498645	0.422626	0.047
H2B_10	0.569033	0.452141	0.469125	0.047
H2C_10	0.502107	0.420566	0.384512	0.047
O1_11	0.88748(14)	0.28682(11)	0.39889(10)	0.0274(4)

S1_11	0.95598(5)	0.36706(4)	0.40310(3)	0.02187(14)
C1_11	0.9309(2)	0.35290(17)	0.31188(14)	0.0302(6)
H1A_11	0.951735	0.311254	0.292226	0.045
H1B_11	0.968616	0.403379	0.309432	0.045
H1C_11	0.858432	0.336295	0.283846	0.045
C2_11	0.8939(2)	0.43183(17)	0.41756(17)	0.0327(7)
H2A_11	0.930064	0.483335	0.415965	0.049
H2B_11	0.892746	0.440760	0.464636	0.049
H2C_11	0.824486	0.407110	0.380041	0.049
O1_12	0.30765(15)	0.20488(11)	0.59027(10)	0.0279(4)
S1_12	0.34539(6)	0.13872(4)	0.59314(4)	0.02829(16)
C1_12	0.3580(3)	0.13068(19)	0.67709(15)	0.0401(8)
H1A_12	0.294433	0.123504	0.681361	0.060
H1B_12	0.412323	0.179720	0.716185	0.060
H1C_12	0.374045	0.084389	0.679934	0.060
C2_12	0.2394(2)	0.04852(17)	0.53581(15)	0.0352(7)
H2A_12	0.215830	0.047292	0.486654	0.053
H2B_12	0.185477	0.045031	0.551631	0.053
H2C_12	0.257885	0.002951	0.537168	0.053
O1_13	0.9281(2)	0.18543(14)	0.58053(12)	0.0280(5)
S1_13	0.96063(9)	0.11681(5)	0.58349(4)	0.0221(2)
C1_13	1.0941(2)	0.16207(18)	0.62991(18)	0.0340(8)
H1A_13	1.119112	0.121712	0.639902	0.051
H1B_13	1.114426	0.205414	0.675225	0.051
H1C_13	1.122629	0.184098	0.600280	0.051
C2_13	0.9347(3)	0.09123(19)	0.65266(16)	0.0309(7)
H2A_13	0.861496	0.067416	0.637089	0.046
H2B_13	0.965449	0.139723	0.696172	0.046
H2C_13	0.962801	0.052479	0.662816	0.046
O1_14	0.974(2)	0.2027(12)	0.5862(13)	0.017(4)
S1_14	1.0086(10)	0.1337(6)	0.5896(5)	0.021(2)
C1_14	1.026(3)	0.1289(18)	0.6741(10)	0.027(5)
H1A_14	0.978161	0.145698	0.688996	0.040
H1B_14	1.094868	0.164576	0.709345	0.040
H1C_14	1.012987	0.073610	0.671080	0.040
C2_14	0.8973(19)	0.0451(12)	0.5376(15)	0.027(6)
H2A_14	0.846162	0.047665	0.555239	0.041
H2B_14	0.912075	-0.001513	0.541368	0.041
H2C_14	0.871766	0.039847	0.487274	0.041

U_{eq} is defined as 1/3 of the trace of the orthogonalized U_{ij} tensor.

Table S4. Anisotropic displacement parameters [Å^2] for **4**.
The anisotropic displacement factor exponent takes the form:
$-2\pi^2[h^2(a^*)^2U_{11} + k^2(b^*)^2U_{22} + \ldots + 2hka^*b^*U_{12}]$

Atom	U_{11}	U_{22}	U_{33}	U_{23}	U_{13}	U_{12}
Ca1	0.0240(3)	0.0178(2)	0.0124(2)	0.00321(18)	0.00692(19)	0.0093(2)
Ca2	0.0341(3)	0.0204(3)	0.0130(2)	0.0043(2)	0.0090(2)	0.0150(2)
Ca3	0.0341(3)	0.0202(2)	0.0134(2)	0.0046(2)	0.0093(2)	0.0145(2)
O1_1	0.036(3)	0.020(3)	0.013(3)	0.006(2)	0.006(3)	0.010(2)
S1_1	0.0196(12)	0.0182(14)	0.0218(16)	0.0061(11)	0.0045(11)	0.0067(10)
C1_1	0.105(6)	0.041(4)	0.025(3)	0.011(3)	0.016(3)	0.034(4)
C2_1	0.030(3)	0.036(3)	0.081(5)	0.036(3)	0.003(3)	0.002(3)
O1_2	0.058(5)	0.024(3)	0.023(3)	0.007(3)	−0.004(3)	0.017(3)
S1_2	0.057(3)	0.0272(18)	0.0245(18)	0.0135(13)	0.0057(17)	0.0155(18)
C1_2	0.043(3)	0.030(3)	0.034(3)	0.004(3)	0.005(3)	0.022(3)
C2_2	0.046(4)	0.042(4)	0.099(6)	0.027(4)	0.041(4)	0.013(3)
Cl1_3	0.0301(3)	0.0249(3)	0.0194(3)	0.0074(2)	0.0090(3)	0.0155(3)
O1_3	0.0357(11)	0.0272(10)	0.0167(9)	0.0075(8)	0.0124(8)	0.0191(9)
S1_3	0.0230(3)	0.0208(3)	0.0148(3)	0.0051(2)	0.0093(2)	0.0096(3)
O2_3	0.0315(11)	0.0287(10)	0.0130(8)	0.0053(8)	0.0082(8)	0.0158(9)
N1_3	0.0273(12)	0.0216(11)	0.0150(10)	0.0042(8)	0.0088(9)	0.0107(9)
O3_3	0.0422(12)	0.0246(10)	0.0264(10)	0.0120(8)	0.0164(9)	0.0085(9)
O5_3	0.0279(10)	0.0296(10)	0.0188(9)	0.0042(8)	0.0101(8)	0.0157(8)
O6_3	0.0253(10)	0.0336(11)	0.0175(9)	0.0051(8)	0.0074(8)	0.0149(9)
C1_3	0.0222(13)	0.0216(12)	0.0171(12)	0.0052(10)	0.0078(10)	0.0083(10)
C2_3	0.0259(14)	0.0231(13)	0.0173(12)	0.0033(10)	0.0092(11)	0.0072(11)
C3_3	0.0281(14)	0.0232(13)	0.0191(13)	0.0006(10)	0.0107(11)	0.0100(11)
C4_3	0.0322(15)	0.0261(14)	0.0268(14)	0.0061(11)	0.0156(12)	0.0167(12)
C5_3	0.0395(17)	0.0287(14)	0.0213(13)	0.0084(11)	0.0139(12)	0.0192(13)
C6_3	0.0307(14)	0.0219(13)	0.0179(12)	0.0033(10)	0.0127(11)	0.0131(11)
C7_3	0.0226(13)	0.0191(12)	0.0169(12)	0.0044(10)	0.0070(10)	0.0082(10)
C8_3	0.0235(13)	0.0167(11)	0.0186(12)	0.0044(10)	0.0089(10)	0.0084(10)
C9_3	0.0228(13)	0.0176(12)	0.0170(12)	0.0034(10)	0.0095(10)	0.0076(10)
C10_3	0.0201(12)	0.0191(12)	0.0138(11)	0.0045(9)	0.0065(10)	0.0055(10)
C11_3	0.0226(13)	0.0156(11)	0.0172(12)	0.0022(9)	0.0084(10)	0.0064(10)
C12_3	0.0199(12)	0.0191(12)	0.0204(12)	0.0079(10)	0.0075(10)	0.0079(10)
C13_3	0.0209(13)	0.0184(12)	0.0154(12)	0.0031(10)	0.0059(10)	0.0052(10)
Cl1_4	0.0328(4)	0.0275(3)	0.0207(3)	0.0094(3)	0.0106(3)	0.0182(3)
O1_4	0.0355(11)	0.0243(9)	0.0153(9)	0.0070(7)	0.0118(8)	0.0179(8)
S1_4	0.0202(3)	0.0181(3)	0.0140(3)	0.0039(2)	0.0072(2)	0.0069(2)
O2_4	0.0323(11)	0.0288(10)	0.0165(9)	0.0080(8)	0.0119(8)	0.0166(9)
N1_4	0.0262(11)	0.0208(10)	0.0140(10)	0.0038(8)	0.0082(9)	0.0112(9)
O3_4	0.0298(10)	0.0238(9)	0.0232(10)	0.0087(8)	0.0136(8)	0.0063(8)
O5_4	0.0319(11)	0.0260(10)	0.0183(9)	0.0041(8)	0.0119(8)	0.0142(8)
O6_4	0.0210(9)	0.0248(9)	0.0167(9)	0.0055(7)	0.0054(7)	0.0070(8)
C1_4	0.0215(13)	0.0190(12)	0.0158(12)	0.0036(10)	0.0071(10)	0.0077(10)

C2_4	0.0264(14)	0.0239(13)	0.0149(12)	0.0048(10)	0.0092(10)	0.0083(11)
C3_4	0.0298(14)	0.0230(13)	0.0178(12)	0.0018(10)	0.0109(11)	0.0090(11)
C4_4	0.0335(15)	0.0246(13)	0.0251(14)	0.0060(11)	0.0141(12)	0.0165(12)
C5_4	0.0387(16)	0.0275(14)	0.0217(13)	0.0077(11)	0.0152(12)	0.0177(13)
C6_4	0.0268(14)	0.0250(13)	0.0169(12)	0.0034(10)	0.0098(11)	0.0120(11)
C7_4	0.0229(13)	0.0206(12)	0.0160(12)	0.0063(10)	0.0073(10)	0.0093(10)
C8_4	0.0207(13)	0.0205(12)	0.0182(12)	0.0047(10)	0.0071(10)	0.0080(10)
C9_4	0.0197(12)	0.0197(12)	0.0173(12)	0.0054(10)	0.0074(10)	0.0079(10)
C10_4	0.0197(12)	0.0188(12)	0.0160(12)	0.0048(10)	0.0066(10)	0.0062(10)
C11_4	0.0201(13)	0.0206(12)	0.0171(12)	0.0041(10)	0.0079(10)	0.0070(10)
C12_4	0.0214(13)	0.0208(12)	0.0196(12)	0.0070(10)	0.0071(10)	0.0112(10)
C13_4	0.0215(13)	0.0199(12)	0.0155(12)	0.0048(10)	0.0076(10)	0.0068(10)
Cl1_5	0.0324(4)	0.0260(3)	0.0194(3)	0.0067(3)	0.0079(3)	0.0171(3)
O1_5	0.0336(11)	0.0250(9)	0.0183(9)	0.0076(8)	0.0129(8)	0.0174(8)
S1_5	0.0204(3)	0.0211(3)	0.0148(3)	0.0050(2)	0.0080(2)	0.0076(2)
O2_5	0.0309(10)	0.0280(10)	0.0149(9)	0.0066(8)	0.0091(8)	0.0157(9)
N1_5	0.0254(11)	0.0203(10)	0.0160(10)	0.0036(8)	0.0083(9)	0.0107(9)
O3_5	0.0339(11)	0.0255(10)	0.0265(10)	0.0107(8)	0.0155(9)	0.0041(9)
O5_5	0.0387(12)	0.0285(10)	0.0191(9)	0.0044(8)	0.0133(9)	0.0163(9)
O6_5	0.0236(10)	0.0369(11)	0.0229(10)	0.0124(9)	0.0101(8)	0.0146(9)
C1_5	0.0227(13)	0.0195(12)	0.0168(12)	0.0027(10)	0.0078(10)	0.0074(10)
C2_5	0.0258(14)	0.0224(13)	0.0166(12)	0.0034(10)	0.0073(10)	0.0087(11)
C3_5	0.0250(14)	0.0229(13)	0.0179(12)	−0.0009(10)	0.0101(11)	0.0069(11)
C4_5	0.0292(15)	0.0256(14)	0.0252(14)	0.0056(11)	0.0124(12)	0.0142(12)
C5_5	0.0325(15)	0.0273(14)	0.0182(13)	0.0055(11)	0.0098(11)	0.0155(12)
C6_5	0.0261(14)	0.0228(13)	0.0169(12)	0.0041(10)	0.0098(10)	0.0118(11)
C7_5	0.0247(13)	0.0222(12)	0.0144(11)	0.0064(10)	0.0076(10)	0.0111(11)
C8_5	0.0245(13)	0.0202(12)	0.0160(12)	0.0035(10)	0.0091(10)	0.0095(10)
C9_5	0.0218(13)	0.0193(12)	0.0191(12)	0.0045(10)	0.0088(10)	0.0085(10)
C10_5	0.0195(12)	0.0196(12)	0.0141(11)	0.0036(9)	0.0048(9)	0.0063(10)
C11_5	0.0231(13)	0.0218(12)	0.0156(12)	0.0036(10)	0.0074(10)	0.0093(11)
C12_5	0.0228(13)	0.0228(13)	0.0197(12)	0.0072(10)	0.0064(10)	0.0112(11)
C13_5	0.0211(13)	0.0209(12)	0.0148(11)	0.0052(10)	0.0056(10)	0.0067(10)
Cl1_6	0.0243(3)	0.0246(3)	0.0233(3)	0.0084(3)	0.0094(3)	0.0056(3)
O1_6	0.0196(9)	0.0229(9)	0.0169(9)	0.0049(7)	0.0047(7)	0.0038(7)
S1_6	0.0202(3)	0.0165(3)	0.0156(3)	0.0025(2)	0.0052(2)	0.0074(2)
O2_6	0.0237(10)	0.0265(10)	0.0160(9)	0.0070(8)	0.0063(7)	0.0078(8)
N1_6	0.0193(11)	0.0194(10)	0.0155(10)	0.0016(8)	0.0053(8)	0.0062(9)
O3_6	0.0276(10)	0.0214(9)	0.0226(9)	0.0074(8)	0.0064(8)	0.0054(8)
O5_6	0.0209(9)	0.0244(9)	0.0212(9)	0.0063(8)	0.0062(7)	0.0087(8)
O6_6	0.0301(10)	0.0291(10)	0.0165(9)	0.0011(8)	0.0065(8)	0.0159(9)
C1_6	0.0209(13)	0.0193(12)	0.0181(12)	0.0032(10)	0.0085(10)	0.0059(10)
C2_6	0.0250(13)	0.0216(12)	0.0153(12)	0.0027(10)	0.0063(10)	0.0088(11)
C3_6	0.0212(13)	0.0229(13)	0.0195(13)	−0.0007(10)	0.0048(10)	0.0084(11)
C4_6	0.0220(13)	0.0230(13)	0.0242(13)	0.0039(11)	0.0096(11)	0.0045(11)
C5_6	0.0270(14)	0.0284(14)	0.0215(13)	0.0107(11)	0.0096(11)	0.0089(12)
C6_6	0.0207(13)	0.0211(12)	0.0184(12)	0.0025(10)	0.0056(10)	0.0064(10)
C7_6	0.0210(12)	0.0180(12)	0.0166(12)	0.0028(10)	0.0084(10)	0.0069(10)

C8_6	0.0200(12)	0.0183(12)	0.0169(12)	0.0016(10)	0.0069(10)	0.0070(10)
C9_6	0.0195(12)	0.0176(12)	0.0168(12)	0.0021(9)	0.0061(10)	0.0074(10)
C10_6	0.0210(12)	0.0166(11)	0.0163(12)	0.0017(9)	0.0068(10)	0.0088(10)
C11_6	0.0181(12)	0.0185(12)	0.0207(12)	0.0024(10)	0.0077(10)	0.0074(10)
C12_6	0.0236(13)	0.0176(12)	0.0178(12)	0.0037(10)	0.0086(10)	0.0070(10)
C13_6	0.0205(13)	0.0204(12)	0.0178(12)	0.0041(10)	0.0083(10)	0.0083(10)
Cl1_7	0.0259(3)	0.0250(3)	0.0217(3)	0.0090(3)	0.0096(3)	0.0060(3)
O1_7	0.0204(9)	0.0249(9)	0.0202(9)	0.0067(8)	0.0061(8)	0.0050(8)
S1_7	0.0205(3)	0.0177(3)	0.0162(3)	0.0037(2)	0.0068(2)	0.0083(2)
O2_7	0.0223(10)	0.0254(10)	0.0175(9)	0.0060(8)	0.0065(7)	0.0062(8)
N1_7	0.0219(11)	0.0209(11)	0.0200(11)	0.0045(9)	0.0084(9)	0.0080(9)
O3_7	0.0266(10)	0.0287(10)	0.0230(10)	0.0106(8)	0.0076(8)	0.0032(8)
O5_7	0.0249(10)	0.0327(10)	0.0240(10)	0.0127(8)	0.0120(8)	0.0172(8)
O6_7	0.0342(11)	0.0275(10)	0.0185(9)	0.0002(8)	0.0043(8)	0.0173(9)
C1_7	0.0220(13)	0.0187(12)	0.0200(12)	0.0025(10)	0.0082(10)	0.0080(10)
C2_7	0.0275(14)	0.0242(13)	0.0184(12)	0.0056(10)	0.0086(11)	0.0120(11)
C3_7	0.0239(14)	0.0228(13)	0.0235(13)	0.0016(11)	0.0074(11)	0.0089(11)
C4_7	0.0251(14)	0.0214(13)	0.0297(15)	0.0032(11)	0.0119(12)	0.0054(11)
C5_7	0.0275(15)	0.0263(14)	0.0250(14)	0.0077(11)	0.0100(12)	0.0063(12)
C6_7	0.0232(13)	0.0224(13)	0.0173(12)	0.0029(10)	0.0069(10)	0.0072(11)
C7_7	0.0231(13)	0.0203(12)	0.0159(12)	0.0039(10)	0.0085(10)	0.0086(10)
C8_7	0.0208(13)	0.0200(12)	0.0202(12)	0.0039(10)	0.0082(10)	0.0092(10)
C9_7	0.0230(13)	0.0175(12)	0.0155(11)	0.0037(9)	0.0074(10)	0.0085(10)
C10_7	0.0225(13)	0.0186(12)	0.0180(12)	0.0032(10)	0.0074(10)	0.0087(10)
C11_7	0.0203(12)	0.0186(12)	0.0182(12)	0.0037(10)	0.0063(10)	0.0075(10)
C12_7	0.0243(13)	0.0173(12)	0.0196(12)	0.0045(10)	0.0111(10)	0.0095(10)
C13_7	0.0227(13)	0.0187(12)	0.0160(12)	0.0033(10)	0.0079(10)	0.0103(10)
Cl1_8	0.0269(3)	0.0252(3)	0.0212(3)	0.0098(3)	0.0111(3)	0.0082(3)
O1_8	0.0231(10)	0.0248(9)	0.0208(9)	0.0085(8)	0.0091(8)	0.0061(8)
S1_8	0.0219(3)	0.0191(3)	0.0159(3)	0.0055(2)	0.0078(2)	0.0092(2)
O2_8	0.0237(10)	0.0274(10)	0.0171(9)	0.0081(8)	0.0068(8)	0.0095(8)
N1_8	0.0241(11)	0.0214(11)	0.0176(10)	0.0038(9)	0.0084(9)	0.0092(9)
O3_8	0.0366(12)	0.0233(10)	0.0284(11)	0.0121(9)	0.0035(9)	0.0044(9)
O5_8	0.0228(10)	0.0341(11)	0.0245(10)	0.0118(8)	0.0123(8)	0.0155(8)
O6_8	0.0328(11)	0.0404(12)	0.0179(9)	0.0054(8)	0.0113(8)	0.0229(9)
C1_8	0.0251(13)	0.0194(12)	0.0205(13)	0.0041(10)	0.0114(11)	0.0103(11)
C2_8	0.0259(14)	0.0220(13)	0.0210(13)	0.0040(10)	0.0079(11)	0.0104(11)
C3_8	0.0239(14)	0.0216(13)	0.0238(13)	0.0017(11)	0.0073(11)	0.0075(11)
C4_8	0.0262(14)	0.0223(13)	0.0327(15)	0.0060(12)	0.0149(12)	0.0071(11)
C5_8	0.0300(15)	0.0271(14)	0.0277(14)	0.0118(12)	0.0144(12)	0.0112(12)
C6_8	0.0230(13)	0.0212(12)	0.0192(12)	0.0031(10)	0.0077(10)	0.0083(11)
C7_8	0.0247(13)	0.0205(12)	0.0212(13)	0.0061(10)	0.0120(11)	0.0097(11)
C8_8	0.0273(14)	0.0221(12)	0.0172(12)	0.0058(10)	0.0110(11)	0.0120(11)
C9_8	0.0232(13)	0.0203(12)	0.0193(12)	0.0058(10)	0.0104(10)	0.0097(10)
C10_8	0.0270(14)	0.0179(12)	0.0188(12)	0.0055(10)	0.0113(11)	0.0105(11)
C11_8	0.0207(13)	0.0190(12)	0.0196(12)	0.0048(10)	0.0090(10)	0.0089(10)
C12_8	0.0268(14)	0.0191(12)	0.0168(12)	0.0053(10)	0.0114(10)	0.0101(11)
C13_8	0.0247(13)	0.0203(12)	0.0146(11)	0.0048(10)	0.0082(10)	0.0114(10)

O1_9	0.0314(10)	0.0193(9)	0.0174(9)	0.0064(7)	0.0059(8)	0.0045(8)
S1_9	0.0223(3)	0.0198(3)	0.0154(3)	0.0053(2)	0.0064(2)	0.0068(3)
C1_9	0.0351(16)	0.0262(14)	0.0191(13)	0.0064(11)	0.0117(12)	0.0042(12)
C2_9	0.0311(16)	0.0322(15)	0.0405(17)	0.0180(14)	0.0193(14)	0.0163(13)
O1_10	0.0365(11)	0.0209(9)	0.0176(9)	0.0068(8)	0.0083(8)	0.0073(8)
S1_10	0.0246(3)	0.0241(3)	0.0152(3)	0.0062(2)	0.0068(2)	0.0101(3)
C1_10	0.0380(17)	0.0321(15)	0.0163(13)	0.0077(11)	0.0099(12)	0.0068(13)
C2_10	0.0342(16)	0.0299(15)	0.0370(17)	0.0148(13)	0.0201(14)	0.0154(13)
O1_11	0.0327(11)	0.0249(10)	0.0196(9)	0.0093(8)	0.0074(8)	0.0087(8)
S1_11	0.0221(3)	0.0245(3)	0.0157(3)	0.0064(2)	0.0064(2)	0.0080(3)
C1_11	0.0357(16)	0.0269(14)	0.0204(13)	0.0073(11)	0.0108(12)	0.0052(12)
C2_11	0.0343(16)	0.0311(15)	0.0410(18)	0.0132(14)	0.0234(14)	0.0162(13)
O1_12	0.0418(12)	0.0230(9)	0.0193(9)	0.0080(8)	0.0109(9)	0.0161(9)
S1_12	0.0393(4)	0.0263(3)	0.0198(3)	0.0073(3)	0.0100(3)	0.0181(3)
C1_12	0.062(2)	0.0358(17)	0.0224(15)	0.0100(13)	0.0120(15)	0.0277(16)
C2_12	0.0444(18)	0.0243(14)	0.0264(15)	0.0068(12)	0.0069(13)	0.0117(13)
O1_13	0.0395(14)	0.0326(12)	0.0228(11)	0.0139(10)	0.0163(11)	0.0224(11)
S1_13	0.0262(6)	0.0208(4)	0.0191(4)	0.0071(3)	0.0104(4)	0.0088(4)
C1_13	0.0248(16)	0.0258(16)	0.045(2)	0.0104(14)	0.0140(14)	0.0055(13)
C2_13	0.0391(18)	0.0300(16)	0.0257(16)	0.0130(13)	0.0172(14)	0.0108(14)
O1_14	0.025(5)	0.022(5)	0.019(5)	0.016(4)	0.013(5)	0.016(5)
S1_14	0.024(4)	0.022(3)	0.020(3)	0.013(3)	0.008(3)	0.010(3)
C1_14	0.037(9)	0.024(8)	0.023(7)	0.017(6)	0.011(6)	0.013(7)
C2_14	0.030(10)	0.021(6)	0.027(8)	0.012(8)	0.006(8)	0.010(9)

Table S5. Bond lengths and angles for **4**.

**Atom–Atom**	**Length [Å]**
Ca1–O5_3	2.2695(19)
Ca1–O6_8	2.287(2)
Ca1–O1_9	2.3183(19)
Ca1–O1_12	2.3357(19)
Ca1–O6_4	2.3472(19)
Ca1–O5_6[#1]	2.3525(19)
Ca1–S1_12	3.4353(11)
Ca1–S1_9	3.4523(11)
Ca1–S1_4	3.4919(13)
Ca2–O1_2	2.283(12)
Ca2–O5_4	2.2973(19)
Ca2–O1_1	2.320(11)
Ca2–O1_10	2.3229(19)
Ca2–O5_8	2.341(2)
Ca2–O6_7	2.346(2)
Ca2–O6_5	2.356(2)
Ca2–S1_5	3.4051(13)
Ca2–S1_10	3.4339(11)
Ca2–S1_1	3.468(7)
Ca3–O5_5	2.291(2)
Ca3–O1_11	2.3129(19)
Ca3–O6_6	2.314(2)
Ca3–O1_14	2.32(2)
Ca3–O1_13	2.340(2)
Ca3–O6_3[#2]	2.377(2)
Ca3–O5_7	2.440(2)
Ca3–O6_7	2.862(2)
Ca3–S1_7	3.2237(12)
Ca3–S1_13	3.4514(12)
Ca3–S1_11	3.4669(11)
O1_1–S1_1	1.504(8)
S1_1–C1_1	1.741(8)
S1_1–C2_1	1.772(8)
C1_1–H1A_1	0.9800
C1_1–H1B_1	0.9800
C1_1–H1C_1	0.9800
C2_1–H2A_1	0.9800
C2_1–H2B_1	0.9800
C2_1–H2C_1	0.9800
O1_2–S1_2	1.534(9)
S1_2–C2_2	1.777(9)

S1_2–C1_2	1.788(8)
C1_2–H1A_2	0.9800
C1_2–H1B_2	0.9800
C1_2–H1C_2	0.9800
C2_2–H2A_2	0.9800
C2_2–H2B_2	0.9800
C2_2–H2C_2	0.9800
Cl1_3–C12_3	1.738(3)
O1_3–C7_3	1.364(3)
O1_3–C6_3	1.386(3)
S1_3–O3_3	1.4380(19)
S1_3–O5_3	1.4490(19)
S1_3–O6_3	1.456(2)
S1_3–C10_3	1.776(2)
O2_3–C13_3	1.343(3)
O2_3–H2A_3	0.78(3)
N1_3–C7_3	1.302(3)
N1_3–C1_3	1.403(3)
C1_3–C6_3	1.385(4)
C1_3–C2_3	1.391(3)
C2_3–C3_3	1.390(4)
C2_3–H2_3	0.9500
C3_3–C4_3	1.394(4)
C3_3–H3_3	0.9500
C4_3–C5_3	1.388(4)
C4_3–H4_3	0.9500
C5_3–C6_3	1.383(4)
C5_3–H5_3	0.9500
C7_3–C8_3	1.449(3)
C8_3–C9_3	1.396(3)
C8_3–C13_3	1.417(4)
C9_3–C10_3	1.385(3)
C9_3–H9_3	0.9500
C10_3–C11_3	1.396(3)
C11_3–C12_3	1.380(3)
C11_3–H11_3	0.9500
C12_3–C13_3	1.402(4)
Cl1_4–C12_4	1.737(3)
O1_4–C7_4	1.369(3)
O1_4–C6_4	1.388(3)
S1_4–O3_4	1.4421(19)
S1_4–O5_4	1.4511(19)
S1_4–O6_4	1.4581(19)
S1_4–C10_4	1.775(3)
O2_4–C13_4	1.344(3)
O2_4–H2A_4	0.83(3)
N1_4–C7_4	1.301(3)
N1_4–C1_4	1.402(3)
C1_4–C2_4	1.387(3)
C1_4–C6_4	1.393(4)
C2_4–C3_4	1.387(4)
C2_4–H2_4	0.9500
C3_4–C4_4	1.397(4)
C3_4–H3_4	0.9500
C4_4–C5_4	1.398(4)
C4_4–H4_4	0.9500
C5_4–C6_4	1.375(4)
C5_4–H5_4	0.9500
C7_4–C8_4	1.445(4)
C8_4–C9_4	1.402(3)
C8_4–C13_4	1.414(4)
C9_4–C10_4	1.380(3)
C9_4–H9_4	0.9500
C10_4–C11_4	1.399(3)
C11_4–C12_4	1.375(3)
C11_4–H11_4	0.9500
C12_4–C13_4	1.403(3)
Cl1_5–C12_5	1.737(3)
O1_5–C7_5	1.366(3)
O1_5–C6_5	1.383(3)
S1_5–O3_5	1.4409(19)
S1_5–O5_5	1.441(2)
S1_5–O6_5	1.460(2)
S1_5–C10_5	1.765(3)
O2_5–C13_5	1.345(3)
O2_5–H2A_5	0.85(3)
N1_5–C7_5	1.302(3)
N1_5–C1_5	1.406(3)
C1_5–C6_5	1.389(4)
C1_5–C2_5	1.399(3)
C2_5–C3_5	1.379(4)
C2_5–H2_5	0.9500
C3_5–C4_5	1.400(4)
C3_5–H3_5	0.9500
C4_5–C5_5	1.392(4)
C4_5–H4_5	0.9500
C5_5–C6_5	1.378(4)
C5_5–H5_5	0.9500
C7_5–C8_5	1.451(3)
C8_5–C9_5	1.396(3)
C8_5–C13_5	1.411(4)
C9_5–C10_5	1.377(3)
C9_5–H9_5	0.9500
C10_5–C11_5	1.398(3)
C11_5–C12_5	1.383(3)
C11_5–H11_5	0.9500

C12_5–C13_5	1.401(4)	C4_7–C5_7	1.386(4)
Cl1_6–C12_6	1.736(3)	C4_7–H4_7	0.9500
O1_6–C7_6	1.368(3)	C5_7–C6_7	1.379(4)
O1_6–C6_6	1.386(3)	C5_7–H5_7	0.9500
S1_6–O3_6	1.4424(19)	C7_7–C8_7	1.448(4)
S1_6–O6_6	1.4579(19)	C8_7–C9_7	1.393(3)
S1_6–O5_6	1.4609(19)	C8_7–C13_7	1.411(3)
S1_6–C10_6	1.769(3)	C9_7–C10_7	1.373(3)
O2_6–C13_6	1.342(3)	C9_7–H9_7	0.9500
O2_6–H2A_6	0.82(3)	C10_7–C11_7	1.403(3)
N1_6–C7_6	1.305(3)	C11_7–C12_7	1.388(3)
N1_6–C1_6	1.402(3)	C11_7–H11_7	0.9500
C1_6–C6_6	1.381(4)	C12_7–C13_7	1.404(3)
C1_6–C2_6	1.399(3)	Cl1_8–C12_8	1.731(3)
C2_6–C3_6	1.388(4)	O1_8–C7_8	1.376(3)
C2_6–H2_6	0.9500	O1_8–C6_8	1.385(3)
C3_6–C4_6	1.402(4)	S1_8–O3_8	1.441(2)
C3_6–H3_6	0.9500	S1_8–O6_8	1.4486(19)
C4_6–C5_6	1.381(4)	S1_8–O5_8	1.4585(19)
C4_6–H4_6	0.9500	S1_8–C10_8	1.767(3)
C5_6–C6_6	1.381(4)	O2_8–C13_8	1.340(3)
C5_6–H5_6	0.9500	O2_8–H2A_8	0.80(3)
C7_6–C8_6	1.451(3)	N1_8–C7_8	1.306(3)
C8_6–C9_6	1.395(3)	N1_8–C1_8	1.403(3)
C8_6–C13_6	1.403(3)	C1_8–C6_8	1.390(4)
C9_6–C10_6	1.387(3)	C1_8–C2_8	1.393(4)
C9_6–H9_6	0.9500	C2_8–C3_8	1.381(4)
C10_6–C11_6	1.402(3)	C2_8–H2_8	0.9500
C11_6–C12_6	1.386(3)	C3_8–C4_8	1.396(4)
C11_6–H11_6	0.9500	C3_8–H3_8	0.9500
C12_6–C13_6	1.404(3)	C4_8–C5_8	1.391(4)
Cl1_7–C12_7	1.731(3)	C4_8–H4_8	0.9500
O1_7–C7_7	1.369(3)	C5_8–C6_8	1.379(4)
O1_7–C6_7	1.390(3)	C5_8–H5_8	0.9500
S1_7–O3_7	1.4348(19)	C7_8–C8_8	1.448(4)
S1_7–O5_7	1.4558(19)	C8_8–C9_8	1.392(3)
S1_7–O6_7	1.4626(19)	C8_8–C13_8	1.411(3)
S1_7–C10_7	1.770(3)	C9_8–C10_8	1.379(4)
O2_7–C13_7	1.342(3)	C9_8–H9_8	0.9500
O2_7–H2A_7	0.87(3)	C10_8–C11_8	1.399(3)
N1_7–C7_7	1.306(3)	C11_8–C12_8	1.391(3)
N1_7–C1_7	1.405(3)	C11_8–H11_8	0.9500
C1_7–C6_7	1.385(4)	C12_8–C13_8	1.406(4)
C1_7–C2_7	1.391(4)	O1_9–S1_9	1.5230(19)
C2_7–C3_7	1.388(4)	S1_9–C2_9	1.771(3)
C2_7–H2_7	0.9500	S1_9–C1_9	1.793(3)
C3_7–C4_7	1.403(4)	C1_9–H1A_9	0.9800
C3_7–H3_7	0.9500	C1_9–H1B_9	0.9800

C1_9–H1C_9	0.9800
C2_9–H2A_9	0.9800
C2_9–H2B_9	0.9800
C2_9–H2C_9	0.9800
O1_10–S1_10	1.5216(19)
S1_10–C2_10	1.779(3)
S1_10–C1_10	1.789(3)
C1_10–H1A_10	0.9800
C1_10–H1B_10	0.9800
C1_10–H1C_10	0.9800
C2_10–H2A_10	0.9800
C2_10–H2B_10	0.9800
C2_10–H2C_10	0.9800
O1_11–S1_11	1.5273(19)
S1_11–C2_11	1.777(3)
S1_11–C1_11	1.784(3)
C1_11–H1A_11	0.9800
C1_11–H1B_11	0.9800
C1_11–H1C_11	0.9800
C2_11–H2A_11	0.9800
C2_11–H2B_11	0.9800
C2_11–H2C_11	0.9800
O1_12–S1_12	1.521(2)
S1_12–C2_12	1.758(3)
S1_12–C1_12	1.780(3)
C1_12–H1A_12	0.9800
C1_12–H1B_12	0.9800
C1_12–H1C_12	0.9800
C2_12–H2A_12	0.9800
C2_12–H2B_12	0.9800
C2_12–H2C_12	0.9800
O1_13–S1_13	1.518(2)
S1_13–C1_13	1.772(3)
S1_13–C2_13	1.779(3)
C1_13–H1A_13	0.9800
C1_13–H1B_13	0.9800
C1_13–H1C_13	0.9800
C2_13–H2A_13	0.9800
C2_13–H2B_13	0.9800
C2_13–H2C_13	0.9800
O1_14–S1_14	1.542(14)
S1_14–C1_14	1.759(14)
S1_14–C2_14	1.767(14)
C1_14–H1A_14	0.9800
C1_14–H1B_14	0.9800
C1_14–H1C_14	0.9800
C2_14–H2A_14	0.9800
C2_14–H2B_14	0.9800
C2_14–H2C_14	0.9800

**Atom–Atom–Atom**	**Angle [°]**
O5_3–Ca1–O6_8	168.99(7)
O5_3–Ca1–O1_9	97.31(7)
O6_8–Ca1–O1_9	93.70(7)
O5_3–Ca1–O1_12	84.10(7)
O6_8–Ca1–O1_12	84.93(7)
O1_9–Ca1–O1_12	174.95(7)
O5_3–Ca1–O6_4	81.83(7)
O6_8–Ca1–O6_4	98.92(7)
O1_9–Ca1–O6_4	86.05(7)
O1_12–Ca1–O6_4	89.36(7)
O5_3–Ca1–O5_6$^{\#1}$	97.28(7)
O6_8–Ca1–O5_6$^{\#1}$	83.08(7)
O1_9–Ca1–O5_6$^{\#1}$	88.28(7)
O1_12–Ca1–O5_6$^{\#1}$	96.36(7)
O6_4–Ca1–O5_6$^{\#1}$	174.10(7)
O5_3–Ca1–S1_12	105.05(5)
O6_8–Ca1–S1_12	63.94(5)
O1_9–Ca1–S1_12	157.63(5)
O1_12–Ca1–S1_12	21.37(5)
O6_4–Ca1–S1_12	96.36(5)
O5_6$^{\#1}$–Ca1–S1_12	89.51(5)
O5_3–Ca1–S1_9	91.53(5)
O6_8–Ca1–S1_9	98.79(6)
O1_9–Ca1–S1_9	20.70(4)
O1_12–Ca1–S1_9	154.85(5)
O6_4–Ca1–S1_9	65.49(5)
O5_6$^{\#1}$–Ca1–S1_9	108.77(5)
S1_12–Ca1–S1_9	153.73(3)
O5_3–Ca1–S1_4	97.36(5)
O6_8–Ca1–S1_4	81.88(6)
O1_9–Ca1–S1_4	93.34(5)
O1_12–Ca1–S1_4	81.66(5)
O6_4–Ca1–S1_4	18.15(5)
O5_6$^{\#1}$–Ca1–S1_4	164.94(5)
S1_12–Ca1–S1_4	83.30(3)
S1_9–Ca1–S1_4	74.33(2)
O1_2–Ca2–O5_4	84.3(4)
O5_4–Ca2–O1_1	85.4(3)
O1_2–Ca2–O1_10	179.0(4)
O5_4–Ca2–O1_10	94.67(7)
O1_1–Ca2–O1_10	173.9(3)
O1_2–Ca2–O5_8	94.5(3)
O5_4–Ca2–O5_8	95.91(7)
O1_1–Ca2–O5_8	100.6(3)
O1_10–Ca2–O5_8	85.47(7)

O1_2–Ca2–O6_7	88.9(4)
O5_4–Ca2–O6_7	173.16(7)
O1_1–Ca2–O6_7	87.7(3)
O1_10–Ca2–O6_7	92.14(7)
O5_8–Ca2–O6_7	85.38(7)
O1_2–Ca2–O6_5	93.4(3)
O5_4–Ca2–O6_5	80.11(7)
O1_1–Ca2–O6_5	87.4(3)
O1_10–Ca2–O6_5	86.57(7)
O5_8–Ca2–O6_5	170.79(7)
O6_7–Ca2–O6_5	99.57(7)
O1_2–Ca2–S1_5	84.4(3)
O5_4–Ca2–S1_5	97.69(5)
O1_1–Ca2–S1_5	78.1(3)
O1_10–Ca2–S1_5	95.85(5)
O5_8–Ca2–S1_5	166.18(5)
O6_7–Ca2–S1_5	80.83(5)
O6_5–Ca2–S1_5	20.65(5)
O1_2–Ca2–S1_10	158.8(3)
O5_4–Ca2–S1_10	92.73(5)
O1_1–Ca2–S1_10	152.7(3)
O1_10–Ca2–S1_10	21.21(5)
O5_8–Ca2–S1_10	106.68(5)
O6_7–Ca2–S1_10	93.33(5)
O6_5–Ca2–S1_10	65.48(5)
S1_5–Ca2–S1_10	75.17(3)
O5_4–Ca2–S1_1	100.62(11)
O1_1–Ca2–S1_1	19.7(3)
O1_10–Ca2–S1_1	163.40(12)
O5_8–Ca2–S1_1	86.54(11)
O6_7–Ca2–S1_1	72.72(11)
O6_5–Ca2–S1_1	102.32(12)
S1_5–Ca2–S1_1	88.53(10)
S1_10–Ca2–S1_1	160.22(9)
O5_5–Ca3–O1_11	95.09(7)
O5_5–Ca3–O6_6	160.73(7)
O1_11–Ca3–O6_6	93.75(7)
O5_5–Ca3–O1_14	89.6(5)
O1_11–Ca3–O1_14	162.8(7)
O6_6–Ca3–O1_14	77.3(6)
O5_5–Ca3–O1_13	84.48(8)
O1_11–Ca3–O1_13	178.38(9)
O6_6–Ca3–O1_13	86.21(8)
O5_5–Ca3–O6_3[#2]	76.80(7)
O1_11–Ca3–O6_3[#2]	85.05(7)
O6_6–Ca3–O6_3[#2]	86.96(7)
O1_13–Ca3–O6_3[#2]	93.33(8)
O5_5–Ca3–O5_7	119.33(7)
O1_11–Ca3–O5_7	87.58(7)
O6_6–Ca3–O5_7	78.10(7)
O1_14–Ca3–O5_7	104.6(6)
O1_13–Ca3–O5_7	93.99(8)
O6_3[#2]–Ca3–O5_7	162.87(7)
O5_5–Ca3–O6_7	67.34(7)
O1_11–Ca3–O6_7	87.26(7)
O6_6–Ca3–O6_7	130.27(7)
O1_14–Ca3–O6_7	109.8(7)
O1_13–Ca3–O6_7	94.00(8)
O6_3[#2]–Ca3–O6_7	142.43(6)
O5_7–Ca3–O6_7	52.24(6)
O5_5–Ca3–S1_7	94.14(6)
O1_11–Ca3–S1_7	87.65(5)
O6_6–Ca3–S1_7	103.30(5)
O1_14–Ca3–S1_7	108.6(6)
O1_13–Ca3–S1_7	93.93(7)
O6_3[#2]–Ca3–S1_7	167.79(5)
O5_7–Ca3–S1_7	25.27(4)
O6_7–Ca3–S1_7	26.98(4)
O5_5–Ca3–S1_13	105.45(6)
O1_11–Ca3–S1_13	159.38(6)
O6_6–Ca3–S1_13	66.10(5)
O1_13–Ca3–S1_13	20.97(6)
O6_3[#2]–Ca3–S1_13	97.63(6)
O5_7–Ca3–S1_13	84.04(5)
O6_7–Ca3–S1_13	102.20(5)
S1_7–Ca3–S1_13	92.64(3)
O5_5–Ca3–S1_11	88.23(6)
O1_11–Ca3–S1_11	20.35(5)
O6_6–Ca3–S1_11	94.24(5)
O1_14–Ca3–S1_11	144.1(6)
O1_13–Ca3–S1_11	158.04(7)
O6_3[#2]–Ca3–S1_11	64.80(5)
O5_7–Ca3–S1_11	107.62(5)
O6_7–Ca3–S1_11	102.25(4)
S1_7–Ca3–S1_11	107.27(3)
S1_13–Ca3–S1_11	155.11(3)
S1_1–O1_1–Ca2	128.9(7)
O1_1–S1_1–C1_1	106.8(6)
O1_1–S1_1–C2_1	111.2(5)
C1_1–S1_1–C2_1	99.1(5)
O1_1–S1_1–Ca2	31.4(5)
C1_1–S1_1–Ca2	137.8(4)
C2_1–S1_1–Ca2	96.2(3)
S1_1–C1_1–H1A_1	109.5
S1_1–C1_1–H1B_1	109.5
H1A_1–C1_1–H1B_1	109.5

S1_1–C1_1–H1C_1	109.5
H1A_1–C1_1–H1C_1	109.5
H1B_1–C1_1–H1C_1	109.5
S1_1–C2_1–H2A_1	109.5
S1_1–C2_1–H2B_1	109.5
H2A_1–C2_1–H2B_1	109.5
S1_1–C2_1–H2C_1	109.5
H2A_1–C2_1–H2C_1	109.5
H2B_1–C2_1–H2C_1	109.5
S1_2–O1_2–Ca2	136.2(9)
O1_2–S1_2–C2_2	98.7(7)
O1_2–S1_2–C1_2	104.3(6)
C2_2–S1_2–C1_2	93.4(5)
S1_2–C1_2–H1A_2	109.5
S1_2–C1_2–H1B_2	109.5
H1A_2–C1_2–H1B_2	109.5
S1_2–C1_2–H1C_2	109.5
H1A_2–C1_2–H1C_2	109.5
H1B_2–C1_2–H1C_2	109.5
S1_2–C2_2–H2A_2	109.5
S1_2–C2_2–H2B_2	109.5
H2A_2–C2_2–H2B_2	109.5
S1_2–C2_2–H2C_2	109.5
H2A_2–C2_2–H2C_2	109.5
H2B_2–C2_2–H2C_2	109.5
C7_3–O1_3–C6_3	103.52(19)
O3_3–S1_3–O5_3	114.19(12)
O3_3–S1_3–O6_3	112.84(12)
O5_3–S1_3–O6_3	111.72(11)
O3_3–S1_3–C10_3	107.00(12)
O5_3–S1_3–C10_3	104.87(12)
O6_3–S1_3–C10_3	105.35(11)
C13_3–O2_3–H2A_3	109.5
C7_3–N1_3–C1_3	104.5(2)
S1_3–O5_3–Ca1	162.63(12)
S1_3–O6_3–Ca3[#1]	144.07(12)
C6_3–C1_3–C2_3	120.7(2)
C6_3–C1_3–N1_3	108.1(2)
C2_3–C1_3–N1_3	131.2(2)
C3_3–C2_3–C1_3	116.3(2)
C3_3–C2_3–H2_3	121.8
C1_3–C2_3–H2_3	121.8
C2_3–C3_3–C4_3	122.0(2)
C2_3–C3_3–H3_3	119.0
C4_3–C3_3–H3_3	119.0
C5_3–C4_3–C3_3	121.8(3)
C5_3–C4_3–H4_3	119.1
C3_3–C4_3–H4_3	119.1
C6_3–C5_3–C4_3	115.4(3)
C6_3–C5_3–H5_3	122.3
C4_3–C5_3–H5_3	122.3
C5_3–C6_3–C1_3	123.7(2)
C5_3–C6_3–O1_3	128.0(2)
C1_3–C6_3–O1_3	108.3(2)
N1_3–C7_3–O1_3	115.6(2)
N1_3–C7_3–C8_3	126.1(2)
O1_3–C7_3–C8_3	118.3(2)
C9_3–C8_3–C13_3	120.3(2)
C9_3–C8_3–C7_3	121.3(2)
C13_3–C8_3–C7_3	118.3(2)
C10_3–C9_3–C8_3	120.0(2)
C10_3–C9_3–H9_3	120.0
C8_3–C9_3–H9_3	120.0
C9_3–C10_3–C11_3	120.7(2)
C9_3–C10_3–S1_3	118.64(19)
C11_3–C10_3–S1_3	120.65(19)
C12_3–C11_3–C10_3	119.1(2)
C12_3–C11_3–H11_3	120.5
C10_3–C11_3–H11_3	120.5
C11_3–C12_3–C13_3	122.1(2)
C11_3–C12_3–Cl1_3	119.6(2)
C13_3–C12_3–Cl1_3	118.29(19)
O2_3–C13_3–C12_3	118.8(2)
O2_3–C13_3–C8_3	123.5(2)
C12_3–C13_3–C8_3	117.7(2)
C7_4–O1_4–C6_4	103.62(19)
O3_4–S1_4–O5_4	114.79(12)
O3_4–S1_4–O6_4	112.59(11)
O5_4–S1_4–O6_4	111.58(11)
O3_4–S1_4–C10_4	106.23(11)
O5_4–S1_4–C10_4	105.47(12)
O6_4–S1_4–C10_4	105.29(11)
O3_4–S1_4–Ca1	136.72(8)
O5_4–S1_4–Ca1	82.79(8)
O6_4–S1_4–Ca1	30.10(7)
C10_4–S1_4–Ca1	106.17(8)
C13_4–O2_4–H2A_4	109.5
C7_4–N1_4–C1_4	104.8(2)
S1_4–O5_4–Ca2	155.29(12)
S1_4–O6_4–Ca1	131.75(11)
C2_4–C1_4–C6_4	120.5(2)
C2_4–C1_4–N1_4	131.5(2)

C6_4–C1_4–N1_4	108.1(2)
C3_4–C2_4–C1_4	116.7(2)
C3_4–C2_4–H2_4	121.6
C1_4–C2_4–H2_4	121.6
C2_4–C3_4–C4_4	122.0(2)
C2_4–C3_4–H3_4	119.0
C4_4–C3_4–H3_4	119.0
C3_4–C4_4–C5_4	121.6(3)
C3_4–C4_4–H4_4	119.2
C5_4–C4_4–H4_4	119.2
C6_4–C5_4–C4_4	115.3(3)
C6_4–C5_4–H5_4	122.4
C4_4–C5_4–H5_4	122.4
C5_4–C6_4–O1_4	128.1(2)
C5_4–C6_4–C1_4	123.9(2)
O1_4–C6_4–C1_4	108.0(2)
N1_4–C7_4–O1_4	115.5(2)
N1_4–C7_4–C8_4	126.2(2)
O1_4–C7_4–C8_4	118.3(2)
C9_4–C8_4–C13_4	119.8(2)
C9_4–C8_4–C7_4	121.3(2)
C13_4–C8_4–C7_4	118.9(2)
C10_4–C9_4–C8_4	120.2(2)
C10_4–C9_4–H9_4	119.9
C8_4–C9_4–H9_4	119.9
C9_4–C10_4–C11_4	120.8(2)
C9_4–C10_4–S1_4	118.34(19)
C11_4–C10_4–S1_4	120.81(19)
C12_4–C11_4–C10_4	118.9(2)
C12_4–C11_4–H11_4	120.6
C10_4–C11_4–H11_4	120.6
C11_4–C12_4–C13_4	122.2(2)
C11_4–C12_4–Cl1_4	119.9(2)
C13_4–C12_4–Cl1_4	117.87(19)
O2_4–C13_4–C12_4	119.0(2)
O2_4–C13_4–C8_4	122.9(2)
C12_4–C13_4–C8_4	118.1(2)
C7_5–O1_5–C6_5	103.79(19)
O3_5–S1_5–O5_5	114.70(12)
O3_5–S1_5–O6_5	112.28(12)
O5_5–S1_5–O6_5	111.53(12)
O3_5–S1_5–C10_5	106.43(12)
O5_5–S1_5–C10_5	105.62(12)
O6_5–S1_5–C10_5	105.47(12)
O3_5–S1_5–Ca2	138.52(9)
O5_5–S1_5–Ca2	78.02(9)
O6_5–S1_5–Ca2	34.69(8)
C10_5–S1_5–Ca2	107.39(9)
C13_5–O2_5–H2A_5	109.5
C7_5–N1_5–C1_5	104.1(2)
S1_5–O5_5–Ca3	166.71(13)
S1_5–O6_5–Ca2	124.66(11)
C6_5–C1_5–C2_5	120.3(2)
C6_5–C1_5–N1_5	108.4(2)
C2_5–C1_5–N1_5	131.2(2)
C3_5–C2_5–C1_5	116.5(2)
C3_5–C2_5–H2_5	121.7
C1_5–C2_5–H2_5	121.7
C2_5–C3_5–C4_5	122.4(2)
C2_5–C3_5–H3_5	118.8
C4_5–C3_5–H3_5	118.8
C5_5–C4_5–C3_5	121.3(3)
C5_5–C4_5–H4_5	119.4
C3_5–C4_5–H4_5	119.4
C6_5–C5_5–C4_5	115.6(2)
C6_5–C5_5–H5_5	122.2
C4_5–C5_5–H5_5	122.2
C5_5–C6_5–O1_5	128.3(2)
C5_5–C6_5–C1_5	123.8(2)
O1_5–C6_5–C1_5	107.9(2)
N1_5–C7_5–O1_5	115.8(2)
N1_5–C7_5–C8_5	126.1(2)
O1_5–C7_5–C8_5	118.1(2)
C9_5–C8_5–C13_5	120.2(2)
C9_5–C8_5–C7_5	120.5(2)
C13_5–C8_5–C7_5	119.3(2)
C10_5–C9_5–C8_5	120.2(2)
C10_5–C9_5–H9_5	119.9
C8_5–C9_5–H9_5	119.9
C9_5–C10_5–C11_5	120.8(2)
C9_5–C10_5–S1_5	118.7(2)
C11_5–C10_5–S1_5	120.56(19)
C12_5–C11_5–C10_5	118.9(2)
C12_5–C11_5–H11_5	120.6
C10_5–C11_5–H11_5	120.6
C11_5–C12_5–C13_5	121.9(2)
C11_5–C12_5–Cl1_5	119.5(2)
C13_5–C12_5–Cl1_5	118.60(19)

O2_5–C13_5–C12_5	119.1(2)
O2_5–C13_5–C8_5	122.9(2)
C12_5–C13_5–C8_5	118.0(2)
C7_6–O1_6–C6_6	103.91(19)
O3_6–S1_6–O6_6	113.15(12)
O3_6–S1_6–O5_6	113.74(11)
O6_6–S1_6–O5_6	110.97(11)
O3_6–S1_6–C10_6	106.06(11)
O6_6–S1_6–C10_6	106.90(11)
O5_6–S1_6–C10_6	105.34(11)
C13_6–O2_6–H2A_6	109.5
C7_6–N1_6–C1_6	104.3(2)
S1_6–O5_6–Ca1$^{\#2}$	135.32(11)
S1_6–O6_6–Ca3	153.99(12)
C6_6–C1_6–C2_6	120.2(2)
C6_6–C1_6–N1_6	108.7(2)
C2_6–C1_6–N1_6	131.1(2)
C3_6–C2_6–C1_6	116.6(2)
C3_6–C2_6–H2_6	121.7
C1_6–C2_6–H2_6	121.7
C2_6–C3_6–C4_6	121.7(2)
C2_6–C3_6–H3_6	119.2
C4_6–C3_6–H3_6	119.2
C5_6–C4_6–C3_6	122.0(2)
C5_6–C4_6–H4_6	119.0
C3_6–C4_6–H4_6	119.0
C6_6–C5_6–C4_6	115.3(2)
C6_6–C5_6–H5_6	122.3
C4_6–C5_6–H5_6	122.3
C5_6–C6_6–C1_6	124.2(2)
C5_6–C6_6–O1_6	127.9(2)
C1_6–C6_6–O1_6	107.9(2)
N1_6–C7_6–O1_6	115.2(2)
N1_6–C7_6–C8_6	126.3(2)
O1_6–C7_6–C8_6	118.5(2)
C9_6–C8_6–C13_6	120.7(2)
C9_6–C8_6–C7_6	120.5(2)
C13_6–C8_6–C7_6	118.8(2)
C10_6–C9_6–C8_6	120.3(2)
C10_6–C9_6–H9_6	119.9
C8_6–C9_6–H9_6	119.9
C9_6–C10_6–C11_6	120.0(2)
C9_6–C10_6–S1_6	119.06(19)
C11_6–C10_6–S1_6	120.89(19)
C12_6–C11_6–C10_6	119.2(2)
C12_6–C11_6–H11_6	120.4
C10_6–C11_6–H11_6	120.4
C11_6–C12_6–C13_6	121.8(2)
C11_6–C12_6–Cl1_6	119.7(2)
C13_6–C12_6–Cl1_6	118.42(19)
O2_6–C13_6–C8_6	123.7(2)
O2_6–C13_6–C12_6	118.3(2)
C8_6–C13_6–C12_6	118.0(2)
C7_7–O1_7–C6_7	103.60(19)
O3_7–S1_7–O5_7	115.09(12)
O3_7–S1_7–O6_7	114.38(12)
O5_7–S1_7–O6_7	108.23(12)
O3_7–S1_7–C10_7	106.13(12)
O5_7–S1_7–C10_7	106.07(12)
O6_7–S1_7–C10_7	106.24(12)
O3_7–S1_7–Ca3	133.58(8)
O5_7–S1_7–Ca3	45.67(8)
O6_7–S1_7–Ca3	62.59(8)
C10_7–S1_7–Ca3	119.39(9)
C13_7–O2_7–H2A_7	109.5
C7_7–N1_7–C1_7	104.3(2)
S1_7–O5_7–Ca3	109.07(10)
S1_7–O6_7–Ca2	141.80(12)
S1_7–O6_7–Ca3	90.43(9)
Ca2–O6_7–Ca3	127.32(8)
C6_7–C1_7–C2_7	120.1(2)
C6_7–C1_7–N1_7	108.6(2)
C2_7–C1_7–N1_7	131.3(2)
C3_7–C2_7–C1_7	116.9(2)
C3_7–C2_7–H2_7	121.6
C1_7–C2_7–H2_7	121.6
C2_7–C3_7–C4_7	121.5(3)
C2_7–C3_7–H3_7	119.3
C4_7–C3_7–H3_7	119.3
C5_7–C4_7–C3_7	122.2(3)
C5_7–C4_7–H4_7	118.9
C3_7–C4_7–H4_7	118.9
C6_7–C5_7–C4_7	114.9(3)
C6_7–C5_7–H5_7	122.6
C4_7–C5_7–H5_7	122.6
C5_7–C6_7–C1_7	124.5(2)
C5_7–C6_7–O1_7	127.4(2)
C1_7–C6_7–O1_7	108.0(2)
N1_7–C7_7–O1_7	115.5(2)
N1_7–C7_7–C8_7	126.4(2)
O1_7–C7_7–C8_7	118.1(2)
C9_7–C8_7–C13_7	120.3(2)

C9_7–C8_7–C7_7	121.1(2)
C13_7–C8_7–C7_7	118.6(2)
C10_7–C9_7–C8_7	120.4(2)
C10_7–C9_7–H9_7	119.8
C8_7–C9_7–H9_7	119.8
C9_7–C10_7–C11_7	120.8(2)
C9_7–C10_7–S1_7	119.9(2)
C11_7–C10_7–S1_7	119.29(19)
C12_7–C11_7–C10_7	118.8(2)
C12_7–C11_7–H11_7	120.6
C10_7–C11_7–H11_7	120.6
C11_7–C12_7–C13_7	121.6(2)
C11_7–C12_7–Cl1_7	119.5(2)
C13_7–C12_7–Cl1_7	118.86(19)
O2_7–C13_7–C12_7	118.4(2)
O2_7–C13_7–C8_7	123.6(2)
C12_7–C13_7–C8_7	118.1(2)
C7_8–O1_8–C6_8	103.80(19)
O3_8–S1_8–O6_8	112.88(13)
O3_8–S1_8–O5_8	113.19(12)
O6_8–S1_8–O5_8	111.41(12)
O3_8–S1_8–C10_8	105.98(12)
O6_8–S1_8–C10_8	106.66(12)
O5_8–S1_8–C10_8	106.11(12)
C13_8–O2_8–H2A_8	109.5
C7_8–N1_8–C1_8	104.5(2)
S1_8–O5_8–Ca2	136.69(11)
S1_8–O6_8–Ca1	160.33(12)
C6_8–C1_8–C2_8	119.8(2)
C6_8–C1_8–N1_8	108.5(2)
C2_8–C1_8–N1_8	131.7(2)
C3_8–C2_8–C1_8	117.0(2)
C3_8–C2_8–H2_8	121.5
C1_8–C2_8–H2_8	121.5
C2_8–C3_8–C4_8	122.0(3)
C2_8–C3_8–H3_8	119.0
C4_8–C3_8–H3_8	119.0
C5_8–C4_8–C3_8	121.8(3)
C5_8–C4_8–H4_8	119.1
C3_8–C4_8–H4_8	119.1
C6_8–C5_8–C4_8	115.0(3)
C6_8–C5_8–H5_8	122.5
C4_8–C5_8–H5_8	122.5
C5_8–C6_8–O1_8	127.5(2)
C5_8–C6_8–C1_8	124.4(2)
O1_8–C6_8–C1_8	108.0(2)
N1_8–C7_8–O1_8	115.1(2)
N1_8–C7_8–C8_8	126.7(2)
O1_8–C7_8–C8_8	118.2(2)
C9_8–C8_8–C13_8	120.4(2)
C9_8–C8_8–C7_8	120.7(2)
C13_8–C8_8–C7_8	118.8(2)
C10_8–C9_8–C8_8	120.6(2)
C10_8–C9_8–H9_8	119.7
C8_8–C9_8–H9_8	119.7
C9_8–C10_8–C11_8	120.5(2)
C9_8–C10_8–S1_8	118.59(19)
C11_8–C10_8–S1_8	121.0(2)
C12_8–C11_8–C10_8	119.0(2)
C12_8–C11_8–H11_8	120.5
C10_8–C11_8–H11_8	120.5
C11_8–C12_8–C13_8	121.8(2)
C11_8–C12_8–Cl1_8	119.9(2)
C13_8–C12_8–Cl1_8	118.34(19)
O2_8–C13_8–C12_8	118.8(2)
O2_8–C13_8–C8_8	123.4(2)
C12_8–C13_8–C8_8	117.8(2)
S1_9–O1_9–Ca1	126.74(10)
O1_9–S1_9–C2_9	105.04(13)
O1_9–S1_9–C1_9	104.46(12)
C2_9–S1_9–C1_9	98.14(14)
O1_9–S1_9–Ca1	32.56(7)
C2_9–S1_9–Ca1	109.22(10)
C1_9–S1_9–Ca1	133.05(10)
S1_9–C1_9–H1A_9	109.5
S1_9–C1_9–H1B_9	109.5
H1A_9–C1_9–H1B_9	109.5
S1_9–C1_9–H1C_9	109.5
H1A_9–C1_9–H1C_9	109.5
H1B_9–C1_9–H1C_9	109.5
S1_9–C2_9–H2A_9	109.5
S1_9–C2_9–H2B_9	109.5
H2A_9–C2_9–H2B_9	109.5
S1_9–C2_9–H2C_9	109.5
H2A_9–C2_9–H2C_9	109.5
H2B_9–C2_9–H2C_9	109.5
S1_10–O1_10–Ca2	125.26(11)

O1_10–S1_10–C2_10	104.95(13)
O1_10–S1_10–C1_10	104.31(12)
C2_10–S1_10–C1_10	98.42(15)
O1_10–S1_10–Ca2	33.53(7)
C2_10–S1_10–Ca2	109.99(10)
C1_10–S1_10–Ca2	133.16(10)
S1_10–C1_10–H1A_10	109.5
S1_10–C1_10–H1B_10	109.5
H1A_10–C1_10–H1B_10	109.5
S1_10–C1_10–H1C_10	109.5
H1A_10–C1_10–H1C_10	109.5
H1B_10–C1_10–H1C_10	109.5
S1_10–C2_10–H2A_10	109.5
S1_10–C2_10–H2B_10	109.5
H2A_10–C2_10–H2B_10	109.5
S1_10–C2_10–H2C_10	109.5
H2A_10–C2_10–H2C_10	109.5
H2B_10–C2_10–H2C_10	109.5
S1_11–O1_11–Ca3	127.87(11)
O1_11–S1_11–C2_11	104.72(13)
O1_11–S1_11–C1_11	104.18(12)
C2_11–S1_11–C1_11	98.25(15)
O1_11–S1_11–Ca3	31.78(7)
C2_11–S1_11–Ca3	107.53(10)
C1_11–S1_11–Ca3	132.89(10)
S1_11–C1_11–H1A_11	109.5
S1_11–C1_11–H1B_11	109.5
H1A_11–C1_11–H1B_11	109.5
S1_11–C1_11–H1C_11	109.5
H1A_11–C1_11–H1C_11	109.5
H1B_11–C1_11–H1C_11	109.5
S1_11–C2_11–H2A_11	109.5
S1_11–C2_11–H2B_11	109.5
H2A_11–C2_11–H2B_11	109.5
S1_11–C2_11–H2C_11	109.5
H2A_11–C2_11–H2C_11	109.5
H2B_11–C2_11–H2C_11	109.5
S1_12–O1_12–Ca1	124.59(11)
O1_12–S1_12–C2_12	105.30(14)
O1_12–S1_12–C1_12	105.17(13)
C2_12–S1_12–C1_12	99.07(15)
O1_12–S1_12–Ca1	34.03(7)
C2_12–S1_12–Ca1	93.14(10)
C1_12–S1_12–Ca1	139.12(11)
S1_12–C1_12–H1A_12	109.5
S1_12–C1_12–H1B_12	109.5
H1A_12–C1_12–H1B_12	109.5
S1_12–C1_12–H1C_12	109.5
H1A_12–C1_12–H1C_12	109.5
H1B_12–C1_12–H1C_12	109.5
S1_12–C2_12–H2A_12	109.5
S1_12–C2_12–H2B_12	109.5
H2A_12–C2_12–H2B_12	109.5

S1_12–C2_12–H2C_12	109.5
H2A_12–C2_12–H2C_12	109.5
H2B_12–C2_12–H2C_12	109.5
S1_13–O1_13–Ca3	125.53(13)
O1_13–S1_13–C1_13	105.63(15)
O1_13–S1_13–C2_13	104.49(15)
C1_13–S1_13–C2_13	97.78(16)
O1_13–S1_13–Ca3	33.49(9)
C1_13–S1_13–Ca3	103.67(11)
C2_13–S1_13–Ca3	136.67(12)
S1_13–C1_13–H1A_13	109.5
S1_13–C1_13–H1B_13	109.5
H1A_13–C1_13–H1B_13	109.5
S1_13–C1_13–H1C_13	109.5
H1A_13–C1_13–H1C_13	109.5
H1B_13–C1_13–H1C_13	109.5
S1_13–C2_13–H2A_13	109.5
S1_13–C2_13–H2B_13	109.5
H2A_13–C2_13–H2B_13	109.5
S1_13–C2_13–H2C_13	109.5
H2A_13–C2_13–H2C_13	109.5
H2B_13–C2_13–H2C_13	109.5
S1_14–O1_14–Ca3	129.4(14)
O1_14–S1_14–C1_14	106.1(13)
O1_14–S1_14–C2_14	104.9(13)
C1_14–S1_14–C2_14	97.4(13)
S1_14–C1_14–H1A_14	109.5
S1_14–C1_14–H1B_14	109.5
H1A_14–C1_14–H1B_14	109.5
S1_14–C1_14–H1C_14	109.5
H1A_14–C1_14–H1C_14	109.5
H1B_14–C1_14–H1C_14	109.5
S1_14–C2_14–H2A_14	109.5
S1_14–C2_14–H2B_14	109.5
H2A_14–C2_14–H2B_14	109.5
S1_14–C2_14–H2C_14	109.5
H2A_14–C2_14–H2C_14	109.5
H2B_14–C2_14–H2C_14	109.5

Symmetry transformations used to generate equivalent atoms:
#1: -1+X, +Y, +Z; #2: 1+X, +Y, +Z;

Table S6. Torsion angles for **4**.

**Atom–Atom–Atom–Atom**	**Torsion Angle [°]**
Ca2–O1_1–S1_1–C1_1	−172.8(7)
Ca2–O1_1–S1_1–C2_1	−65.6(10)
Ca2–O1_2–S1_2–C2_2	−128.9(10)
Ca2–O1_2–S1_2–C1_2	135.4(9)
O3_3–S1_3–O5_3–Ca1	−9.9(4)
O6_3–S1_3–O5_3–Ca1	119.7(4)
C10_3–S1_3–O5_3–Ca1	−126.7(4)
O3_3–S1_3–O6_3–Ca3$^{\#1}$	140.90(18)
O5_3–S1_3–O6_3–Ca3$^{\#1}$	10.6(2)
C10_3–S1_3–O6_3–Ca3$^{\#1}$	−102.70(19)
C7_3–N1_3–C1_3–C6_3	−0.4(3)
C7_3–N1_3–C1_3–C2_3	178.2(3)
C6_3–C1_3–C2_3–C3_3	0.9(4)
N1_3–C1_3–C2_3–C3_3	−177.6(3)

C1_3–C2_3–C3_3–C4_3	−2.3(4)
C2_3–C3_3–C4_3–C5_3	2.0(4)
C3_3–C4_3–C5_3–C6_3	−0.4(4)
C4_3–C5_3–C6_3–C1_3	−1.0(4)
C4_3–C5_3–C6_3–O1_3	178.1(3)
C2_3–C1_3–C6_3–C5_3	0.7(4)
N1_3–C1_3–C6_3–C5_3	179.5(3)
C2_3–C1_3–C6_3–O1_3	−178.5(2)
N1_3–C1_3–C6_3–O1_3	0.2(3)
C7_3–O1_3–C6_3–C5_3	−179.2(3)
C7_3–O1_3–C6_3–C1_3	0.0(3)
C1_3–N1_3–C7_3–O1_3	0.5(3)
C1_3–N1_3–C7_3–C8_3	−179.1(2)
C6_3–O1_3–C7_3–N1_3	−0.4(3)
C6_3–O1_3–C7_3–C8_3	179.3(2)
N1_3–C7_3–C8_3–C9_3	173.1(3)
O1_3–C7_3–C8_3–C9_3	−6.6(4)
N1_3–C7_3–C8_3–C13_3	−5.0(4)
O1_3–C7_3–C8_3–C13_3	175.4(2)
C13_3–C8_3–C9_3–C10_3	−1.2(4)
C7_3–C8_3–C9_3–C10_3	−179.2(2)
C8_3–C9_3–C10_3–C11_3	−1.9(4)
C8_3–C9_3–C10_3–S1_3	177.33(19)
O3_3–S1_3–C10_3–C9_3	63.4(2)
O5_3–S1_3–C10_3–C9_3	−174.93(19)
O6_3–S1_3–C10_3–C9_3	−56.9(2)
O3_3–S1_3–C10_3–C11_3	−117.4(2)
O5_3–S1_3–C10_3–C11_3	4.3(2)
O6_3–S1_3–C10_3–C11_3	122.3(2)
C9_3–C10_3–C11_3–C12_3	3.3(4)
S1_3–C10_3–C11_3–C12_3	−175.89(19)

C10_3–C11_3–C12_3–C13_3	−1.7(4)
C10_3–C11_3–C12_3–Cl1_3	176.23(19)
C11_3–C12_3–C13_3–O2_3	178.9(2)
Cl1_3–C12_3–C13_3–O2_3	0.9(3)
C11_3–C12_3–C13_3–C8_3	−1.2(4)
Cl1_3–C12_3–C13_3–C8_3	−179.19(19)
C9_3–C8_3–C13_3–O2_3	−177.4(2)
C7_3–C8_3–C13_3–O2_3	0.7(4)
C9_3–C8_3–C13_3–C12_3	2.7(4)
C7_3–C8_3–C13_3–C12_3	−179.2(2)
O3_4–S1_4–O5_4–Ca2	−2.6(3)
O6_4–S1_4–O5_4–Ca2	127.0(3)
C10_4–S1_4–O5_4–Ca2	−119.2(3)
Ca1–S1_4–O5_4–Ca2	136.0(3)
O3_4–S1_4–O6_4–Ca1	148.72(13)
O5_4–S1_4–O6_4–Ca1	17.96(17)
C10_4–S1_4–O6_4–Ca1	−95.97(15)
C7_4–N1_4–C1_4–C2_4	178.8(3)
C7_4–N1_4–C1_4–C6_4	0.1(3)
C6_4–C1_4–C2_4–C3_4	0.0(4)
N1_4–C1_4–C2_4–C3_4	−178.6(3)
C1_4–C2_4–C3_4–C4_4	−0.8(4)
C2_4–C3_4–C4_4–C5_4	0.5(4)
C3_4–C4_4–C5_4–C6_4	0.7(4)
C4_4–C5_4–C6_4–O1_4	178.7(3)
C4_4–C5_4–C6_4–C1_4	−1.6(4)
C7_4–O1_4–C6_4–C5_4	179.7(3)
C7_4–O1_4–C6_4–C1_4	0.0(3)
C2_4–C1_4–C6_4–C5_4	1.3(4)
N1_4–C1_4–C6_4–C5_4	−179.8(3)
C2_4–C1_4–C6_4–O1_4	−178.9(2)
N1_4–C1_4–C6_4–O1_4	−0.1(3)
C1_4–N1_4–C7_4–O1_4	−0.2(3)
C1_4–N1_4–C7_4–C8_4	−179.3(2)
C6_4–O1_4–C7_4–N1_4	0.1(3)

C6_4–O1_4–C7_4–C8_4	179.3(2)
N1_4–C7_4–C8_4–C9_4	174.4(2)
O1_4–C7_4–C8_4–C9_4	−4.6(4)
N1_4–C7_4–C8_4–C13_4	−5.2(4)
O1_4–C7_4–C8_4–C13_4	175.7(2)
C13_4–C8_4–C9_4–C10_4	−0.5(4)
C7_4–C8_4–C9_4–C10_4	179.9(2)
C8_4–C9_4–C10_4–C11_4	−0.5(4)
C8_4–C9_4–C10_4–S1_4	−179.77(19)
O3_4–S1_4–C10_4–C9_4	66.7(2)
O5_4–S1_4–C10_4–C9_4	−171.08(19)
O6_4–S1_4–C10_4–C9_4	−53.0(2)
Ca1–S1_4–C10_4–C9_4	−84.3(2)
O3_4–S1_4–C10_4–C11_4	−112.6(2)
O5_4–S1_4–C10_4–C11_4	9.7(2)
O6_4–S1_4–C10_4–C11_4	127.8(2)
Ca1–S1_4–C10_4–C11_4	96.5(2)
C9_4–C10_4–C11_4–C12_4	0.6(4)
S1_4–C10_4–C11_4–C12_4	179.83(19)
C10_4–C11_4–C12_4–C13_4	0.3(4)
C10_4–C11_4–C12_4–Cl1_4	179.69(19)
C11_4–C12_4–C13_4–O2_4	178.5(2)
Cl1_4–C12_4–C13_4–O2_4	−0.9(3)
C11_4–C12_4–C13_4–C8_4	−1.3(4)
Cl1_4–C12_4–C13_4–C8_4	179.32(19)
C9_4–C8_4–C13_4–O2_4	−178.4(2)
C7_4–C8_4–C13_4–O2_4	1.2(4)
C9_4–C8_4–C13_4–C12_4	1.3(4)
C7_4–C8_4–C13_4–C12_4	−179.0(2)
O3_5–S1_5–O5_5–Ca3	−19.5(6)
O6_5–S1_5–O5_5–Ca3	109.6(6)
C10_5–S1_5–O5_5–Ca3	−136.4(5)
Ca2–S1_5–O5_5–Ca3	118.7(6)
O3_5–S1_5–O6_5–Ca2	146.16(12)
O5_5–S1_5–O6_5–Ca2	15.83(16)
C10_5–S1_5–O6_5–Ca2	−98.35(14)
C7_5–N1_5–C1_5–C6_5	0.0(3)
C7_5–N1_5–C1_5–C2_5	178.4(3)
C6_5–C1_5–C2_5–C3_5	−0.1(4)
N1_5–C1_5–C2_5–C3_5	−178.2(3)
C1_5–C2_5–C3_5–C4_5	−0.9(4)
C2_5–C3_5–C4_5–C5_5	0.7(4)
C3_5–C4_5–C5_5–C6_5	0.5(4)
C4_5–C5_5–C6_5–O1_5	178.4(3)
C4_5–C5_5–C6_5–C1_5	−1.5(4)
C7_5–O1_5–C6_5–C5_5	−179.9(3)
C7_5–O1_5–C6_5–C1_5	0.0(3)
C2_5–C1_5–C6_5–C5_5	1.4(4)
N1_5–C1_5–C6_5–C5_5	179.9(2)
C2_5–C1_5–C6_5–O1_5	−178.5(2)
N1_5–C1_5–C6_5–O1_5	0.0(3)
C1_5–N1_5–C7_5–O1_5	−0.1(3)
C1_5–N1_5–C7_5–C8_5	−179.9(2)
C6_5–O1_5–C7_5–N1_5	0.1(3)
C6_5–O1_5–C7_5–C8_5	179.9(2)
N1_5–C7_5–C8_5–C9_5	174.3(3)
O1_5–C7_5–C8_5–C9_5	−5.6(4)
N1_5–C7_5–C8_5–C13_5	−4.6(4)
O1_5–C7_5–C8_5–C13_5	175.5(2)
C13_5–C8_5–C9_5–C10_5	−1.1(4)
C7_5–C8_5–C9_5–C10_5	180.0(2)
C8_5–C9_5–C10_5–C11_5	−0.2(4)

C8_5–C9_5–C10_5–S1_5	−179.62(19)
O3_5–S1_5–C10_5–C9_5	70.2(2)
O5_5–S1_5–C10_5–C9_5	−167.4(2)
O6_5–S1_5–C10_5–C9_5	−49.2(2)
Ca2–S1_5–C10_5–C9_5	−85.4(2)
O3_5–S1_5–C10_5–C11_5	−109.2(2)
O5_5–S1_5–C10_5–C11_5	13.2(2)
O6_5–S1_5–C10_5–C11_5	131.4(2)
Ca2–S1_5–C10_5–C11_5	95.2(2)
C9_5–C10_5–C11_5–C12_5	0.9(4)
S1_5–C10_5–C11_5–C12_5	−179.7(2)
C10_5–C11_5–C12_5–C13_5	−0.2(4)
C10_5–C11_5–C12_5–Cl1_5	178.89(19)
C11_5–C12_5–C13_5–O2_5	179.2(2)
Cl1_5–C12_5–C13_5–O2_5	0.1(3)
C11_5–C12_5–C13_5–C8_5	−1.1(4)
Cl1_5–C12_5–C13_5–C8_5	179.83(19)
C9_5–C8_5–C13_5–O2_5	−178.6(2)
C7_5–C8_5–C13_5–O2_5	0.3(4)
C9_5–C8_5–C13_5–C12_5	1.7(4)
C7_5–C8_5–C13_5–C12_5	−179.3(2)
O3_6–S1_6–O5_6–Ca1[#2]	−135.23(14)
O6_6–S1_6–O5_6–Ca1[#2]	−6.30(19)
C10_6–S1_6–O5_6–Ca1[#2]	109.04(16)
O3_6–S1_6–O6_6–Ca3	−75.6(3)
O5_6–S1_6–O6_6–Ca3	155.2(3)
C10_6–S1_6–O6_6–Ca3	40.8(3)
C7_6–N1_6–C1_6–C6_6	0.0(3)
C7_6–N1_6–C1_6–C2_6	178.5(3)
C6_6–C1_6–C2_6–C3_6	0.1(4)
N1_6–C1_6–C2_6–C3_6	−178.3(3)
C1_6–C2_6–C3_6–C4_6	−0.4(4)
C2_6–C3_6–C4_6–C5_6	0.3(4)
C3_6–C4_6–C5_6–C6_6	0.2(4)
C4_6–C5_6–C6_6–C1_6	−0.6(4)
C4_6–C5_6–C6_6–O1_6	178.5(3)
C2_6–C1_6–C6_6–C5_6	0.5(4)
N1_6–C1_6–C6_6–C5_6	179.2(3)
C2_6–C1_6–C6_6–O1_6	−178.8(2)
N1_6–C1_6–C6_6–O1_6	−0.1(3)
C7_6–O1_6–C6_6–C5_6	−179.1(3)
C7_6–O1_6–C6_6–C1_6	0.1(3)
C1_6–N1_6–C7_6–O1_6	0.0(3)
C1_6–N1_6–C7_6–C8_6	179.7(2)
C6_6–O1_6–C7_6–N1_6	−0.1(3)
C6_6–O1_6–C7_6–C8_6	−179.7(2)
N1_6–C7_6–C8_6–C9_6	177.7(2)
O1_6–C7_6–C8_6–C9_6	−2.7(4)
N1_6–C7_6–C8_6–C13_6	−3.1(4)
O1_6–C7_6–C8_6–C13_6	176.5(2)
C13_6–C8_6–C9_6–C10_6	−0.1(4)
C7_6–C8_6–C9_6–C10_6	179.1(2)
C8_6–C9_6–C10_6–C11_6	0.1(4)
C8_6–C9_6–C10_6–S1_6	−179.93(19)
O3_6–S1_6–C10_6–C9_6	32.4(2)
O6_6–S1_6–C10_6–C9_6	−88.6(2)
O5_6–S1_6–C10_6–C9_6	153.3(2)
O3_6–S1_6–C10_6–C11_6	−147.6(2)
O6_6–S1_6–C10_6–C11_6	91.4(2)

O5_6–S1_6–C10_6–C11_6	−26.7(2)
C9_6–C10_6–C11_6–C12_6	−0.1(4)
S1_6–C10_6–C11_6–C12_6	179.95(19)
C10_6–C11_6–C12_6–C13_6	0.0(4)
C10_6–C11_6–C12_6–Cl1_6	−178.78(19)
C9_6–C8_6–C13_6–O2_6	179.2(2)
C7_6–C8_6–C13_6–O2_6	0.0(4)
C9_6–C8_6–C13_6–C12_6	0.0(4)
C7_6–C8_6–C13_6–C12_6	−179.1(2)
C11_6–C12_6–C13_6–O2_6	−179.2(2)
Cl1_6–C12_6–C13_6–O2_6	−0.4(3)
C11_6–C12_6–C13_6–C8_6	0.0(4)
Cl1_6–C12_6–C13_6–C8_6	178.84(19)
O3_7–S1_7–O5_7–Ca3	−127.36(11)
O6_7–S1_7–O5_7–Ca3	1.98(13)
C10_7–S1_7–O5_7–Ca3	115.64(11)
O3_7–S1_7–O6_7–Ca2	−43.7(2)
O5_7–S1_7–O6_7–Ca2	−173.48(16)
C10_7–S1_7–O6_7–Ca2	73.0(2)
Ca3–S1_7–O6_7–Ca2	−171.9(2)
O3_7–S1_7–O6_7–Ca3	128.14(10)
O5_7–S1_7–O6_7–Ca3	−1.60(10)
C10_7–S1_7–O6_7–Ca3	−115.14(10)
C7_7–N1_7–C1_7–C6_7	0.3(3)
C7_7–N1_7–C1_7–C2_7	178.9(3)
C6_7–C1_7–C2_7–C3_7	0.1(4)
N1_7–C1_7–C2_7–C3_7	−178.4(3)
C1_7–C2_7–C3_7–C4_7	−0.6(4)
C2_7–C3_7–C4_7–C5_7	0.7(4)
C3_7–C4_7–C5_7–C6_7	−0.2(4)
C4_7–C5_7–C6_7–C1_7	−0.4(4)
C4_7–C5_7–C6_7–O1_7	178.8(3)
C2_7–C1_7–C6_7–C5_7	0.4(4)
N1_7–C1_7–C6_7–C5_7	179.2(3)
C2_7–C1_7–C6_7–O1_7	−178.9(2)
N1_7–C1_7–C6_7–O1_7	−0.1(3)
C7_7–O1_7–C6_7–C5_7	−179.5(3)
C7_7–O1_7–C6_7–C1_7	−0.2(3)
C1_7–N1_7–C7_7–O1_7	−0.4(3)
C1_7–N1_7–C7_7–C8_7	−179.9(2)
C6_7–O1_7–C7_7–N1_7	0.4(3)
C6_7–O1_7–C7_7–C8_7	179.9(2)
N1_7–C7_7–C8_7–C9_7	178.1(3)
O1_7–C7_7–C8_7–C9_7	−1.3(4)
N1_7–C7_7–C8_7–C13_7	−2.9(4)
O1_7–C7_7–C8_7–C13_7	177.6(2)
C13_7–C8_7–C9_7–C10_7	0.1(4)
C7_7–C8_7–C9_7–C10_7	179.1(2)
C8_7–C9_7–C10_7–C11_7	0.2(4)
C8_7–C9_7–C10_7–S1_7	−179.25(19)
O3_7–S1_7–C10_7–C9_7	14.2(2)
O5_7–S1_7–C10_7–C9_7	137.1(2)
O6_7–S1_7–C10_7–C9_7	−107.9(2)
Ca3–S1_7–C10_7–C9_7	−175.20(17)
O3_7–S1_7–C10_7–C11_7	−165.2(2)
O5_7–S1_7–C10_7–C11_7	−42.4(2)
O6_7–S1_7–C10_7–C11_7	72.7(2)
Ca3–S1_7–C10_7–C11_7	5.4(2)
C9_7–C10_7–C11_7–C12_7	−0.1(4)
S1_7–C10_7–C11_7–C12_7	179.30(19)
C10_7–C11_7–C12_7–C13_7	−0.2(4)
C10_7–C11_7–C12_7–Cl1_7	−178.73(19)

C11_7–C12_7–C13_7–O2_7	−178.7(2)
Cl1_7–C12_7–C13_7–O2_7	−0.1(3)
C11_7–C12_7–C13_7–C8_7	0.4(4)
Cl1_7–C12_7–C13_7–C8_7	178.96(19)
C9_7–C8_7–C13_7–O2_7	178.7(2)
C7_7–C8_7–C13_7–O2_7	−0.3(4)
C9_7–C8_7–C13_7–C12_7	−0.3(4)
C7_7–C8_7–C13_7–C12_7	−179.3(2)
O3_8–S1_8–O5_8–Ca2	−131.79(15)
O6_8–S1_8–O5_8–Ca2	−3.3(2)
C10_8–S1_8–O5_8–Ca2	112.39(16)
O3_8–S1_8–O6_8–Ca1	−98.2(4)
O5_8–S1_8–O6_8–Ca1	133.1(4)
C10_8–S1_8–O6_8–Ca1	17.8(4)
C7_8–N1_8–C1_8–C6_8	−0.2(3)
C7_8–N1_8–C1_8–C2_8	178.9(3)
C6_8–C1_8–C2_8–C3_8	0.4(4)
N1_8–C1_8–C2_8–C3_8	−178.5(3)
C1_8–C2_8–C3_8–C4_8	−0.7(4)
C2_8–C3_8–C4_8–C5_8	0.6(4)
C3_8–C4_8–C5_8–C6_8	−0.2(4)
C4_8–C5_8–C6_8–O1_8	178.9(3)
C4_8–C5_8–C6_8–C1_8	−0.1(4)
C7_8–O1_8–C6_8–C5_8	−178.9(3)
C7_8–O1_8–C6_8–C1_8	0.2(3)
C2_8–C1_8–C6_8–C5_8	0.0(4)
N1_8–C1_8–C6_8–C5_8	179.1(3)
C2_8–C1_8–C6_8–O1_8	−179.2(2)
N1_8–C1_8–C6_8–O1_8	0.0(3)
C1_8–N1_8–C7_8–O1_8	0.3(3)
C1_8–N1_8–C7_8–C8_8	179.4(2)
C6_8–O1_8–C7_8–N1_8	−0.3(3)
C6_8–O1_8–C7_8–C8_8	−179.5(2)
N1_8–C7_8–C8_8–C9_8	179.5(3)
O1_8–C7_8–C8_8–C9_8	−1.5(4)
N1_8–C7_8–C8_8–C13_8	−1.9(4)
O1_8–C7_8–C8_8–C13_8	177.2(2)
C13_8–C8_8–C9_8–C10_8	−0.1(4)
C7_8–C8_8–C9_8–C10_8	178.6(2)
C8_8–C9_8–C10_8–C11_8	0.5(4)
C8_8–C9_8–C10_8–S1_8	−179.2(2)
O3_8–S1_8–C10_8–C9_8	37.1(2)
O6_8–S1_8–C10_8–C9_8	−83.4(2)
O5_8–S1_8–C10_8–C9_8	157.7(2)
O3_8–S1_8–C10_8–C11_8	−142.7(2)
O6_8–S1_8–C10_8–C11_8	96.8(2)
O5_8–S1_8–C10_8–C11_8	−22.1(2)
C9_8–C10_8–C11_8–C12_8	−0.7(4)
S1_8–C10_8–C11_8–C12_8	179.08(19)
C10_8–C11_8–C12_8–C13_8	0.4(4)
C10_8–C11_8–C12_8–Cl1_8	−178.50(19)
C11_8–C12_8–C13_8–O2_8	−178.6(2)
Cl1_8–C12_8–C13_8–O2_8	0.3(3)
C11_8–C12_8–C13_8–C8_8	0.1(4)
Cl1_8–C12_8–C13_8–C8_8	178.98(19)
C9_8–C8_8–C13_8–O2_8	178.4(2)
C7_8–C8_8–C13_8–O2_8	−0.2(4)
C9_8–C8_8–C13_8–C12_8	−0.3(4)
C7_8–C8_8–C13_8–C12_8	−178.9(2)
Ca1–O1_9–S1_9–C2_9	−102.27(16)
Ca1–O1_9–S1_9–C1_9	154.98(15)

Ca2–O1_10–S1_10–C2_10	−103.74(16)
Ca2–O1_10–S1_10–C1_10	153.28(15)
Ca3–O1_11–S1_11–C2_11	−99.63(17)
Ca3–O1_11–S1_11–C1_11	157.70(15)
Ca1–O1_12–S1_12–C2_12	−72.32(17)
Ca1–O1_12–S1_12–C1_12	−176.42(15)
Ca3–O1_13–S1_13–C1_13	91.24(19)
Ca3–O1_13–S1_13–C2_13	−166.19(17)
Ca3–O1_14–S1_14–C1_14	−166.6(18)
Ca3–O1_14–S1_14–C2_14	−64(2)

Symmetry transformations used to generate equivalent atoms:
#1: -1+X, +Y, +Z;   #2: 1+X, +Y, +Z;

Crystallographic data of 5:

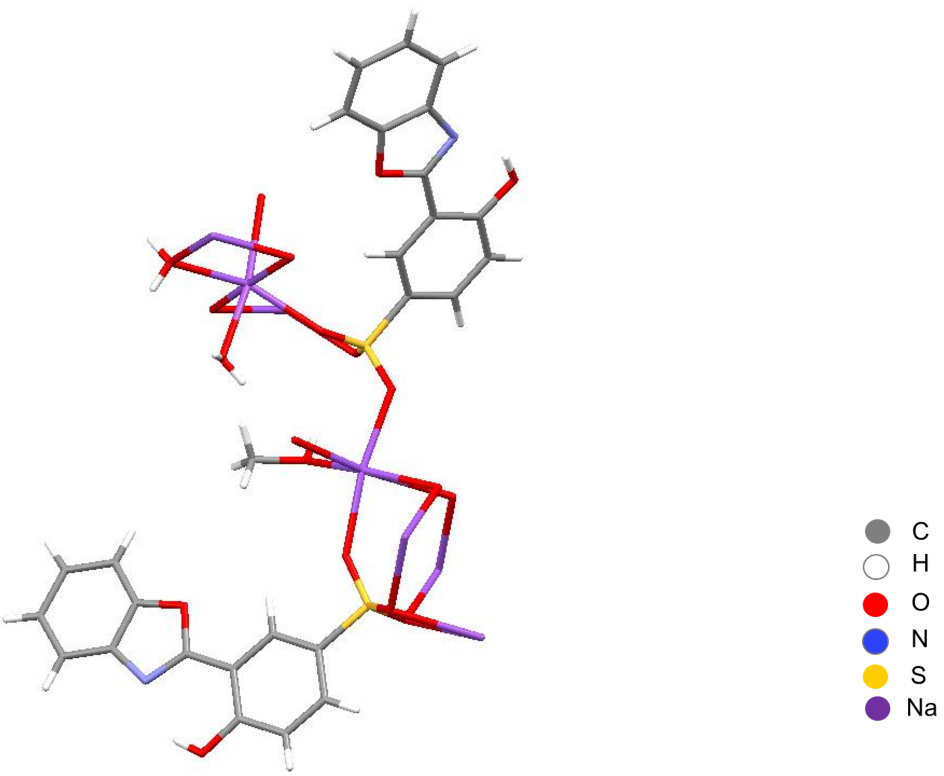

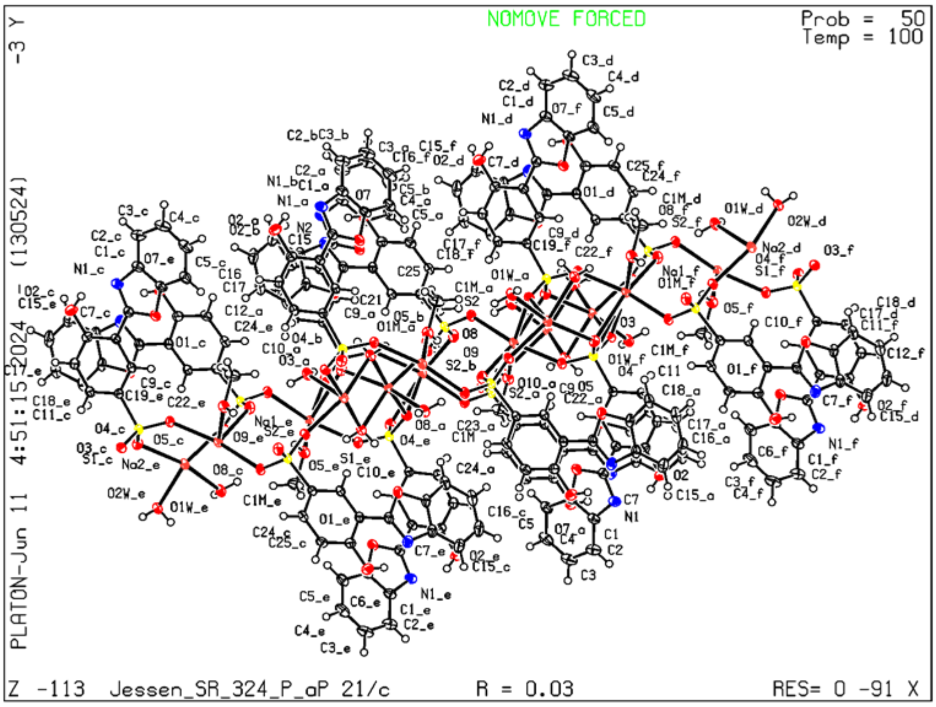

Figure S10: Crystal Structure of **5.**

Crystals were obtained at room temperature from methanol by slow solvent evaporation. A yellow, plate-shaped crystal of **5** was mounted on a MiTeGen micromount with perfluoroether oil. Data were collected from a shock-cooled single crystal at 100(2) K on a Bruker D8 VENTURE dual wavelength Mo/Cu three-circle diffractometer with a microfocus sealed X-ray tube using a mirror optics as monochromator and a Bruker PHOTON III detector. The diffractometer was equipped with an Oxford Cryostream 800 low temperature device and used MoK_α radiation (λ = 0.71073 Å). All data were integrated with SAINT and a multi-scan absorption correction using SADABS was applied.[2,3] The structure was solved by direct methods using SHELXT and refined by full-matrix least-squares methods against F^2 by SHELXL-2019/2.[4,5] All non-hydrogen atoms were refined with anisotropic displacement parameters. All C-bound hydrogen atoms were refined with isotropic displacement parameters. Some of their coordinates were refined freely and some on calculated positions using a riding model with their U_{iso} values constrained to 1.5 times the U_{eq} of their pivot atoms for terminal sp^3 carbon atoms and 1.2 times for all other carbon atoms. Crystallographic data for the structures reported here have been deposited with the Cambridge Crystallographic Data Centre.[8] CCDC 2361756 contain the supplementary crystallographic data for this paper. These data can be obtained free of charge from The Cambridge Crystallographic Data Centre via www.ccdc.cam.ac.uk/ structures. This report and the CIF file were generated using FinalCif.[9]

Table S7. Crystal data and structure refinement for **5**.

CCDC number	2361756
Empirical formula	$C_{27}H_{24}N_2Na_2O_{13}S_2$
Formula weight	694.58
Temperature [K]	100(2)
Crystal system	monoclinic
Space group (number)	$P2_1/c$ (14)
a [Å]	19.360(6)
b [Å]	8.134(2)
c [Å]	18.684(5)
α [°]	90
β [°]	92.357(6)
γ [°]	90
Volume [Å^3]	2940.0(14)
Z	4
ρ_{calc} [gcm^{-3}]	1.569
μ [mm^{-1}]	0.283
F(000)	1432
Crystal size [mm^3]	0.488×0.209×0.020
Crystal colour	yellow

Crystal shape	plate
Radiation	MoK_α (λ=0.71073 Å)
2θ range [°]	4.21 to 56.73 (0.75 Å)
Index ranges	−25 ≤ h ≤ 25<br>−10 ≤ k ≤ 10<br>−24 ≤ l ≤ 24
Reflections collected	196988
Independent reflections	7341<br>R_{int} = 0.0616<br>R_{sigma} = 0.0168
Completeness to<br>θ = 25.242°	100.0 %
Data / Restraints / Parameters	7341/6/436
Absorption correction<br>T_{min}/T_{max} (method)	0.6845/0.7457<br>(multi-scan)
Goodness-of-fit on F^2	1.041
Final R indexes<br>[I≥2σ(I)]	R_1 = 0.0340<br>wR_2 = 0.0819
Final R indexes<br>[all data]	R_1 = 0.0430<br>wR_2 = 0.0873
Largest peak/hole [eÅ^{-3}]	0.59/−0.44

Table S8. Atomic coordinates and U_{eq} [Å^2] for 5.

Atom	*x*	*y*	*z*	U_{eq}
S1	0.40148(2)	0.52705(5)	1.01549(2)	0.01459(8)
S2	0.58184(2)	0.22841(5)	0.72796(2)	0.01473(8)
Na1	0.49158(3)	0.41691(8)	0.85860(3)	0.01728(13)
Na2	0.49633(3)	0.32409(7)	0.55355(3)	0.01647(13)
O1	0.18180(6)	0.27968(14)	0.88152(6)	0.0204(2)
O2	0.10038(6)	0.54725(17)	1.05296(7)	0.0269(3)
H2A	0.0796(7)	0.503(3)	1.0200(12)	0.040
O3	0.42896(6)	0.41254(14)	1.06966(6)	0.0181(2)
O4	0.42800(6)	0.69322(14)	1.02931(6)	0.0193(2)
O5	0.41053(6)	0.47216(15)	0.94254(6)	0.0208(2)
O6	0.73338(6)	0.46431(14)	0.52105(6)	0.0206(2)
O7	0.87547(6)	0.20123(17)	0.65570(7)	0.0264(3)
H7A	0.8836(3)	0.262(3)	0.6193(13)	0.040
O8	0.58287(6)	0.32525(14)	0.79344(6)	0.0198(2)
O9	0.55909(6)	0.05973(14)	0.74009(6)	0.0214(2)
O10	0.54398(6)	0.30744(16)	0.66886(6)	0.0238(3)
N1	0.08419(7)	0.36269(18)	0.93328(8)	0.0218(3)
N2	0.84542(7)	0.39889(18)	0.54439(8)	0.0234(3)
C1	0.06612(9)	0.2609(2)	0.87454(9)	0.0227(3)
C2	0.00225(10)	0.2079(2)	0.84670(10)	0.0290(4)
H2	-0.039723	0.239745	0.867366	0.035
C3	0.00297(10)	0.1056(2)	0.78699(11)	0.0338(4)

H3	-0.039734	0.068422	0.765981	0.041
C4	0.06426(11)	0.0562(2)	0.75711(10)	0.0315(4)
H4	0.062264	-0.014408	0.716565	0.038
C5	0.12844(10)	0.1075(2)	0.78513(9)	0.0262(4)
H5	0.170631	0.073596	0.765489	0.031
C6	0.12634(9)	0.2109(2)	0.84338(9)	0.0221(3)
C7	0.15130(8)	0.3688(2)	0.93410(8)	0.0192(3)
C8	0.19639(8)	0.45680(19)	0.98440(8)	0.0177(3)
C9	0.26805(8)	0.45651(19)	0.97645(8)	0.0167(3)
H9	0.286860	0.401757	0.936779	0.020
C10	0.31142(8)	0.53504(18)	1.02572(8)	0.0154(3)
C11	0.28405(8)	0.6150(2)	1.08477(8)	0.0192(3)
H11	0.314063	0.666861	1.119363	0.023
C12	0.21340(8)	0.6182(2)	1.09255(9)	0.0215(3)
H12	0.194960	0.673931	1.132162	0.026
C13	0.16885(8)	0.5400(2)	1.04255(9)	0.0200(3)
C14	0.84039(9)	0.4964(2)	0.48216(9)	0.0237(3)
C15	0.89092(10)	0.5547(2)	0.43748(10)	0.0305(4)
H15	0.938515	0.530484	0.446249	0.037
C16	0.86817(11)	0.6497(2)	0.37970(10)	0.0334(4)
H16	0.901092	0.691644	0.348099	0.040
C17	0.79845(11)	0.6855(2)	0.36651(9)	0.0311(4)
H17	0.785349	0.750693	0.326016	0.037
C18	0.74728(10)	0.6290(2)	0.41063(9)	0.0260(4)
H18	0.699633	0.652938	0.402033	0.031
C19	0.77171(9)	0.5349(2)	0.46819(8)	0.0223(3)
C20	0.78206(8)	0.3852(2)	0.56391(8)	0.0200(3)
C21	0.75948(8)	0.2975(2)	0.62628(8)	0.0184(3)
C22	0.68975(8)	0.29844(19)	0.64361(8)	0.0172(3)
H22	0.656729	0.355438	0.613912	0.021
C23	0.66896(8)	0.21663(19)	0.70382(8)	0.0162(3)
C24	0.71698(8)	0.1298(2)	0.74699(8)	0.0193(3)
H24	0.702353	0.073258	0.788192	0.023
C25	0.78568(9)	0.1261(2)	0.72988(9)	0.0220(3)
H25	0.818075	0.066301	0.759231	0.026
C26	0.80792(8)	0.2100(2)	0.66954(9)	0.0203(3)
C1M	0.36629(10)	0.2006(2)	0.77082(11)	0.0329(4)
H1MA	0.380775	0.222274	0.722067	0.049
H1MB	0.340032	0.294877	0.787842	0.049
H1MC	0.337150	0.102132	0.770809	0.049
O1M	0.42609(6)	0.17529(15)	0.81722(6)	0.0223(2)
H1	0.4478(12)	0.099(3)	0.8036(12)	0.033
O1W	0.41637(6)	0.50423(15)	0.60806(6)	0.0216(2)
H1W1	0.4225(11)	0.510(3)	0.6517(9)	0.032
H2W1	0.4147(11)	0.599(2)	0.5926(11)	0.032
O2W	0.42706(6)	0.44028(14)	0.45766(6)	0.0194(2)
H1W2	0.3881(9)	0.448(3)	0.4736(11)	0.029
H2W2	0.4196(11)	0.394(3)	0.4184(10)	0.029

U_{eq} is defined as 1/3 of the trace of the orthogonalized U_{ij} tensor.

Table S9. Anisotropic displacement parameters [Å^2] for **5**.
The anisotropic displacement factor exponent takes the form:
$-2\pi^2[\, h^2(a^*)^2U_{11} + k^2(b^*)^2U_{22} + \ldots + 2hka^*b^*U_{12}\,]$

**Atom**	U_{11}	U_{22}	U_{33}	U_{23}	U_{13}	U_{12}
S1	0.01713(17)	0.01375(17)	0.01292(16)	−0.00046(13)	0.00108(12)	−0.00063(13)
S2	0.01926(17)	0.01467(17)	0.01020(15)	0.00020(13)	−0.00002(12)	−0.00046(13)
Na1	0.0203(3)	0.0192(3)	0.0123(3)	−0.0016(2)	0.0008(2)	0.0004(2)
Na2	0.0228(3)	0.0135(3)	0.0130(3)	0.0003(2)	−0.0002(2)	0.0005(2)
O1	0.0228(6)	0.0218(6)	0.0165(5)	−0.0002(4)	−0.0019(4)	−0.0042(5)
O2	0.0189(6)	0.0306(7)	0.0315(7)	−0.0045(5)	0.0025(5)	0.0004(5)
O3	0.0207(5)	0.0164(5)	0.0170(5)	0.0017(4)	−0.0009(4)	0.0017(4)
O4	0.0223(5)	0.0143(5)	0.0216(5)	−0.0003(4)	0.0038(4)	−0.0028(4)
O5	0.0215(5)	0.0266(6)	0.0145(5)	−0.0042(4)	0.0032(4)	−0.0003(5)
O6	0.0251(6)	0.0217(6)	0.0153(5)	0.0011(4)	0.0038(4)	−0.0035(5)
O7	0.0201(6)	0.0321(7)	0.0271(6)	−0.0031(5)	0.0018(5)	0.0027(5)
O8	0.0227(5)	0.0208(6)	0.0159(5)	−0.0055(4)	0.0022(4)	−0.0007(4)
O9	0.0280(6)	0.0164(6)	0.0200(5)	−0.0006(4)	0.0042(4)	−0.0044(5)
O10	0.0223(6)	0.0345(7)	0.0144(5)	0.0079(5)	−0.0006(4)	0.0038(5)
N1	0.0213(7)	0.0198(7)	0.0239(7)	0.0043(6)	−0.0039(5)	−0.0016(5)
N2	0.0258(7)	0.0235(7)	0.0212(7)	−0.0056(6)	0.0059(5)	−0.0041(6)
C1	0.0254(8)	0.0185(8)	0.0237(8)	0.0062(6)	−0.0060(6)	−0.0029(6)
C2	0.0256(9)	0.0239(9)	0.0368(10)	0.0063(7)	−0.0088(7)	−0.0034(7)
C3	0.0334(10)	0.0285(10)	0.0380(10)	0.0048(8)	−0.0162(8)	−0.0073(8)
C4	0.0426(11)	0.0250(9)	0.0258(9)	0.0007(7)	−0.0115(8)	−0.0074(8)
C5	0.0336(9)	0.0245(9)	0.0201(8)	0.0042(7)	−0.0035(7)	−0.0054(7)
C6	0.0254(8)	0.0204(8)	0.0200(7)	0.0060(6)	−0.0062(6)	−0.0064(6)
C7	0.0222(7)	0.0170(7)	0.0182(7)	0.0039(6)	−0.0007(6)	−0.0005(6)
C8	0.0203(7)	0.0164(7)	0.0162(7)	0.0029(6)	−0.0013(5)	−0.0003(6)
C9	0.0206(7)	0.0154(7)	0.0140(7)	0.0016(5)	0.0002(5)	0.0004(6)
C10	0.0178(7)	0.0134(7)	0.0149(7)	0.0025(5)	0.0008(5)	0.0005(5)
C11	0.0228(8)	0.0178(7)	0.0169(7)	−0.0019(6)	0.0004(6)	−0.0008(6)
C12	0.0238(8)	0.0203(8)	0.0205(7)	−0.0031(6)	0.0031(6)	0.0016(6)
C13	0.0188(7)	0.0181(7)	0.0232(8)	0.0013(6)	0.0019(6)	0.0014(6)
C14	0.0306(9)	0.0217(8)	0.0193(8)	−0.0063(6)	0.0062(6)	−0.0074(7)
C15	0.0325(9)	0.0334(10)	0.0264(9)	−0.0085(8)	0.0105(7)	−0.0099(8)
C16	0.0458(11)	0.0319(10)	0.0235(9)	−0.0056(7)	0.0148(8)	−0.0170(9)
C17	0.0502(12)	0.0255(9)	0.0182(8)	−0.0026(7)	0.0072(7)	−0.0125(8)
C18	0.0387(10)	0.0228(8)	0.0168(7)	−0.0023(6)	0.0031(7)	−0.0074(7)
C19	0.0314(9)	0.0191(8)	0.0168(7)	−0.0041(6)	0.0077(6)	−0.0086(7)
C20	0.0245(8)	0.0189(8)	0.0165(7)	−0.0049(6)	0.0017(6)	−0.0030(6)
C21	0.0222(7)	0.0170(7)	0.0160(7)	−0.0033(6)	0.0021(6)	−0.0011(6)
C22	0.0216(7)	0.0147(7)	0.0150(7)	−0.0012(5)	−0.0003(5)	0.0000(6)
C23	0.0200(7)	0.0146(7)	0.0139(7)	−0.0022(5)	−0.0002(5)	0.0002(6)
C24	0.0260(8)	0.0171(7)	0.0146(7)	0.0005(6)	−0.0016(6)	0.0015(6)
C25	0.0240(8)	0.0205(8)	0.0210(8)	−0.0021(6)	−0.0044(6)	0.0043(6)

C26	0.0198(7)	0.0199(8)	0.0213(7)	−0.0057(6)	−0.0003(6)	0.0015(6)
C1M	0.0332(10)	0.0251(9)	0.0394(10)	0.0072(8)	−0.0104(8)	−0.0013(7)
O1M	0.0270(6)	0.0190(6)	0.0207(6)	0.0001(5)	0.0000(5)	0.0042(5)
O1W	0.0302(6)	0.0182(6)	0.0164(5)	−0.0014(5)	−0.0001(5)	0.0020(5)
O2W	0.0240(6)	0.0194(6)	0.0147(5)	−0.0013(4)	−0.0007(4)	0.0000(5)

Table S10. Bond lengths and angles for **5**.

**Atom–Atom**	**Length [Å]**
S1–O5	1.4517(12)
S1–O3	1.4597(11)
S1–O4	1.4651(12)
S1–C10	1.7631(16)
S1–Na1$^{\#1}$	3.1029(10)
S2–O10	1.4503(12)
S2–O8	1.4545(12)
S2–O9	1.4615(12)
S2–C23	1.7665(17)
S2–Na1$^{\#2}$	3.2966(9)
Na1–O5	2.3079(13)
Na1–O8	2.3107(13)
Na1–O9$^{\#3}$	2.3586(14)
Na1–O3$^{\#1}$	2.4327(13)
Na1–O1M	2.4469(15)
Na1–O4$^{\#1}$	2.7101(14)
Na1–Na2$^{\#3}$	3.6997(11)
Na1–Na2$^{\#4}$	4.1343(13)
Na2–O10	2.3124(14)
Na2–O3$^{\#5}$	2.3514(13)
Na2–O2W	2.3884(13)
Na2–O1W	2.3897(14)
Na2–O4$^{\#2}$	2.4214(13)
Na2–O2W$^{\#6}$	2.4377(14)
Na2–Na2$^{\#6}$	3.4983(14)
O1–C7	1.374(2)
O1–C6	1.3825(19)
O2–C13	1.349(2)
O2–H2A	0.81(3)
O6–C20	1.371(2)
O6–C19	1.3842(19)
O7–C26	1.345(2)
O7–H7A	0.86(3)
N1–C7	1.300(2)
N1–C1	1.407(2)
N2–C20	1.299(2)
N2–C14	1.407(2)
C1–C6	1.385(3)
C1–C2	1.390(2)
C2–C3	1.392(3)
C2–H2	0.9500
C3–C4	1.391(3)
C3–H3	0.9500
C4–C5	1.392(3)
C4–H4	0.9500
C5–C6	1.377(2)
C5–H5	0.9500
C7–C8	1.446(2)
C8–C9	1.401(2)
C8–C13	1.404(2)
C9–C10	1.377(2)
C9–H9	0.9500
C10–C11	1.403(2)
C11–C12	1.382(2)
C11–H11	0.9500
C12–C13	1.398(2)
C12–H12	0.9500
C14–C19	1.380(3)
C14–C15	1.395(2)
C15–C16	1.385(3)
C15–H15	0.9500
C16–C17	1.393(3)
C16–H16	0.9500
C17–C18	1.392(3)
C17–H17	0.9500
C18–C19	1.387(2)
C18–H18	0.9500
C20–C21	1.449(2)
C21–C22	1.401(2)
C21–C26	1.406(2)
C22–C23	1.381(2)
C22–H22	0.9500
C23–C24	1.397(2)
C24–C25	1.381(2)
C24–H24	0.9500
C25–C26	1.401(2)
C25–H25	0.9500
C1M–O1M	1.433(2)
C1M–H1MA	0.9800

C1M–H1MB	0.9800
C1M–H1MC	0.9800
O1M–H1	0.80(2)
O1W–H1W1	0.821(17)
O1W–H2W1	0.826(18)
O2W–H1W2	0.824(17)
O2W–H2W2	0.832(17)

**Atom–Atom–Atom**	**Angle [°]**
O5–S1–O3	113.58(7)
O5–S1–O4	113.22(7)
O3–S1–O4	110.57(7)
O5–S1–C10	105.74(7)
O3–S1–C10	106.40(7)
O4–S1–C10	106.73(7)
O5–S1–Na1$^{\#1}$	130.88(5)
O3–S1–Na1$^{\#1}$	49.85(5)
O4–S1–Na1$^{\#1}$	60.84(5)
C10–S1–Na1$^{\#1}$	122.99(5)
O10–S2–O8	112.92(7)
O10–S2–O9	112.84(7)
O8–S2–O9	111.83(7)
O10–S2–C23	106.71(7)
O8–S2–C23	105.30(7)
O9–S2–C23	106.59(7)
O10–S2–Na1$^{\#2}$	76.87(6)
O8–S2–Na1$^{\#2}$	145.06(5)
O9–S2–Na1$^{\#2}$	39.46(5)
C23–S2–Na1$^{\#2}$	103.29(5)
O5–Na1–O8	167.71(5)
O5–Na1–O9$^{\#3}$	99.37(5)
O8–Na1–O9$^{\#3}$	92.84(5)
O5–Na1–O3$^{\#1}$	86.95(5)
O8–Na1–O3$^{\#1}$	89.67(5)
O9$^{\#3}$–Na1–O3$^{\#1}$	112.53(5)
O5–Na1–O1M	90.76(5)
O8–Na1–O1M	88.39(5)
O9$^{\#3}$–Na1–O1M	87.43(5)
O3$^{\#1}$–Na1–O1M	160.02(5)
O5–Na1–O4$^{\#1}$	85.66(5)
O8–Na1–O4$^{\#1}$	82.70(5)
O9$^{\#3}$–Na1–O4$^{\#1}$	166.96(5)
O3$^{\#1}$–Na1–O4$^{\#1}$	55.43(4)
O1M–Na1–O4$^{\#1}$	104.62(4)
O5–Na1–S1$^{\#1}$	84.68(4)
O8–Na1–S1$^{\#1}$	86.89(4)
O9$^{\#3}$–Na1–S1$^{\#1}$	139.77(4)
O3$^{\#1}$–Na1–S1$^{\#1}$	27.30(3)
O1M–Na1–S1$^{\#1}$	132.72(4)
O4$^{\#1}$–Na1–S1$^{\#1}$	28.17(3)
O5–Na1–S2$^{\#3}$	83.82(4)
O8–Na1–S2$^{\#3}$	108.25(4)
O9$^{\#3}$–Na1–S2$^{\#3}$	23.19(3)
O3$^{\#1}$–Na1–S2$^{\#3}$	94.71(4)
O1M–Na1–S2$^{\#3}$	104.78(4)
O4$^{\#1}$–Na1–S2$^{\#3}$	148.83(3)
S1$^{\#1}$–Na1–S2$^{\#3}$	121.32(2)
O5–Na1–Na2$^{\#3}$	63.68(4)
O8–Na1–Na2$^{\#3}$	119.13(4)
O9$^{\#3}$–Na1–Na2$^{\#3}$	85.56(4)
O3$^{\#1}$–Na1–Na2$^{\#3}$	38.56(3)
O1M–Na1–Na2$^{\#3}$	151.86(4)
O4$^{\#1}$–Na1–Na2$^{\#3}$	85.94(3)
S1$^{\#1}$–Na1–Na2$^{\#3}$	60.268(17)
S2$^{\#3}$–Na1–Na2$^{\#3}$	63.11(2)
O5–Na1–Na2$^{\#4}$	58.98(4)
O8–Na1–Na2$^{\#4}$	108.75(4)
O9$^{\#3}$–Na1–Na2$^{\#4}$	156.32(4)
O3$^{\#1}$–Na1–Na2$^{\#4}$	78.13(3)
O1M–Na1–Na2$^{\#4}$	83.68(4)
O4$^{\#1}$–Na1–Na2$^{\#4}$	34.02(3)
S1$^{\#1}$–Na1–Na2$^{\#4}$	53.966(19)
S2$^{\#3}$–Na1–Na2$^{\#4}$	142.21(2)
Na2$^{\#3}$–Na1–Na2$^{\#4}$	92.00(3)
O10–Na2–O3$^{\#5}$	92.00(5)
O10–Na2–O2W	155.82(5)
O3$^{\#5}$–Na2–O2W	97.04(5)
O10–Na2–O1W	83.08(5)
O3$^{\#5}$–Na2–O1W	94.30(5)
O2W–Na2–O1W	73.94(5)
O10–Na2–O4$^{\#2}$	109.96(5)
O3$^{\#5}$–Na2–O4$^{\#2}$	94.32(5)
O2W–Na2–O4$^{\#2}$	91.73(5)
O1W–Na2–O4$^{\#2}$	164.08(5)
O10–Na2–O2W$^{\#6}$	84.57(5)
O3$^{\#5}$–Na2–O2W$^{\#6}$	175.74(5)
O2W–Na2–O2W$^{\#6}$	87.09(5)
O1W–Na2–O2W$^{\#6}$	87.80(5)
O4$^{\#2}$–Na2–O2W$^{\#6}$	84.52(5)
O10–Na2–Na2$^{\#6}$	123.84(5)
O3$^{\#5}$–Na2–Na2$^{\#6}$	141.14(4)
O2W–Na2–Na2$^{\#6}$	44.10(3)
O1W–Na2–Na2$^{\#6}$	77.55(4)
O4$^{\#2}$–Na2–Na2$^{\#6}$	87.36(4)
O2W$^{\#6}$–Na2–Na2$^{\#6}$	42.99(3)
O10–Na2–Na1$^{\#2}$	61.23(4)

O3$^{\#5}$–Na2–Na1$^{\#2}$	40.15(3)
O2W–Na2–Na1$^{\#2}$	135.27(4)
O1W–Na2–Na1$^{\#2}$	112.97(4)
O4$^{\#2}$–Na2–Na1$^{\#2}$	82.02(4)
O2W$^{\#6}$–Na2–Na1$^{\#2}$	135.59(4)
Na2$^{\#6}$–Na2–Na1$^{\#2}$	169.37(3)
O10–Na2–Na1$^{\#5}$	141.87(4)
O3$^{\#5}$–Na2–Na1$^{\#5}$	74.47(3)
O2W–Na2–Na1$^{\#5}$	62.30(4)
O1W–Na2–Na1$^{\#5}$	132.44(4)
O4$^{\#2}$–Na2–Na1$^{\#5}$	38.77(3)
O2W$^{\#6}$–Na2–Na1$^{\#5}$	106.78(4)
Na2$^{\#6}$–Na2–Na1$^{\#5}$	83.33(3)
Na1$^{\#2}$–Na2–Na1$^{\#5}$	88.00(3)
C7–O1–C6	103.57(13)
C13–O2–H2A	109.5
S1–O3–Na2$^{\#4}$	128.45(7)
S1–O3–Na1$^{\#1}$	102.85(6)
Na2$^{\#4}$–O3–Na1$^{\#1}$	101.29(5)
S1–O4–Na2$^{\#3}$	120.66(7)
S1–O4–Na1$^{\#1}$	90.99(6)
Na2$^{\#3}$–O4–Na1$^{\#1}$	107.22(5)
S1–O5–Na1	144.06(7)
C20–O6–C19	103.62(13)
C26–O7–H7A	109.5
S2–O8–Na1	129.31(7)
S2–O9–Na1$^{\#2}$	117.35(7)
S2–O10–Na2	154.84(8)
C7–N1–C1	104.35(14)
C20–N2–C14	104.24(14)
C6–C1–C2	120.16(17)
C6–C1–N1	108.30(14)
C2–C1–N1	131.53(17)
C1–C2–C3	116.56(18)
C1–C2–H2	121.7
C3–C2–H2	121.7
C4–C3–C2	122.08(17)
C4–C3–H3	119.0
C2–C3–H3	119.0
C3–C4–C5	121.67(18)
C3–C4–H4	119.2
C5–C4–H4	119.2
C6–C5–C4	115.17(18)
C6–C5–H5	122.4
C4–C5–H5	122.4
C5–C6–O1	127.37(16)
C5–C6–C1	124.34(16)
O1–C6–C1	108.28(15)
N1–C7–O1	115.50(14)
N1–C7–C8	127.10(15)
O1–C7–C8	117.40(14)
C9–C8–C13	119.48(14)
C9–C8–C7	120.28(14)
C13–C8–C7	120.23(14)
C10–C9–C8	120.48(14)
C10–C9–H9	119.8
C8–C9–H9	119.8
C9–C10–C11	120.07(14)
C9–C10–S1	119.26(12)
C11–C10–S1	120.62(12)
C12–C11–C10	119.93(14)
C12–C11–H11	120.0
C10–C11–H11	120.0
C11–C12–C13	120.49(15)
C11–C12–H12	119.8
C13–C12–H12	119.8
O2–C13–C12	117.83(15)
O2–C13–C8	122.64(15)
C12–C13–C8	119.53(15)
C19–C14–C15	120.22(17)
C19–C14–N2	108.51(14)
C15–C14–N2	131.27(18)
C16–C15–C14	116.61(18)
C16–C15–H15	121.7
C14–C15–H15	121.7
C15–C16–C17	121.97(17)
C15–C16–H16	119.0
C17–C16–H16	119.0
C18–C17–C16	122.29(18)
C18–C17–H17	118.9
C16–C17–H17	118.9
C19–C18–C17	114.38(18)
C19–C18–H18	122.8
C17–C18–H18	122.8
C14–C19–O6	108.14(15)
C14–C19–C18	124.53(16)
O6–C19–C18	127.33(16)
N2–C20–O6	115.48(14)
N2–C20–C21	125.97(15)
O6–C20–C21	118.54(14)
C22–C21–C26	119.74(15)
C22–C21–C20	120.56(14)
C26–C21–C20	119.70(15)
C23–C22–C21	120.10(14)
C23–C22–H22	120.0
C21–C22–H22	120.0

C22–C23–C24	120.30(15)
C22–C23–S2	119.68(12)
C24–C23–S2	119.98(12)
C25–C24–C23	120.11(15)
C25–C24–H24	119.9
C23–C24–H24	119.9
C24–C25–C26	120.44(15)
C24–C25–H25	119.8
C26–C25–H25	119.8
O7–C26–C25	117.54(15)
O7–C26–C21	123.15(15)
C25–C26–C21	119.30(15)
O1M–C1M–H1MA	109.5
O1M–C1M–H1MB	109.5
H1MA–C1M–H1MB	109.5
O1M–C1M–H1MC	109.5
H1MA–C1M–H1MC	109.5
H1MB–C1M–H1MC	109.5
C1M–O1M–Na1	118.09(11)
C1M–O1M–H1	110.0(17)
Na1–O1M–H1	116.9(17)
Na2–O1W–H1W1	112.8(16)
Na2–O1W–H2W1	116.3(16)
H1W1–O1W–H2W1	107(2)
Na2–O2W–Na2$^{\#6}$	92.91(5)
Na2–O2W–H1W2	104.7(15)
Na2$^{\#6}$–O2W–H1W2	122.6(16)
Na2–O2W–H2W2	123.6(15)
Na2$^{\#6}$–O2W–H2W2	111.3(15)
H1W2–O2W–H2W2	103.1(19)

Symmetry transformations used to generate equivalent atoms:
#1: 1-X, 1-Y, 2-Z; #2: 1-X, -0.5+Y, 1.5-Z; #3: 1-X, 0.5+Y, 1.5-Z; #4: +X, 0.5-Y, 0.5+Z; #5: +X, 0.5-Y, -0.5+Z; #6: 1-X, 1-Y, 1-Z;

Table S11. Torsion angles for **5**

**Atom–Atom–Atom–Atom**	**Torsion Angle [°]**
O5–S1–O3–Na2$^{\#1}$	8.51(11)
O4–S1–O3–Na2$^{\#1}$	−120.04(8)
C10–S1–O3–Na2$^{\#1}$	124.43(8)
Na1$^{\#2}$–S1–O3–Na2$^{\#1}$	−115.95(10)
O5–S1–O3–Na1$^{\#2}$	124.47(6)
O4–S1–O3–Na1$^{\#2}$	−4.09(8)
C10–S1–O3–Na1$^{\#2}$	−119.62(6)
O5–S1–O4–Na2$^{\#3}$	−14.42(10)
O3–S1–O4–Na2$^{\#3}$	114.33(8)
C10–S1–O4–Na2$^{\#3}$	−130.35(7)
Na1$^{\#2}$–S1–O4–Na2$^{\#3}$	110.75(8)
O5–S1–O4–Na1$^{\#2}$	−125.18(6)
O3–S1–O4–Na1$^{\#2}$	3.58(7)
C10–S1–O4–Na1$^{\#2}$	118.90(6)
O3–S1–O5–Na1	−66.37(14)
O4–S1–O5–Na1	60.82(15)
C10–S1–O5–Na1	177.33(12)
Na1$^{\#2}$–S1–O5–Na1	−9.92(17)
O10–S2–O8–Na1	59.46(11)
O9–S2–O8–Na1	−69.10(10)
C23–S2–O8–Na1	175.52(8)
Na1$^{\#4}$–S2–O8–Na1	−40.60(14)
O10–S2–O9–Na1$^{\#4}$	25.92(10)
O8–S2–O9–Na1$^{\#4}$	154.53(7)
C23–S2–O9–Na1$^{\#4}$	−90.89(8)
O8–S2–O10–Na2	−175.87(16)
O9–S2–O10–Na2	−47.8(2)
C23–S2–O10–Na2	68.91(19)
Na1$^{\#4}$–S2–O10–Na2	−31.25(17)
C7–N1–C1–C6	0.27(18)
C7–N1–C1–C2	179.65(18)
C6–C1–C2–C3	−0.5(3)
N1–C1–C2–C3	−179.86(17)
C1–C2–C3–C4	1.1(3)
C2–C3–C4–C5	−0.5(3)
C3–C4–C5–C6	−0.7(3)
C4–C5–C6–O1	179.91(16)
C4–C5–C6–C1	1.2(3)
C7–O1–C6–C5	−178.98(16)
C7–O1–C6–C1	−0.14(16)
C2–C1–C6–C5	−0.7(3)
N1–C1–C6–C5	178.81(15)
C2–C1–C6–O1	−179.54(15)
N1–C1–C6–O1	−0.07(18)
C1–N1–C7–O1	−0.38(18)
C1–N1–C7–C8	−179.61(15)
C6–O1–C7–N1	0.34(18)
C6–O1–C7–C8	179.64(13)
N1–C7–C8–C9	−177.49(15)
O1–C7–C8–C9	3.3(2)
N1–C7–C8–C13	4.0(3)
O1–C7–C8–C13	−175.22(14)
C13–C8–C9–C10	1.0(2)
C7–C8–C9–C10	−177.57(14)
C8–C9–C10–C11	0.5(2)
C8–C9–C10–S1	178.14(12)
O5–S1–C10–C9	15.53(14)
O3–S1–C10–C9	−105.55(13)

O4–S1–C10–C9	136.35(12)
Na1[#2]–S1–C10–C9	−157.94(10)
O5–S1–C10–C11	−166.88(13)
O3–S1–C10–C11	72.04(14)
O4–S1–C10–C11	−46.06(14)
Na1[#2]–S1–C10–C11	19.65(15)
C9–C10–C11–C12	−1.5(2)
S1–C10–C11–C12	−179.09(12)
C10–C11–C12–C13	1.0(2)
C11–C12–C13–O2	179.97(15)
C11–C12–C13–C8	0.5(2)
C9–C8–C13–O2	179.08(15)
C7–C8–C13–O2	−2.4(2)
C9–C8–C13–C12	−1.5(2)
C7–C8–C13–C12	177.07(15)
C20–N2–C14–C19	−0.30(18)
C20–N2–C14–C15	179.11(18)
C19–C14–C15–C16	−0.5(3)
N2–C14–C15–C16	−179.83(17)
C14–C15–C16–C17	−0.1(3)
C15–C16–C17–C18	0.3(3)
C16–C17–C18–C19	0.0(3)
C15–C14–C19–O6	−178.96(15)
N2–C14–C19–O6	0.53(18)
C15–C14–C19–C18	0.8(3)
N2–C14–C19–C18	−179.70(15)
C20–O6–C19–C14	−0.52(16)
C20–O6–C19–C18	179.71(16)
C17–C18–C19–C14	−0.5(3)
C17–C18–C19–O6	179.21(15)
C14–N2–C20–O6	−0.04(18)
C14–N2–C20–C21	−179.10(15)
C19–O6–C20–N2	0.36(18)
C19–O6–C20–C21	179.49(14)
N2–C20–C21–C22	176.85(15)
O6–C20–C21–C22	−2.2(2)
N2–C20–C21–C26	−3.5(2)
O6–C20–C21–C26	177.50(14)
C26–C21–C22–C23	1.4(2)
C20–C21–C22–C23	−178.95(14)
C21–C22–C23–C24	−1.3(2)
C21–C22–C23–S2	176.40(12)
O10–S2–C23–C22	9.34(15)
O8–S2–C23–C22	−110.91(13)
O9–S2–C23–C22	130.16(13)
Na1[#4]–S2–C23–C22	89.39(12)
O10–S2–C23–C24	−172.99(12)
O8–S2–C23–C24	66.76(14)
O9–S2–C23–C24	−52.17(14)
Na1[#4]–S2–C23–C24	−92.94(13)
C22–C23–C24–C25	0.4(2)
S2–C23–C24–C25	−177.30(12)
C23–C24–C25–C26	0.4(2)
C24–C25–C26–O7	−179.85(15)
C24–C25–C26–C21	−0.3(2)
C22–C21–C26–O7	178.92(14)
C20–C21–C26–O7	−0.8(2)
C22–C21–C26–C25	−0.6(2)
C20–C21–C26–C25	179.72(15)

Symmetry transformations used to generate equivalent atoms:
#1: +X, 0.5-Y, 0.5+Z; #2: 1-X, 1-Y, 2-Z; #3: 1-X, 0.5+Y, 1.5-Z; #4: 1-X, -0.5+Y, 1.5-Z;

14. References:

[1] a) Y. Li, X. Zhang, B. Zhu, J. Yan and W. Xu, *Analytical Sciences*, 2010, **26**, 1077; (b) Q. Mei, Y. Shi, Q. Hua and Bihai Tong ***RSC Adv.***, 2015,**5**, 74924-74931

[2] Bruker, *SAINT*, *V8.40B*, Bruker AXS Inc., Madison, Wisconsin, USA.

[3] L. Krause, R. Herbst-Irmer, G. M. Sheldrick and D. Stalke, *J. Appl. Cryst.*, 2015, **48**, 3–10.

[4] G. M. Sheldrick, *Acta Cryst.*, 2015, **A71**, 3–8, doi:10.1107/S2053273314026370.

[5] G. M. Sheldrick, *Acta Cryst.*, 2015, **C71**, 3–8, doi:10.1107/S2053229614024218.

[6] D. Kratzert, J. J. Holstein and I. Krossing, *J. Appl. Cryst.*, 2015, **48**, 933–938.

[7] D. Kratzert and I. Krossing, *J. Appl. Cryst.*, 2018, **51**, 928–934.

[8] C. R. Groom, I. J. Bruno, M. P. Lightfoot and S. C. Ward, *Acta Cryst.*, 2016, **B72**, 171–179.

[9] D. Kratzert, *FinalCif*, *V137*, https://dkratzert.de/finalcif.html.

^{1}H-NMR of **1**

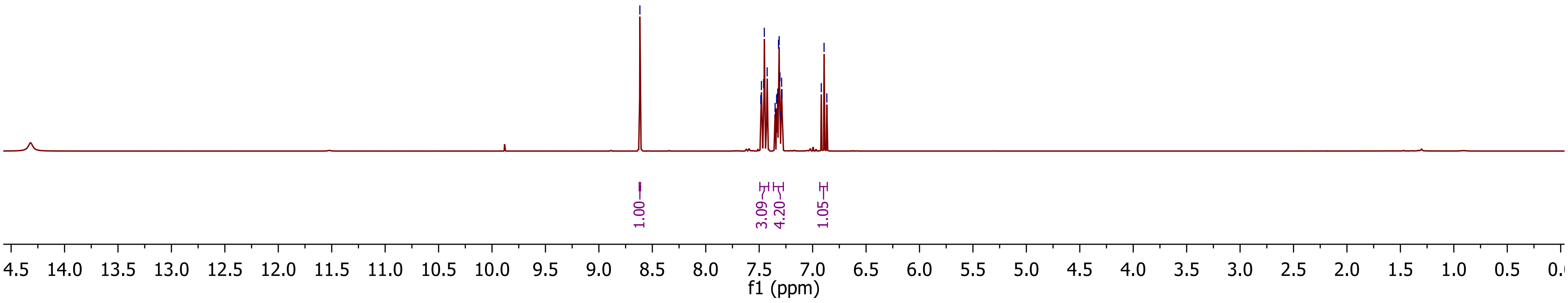
8.616
7.485
7.479
7.458
7.452
7.425
7.351
7.347
7.337
7.332
7.326
7.319
7.313
7.306
7.297
7.290
7.287
6.919
6.893
6.867
1.00
3.09
4.20
1.05
4.5 14.0 13.5 13.0 12.5 12.0 11.5 11.0 10.5 10.0 9.5 9.0 8.5 8.0 7.5 7.0 6.5 6.0 5.5 5.0 4.5 4.0 3.5 3.0 2.5 2.0 1.5 1.0 0.5
f1 (ppm)

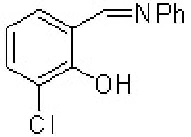
NPh
OH
Cl

1

^{13}C-NMR of **1**

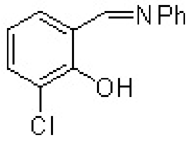
NPh
OH
Cl

1

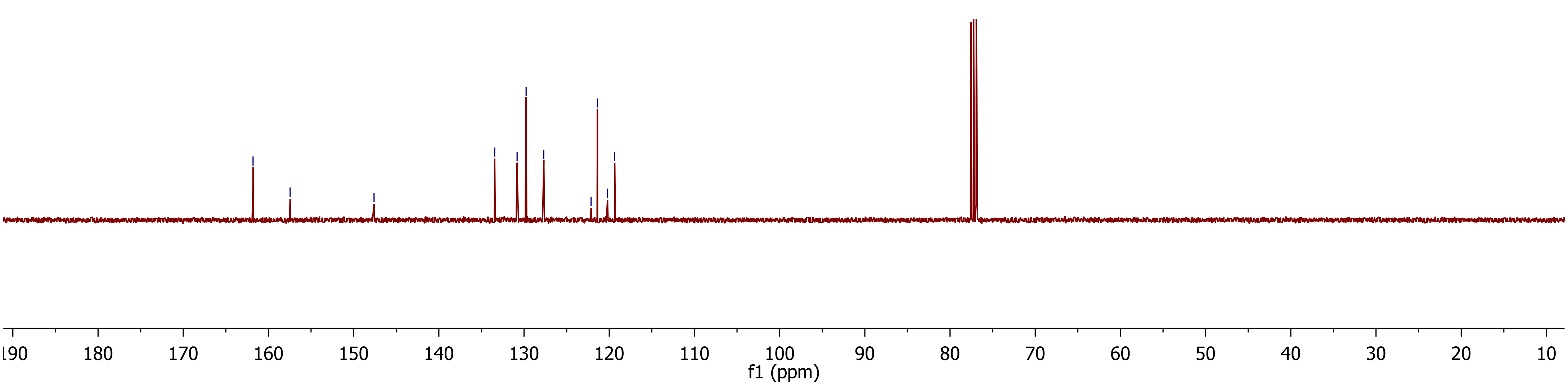
161.81
157.45
147.61
133.45
130.81
129.76
127.68
122.13
121.39
120.20
119.36
190
180
170
160
150
140
130
120
110
100
90
80
70
60
50
40
30
20
10
f1 (ppm)

^{1}H-NMR of **3**

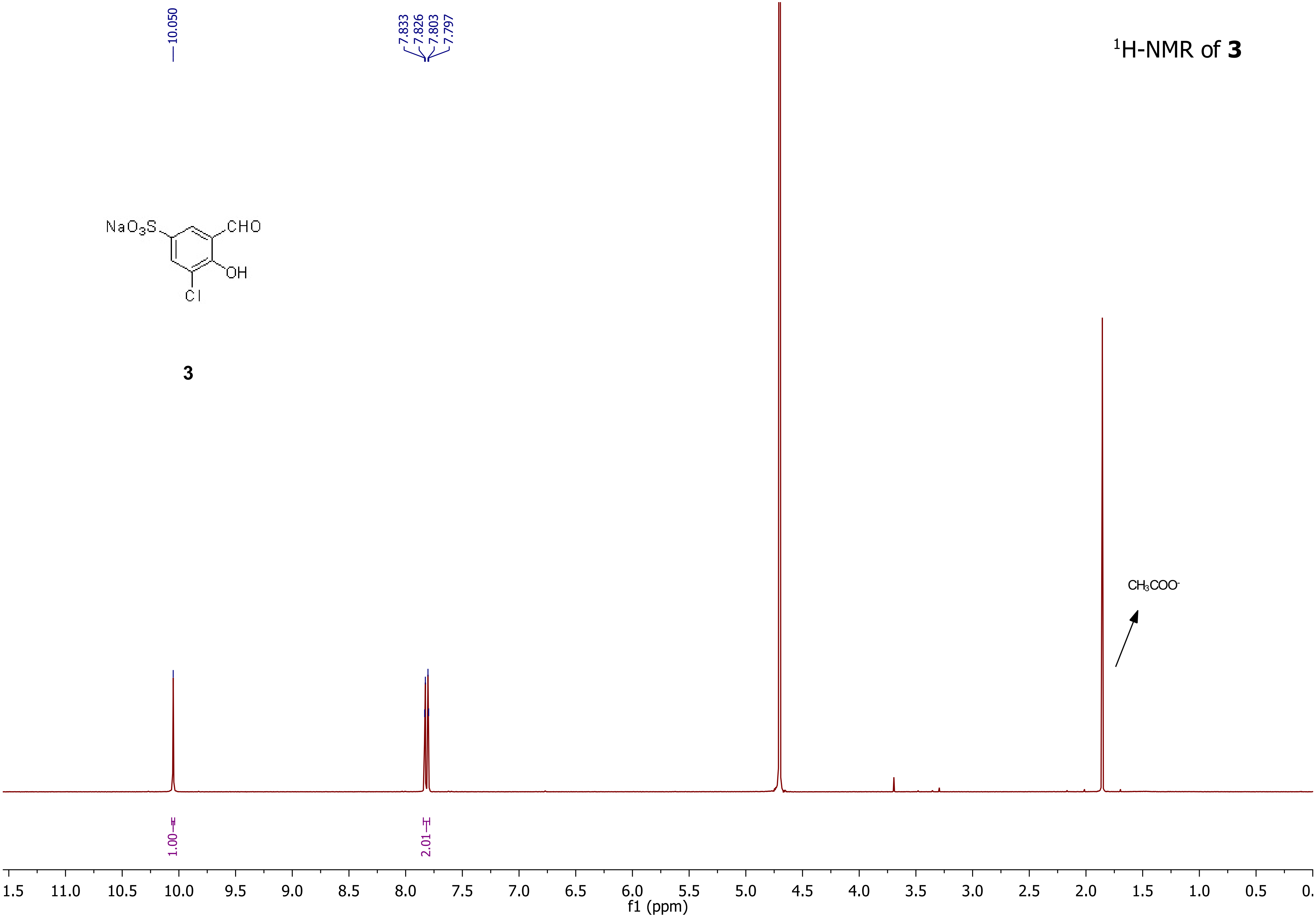

NaO3S
CHO
OH
Cl
3
10.050
7.833
7.826
7.803
7.797
CH3COO-
1.00
2.01
1.5 11.0 10.5 10.0 9.5 9.0 8.5 8.0 7.5 7.0 6.5 6.0 5.5 5.0 4.5 4.0 3.5 3.0 2.5 2.0 1.5 1.0 0.5 0.
f1 (ppm)

^{13}C-NMR of **3**

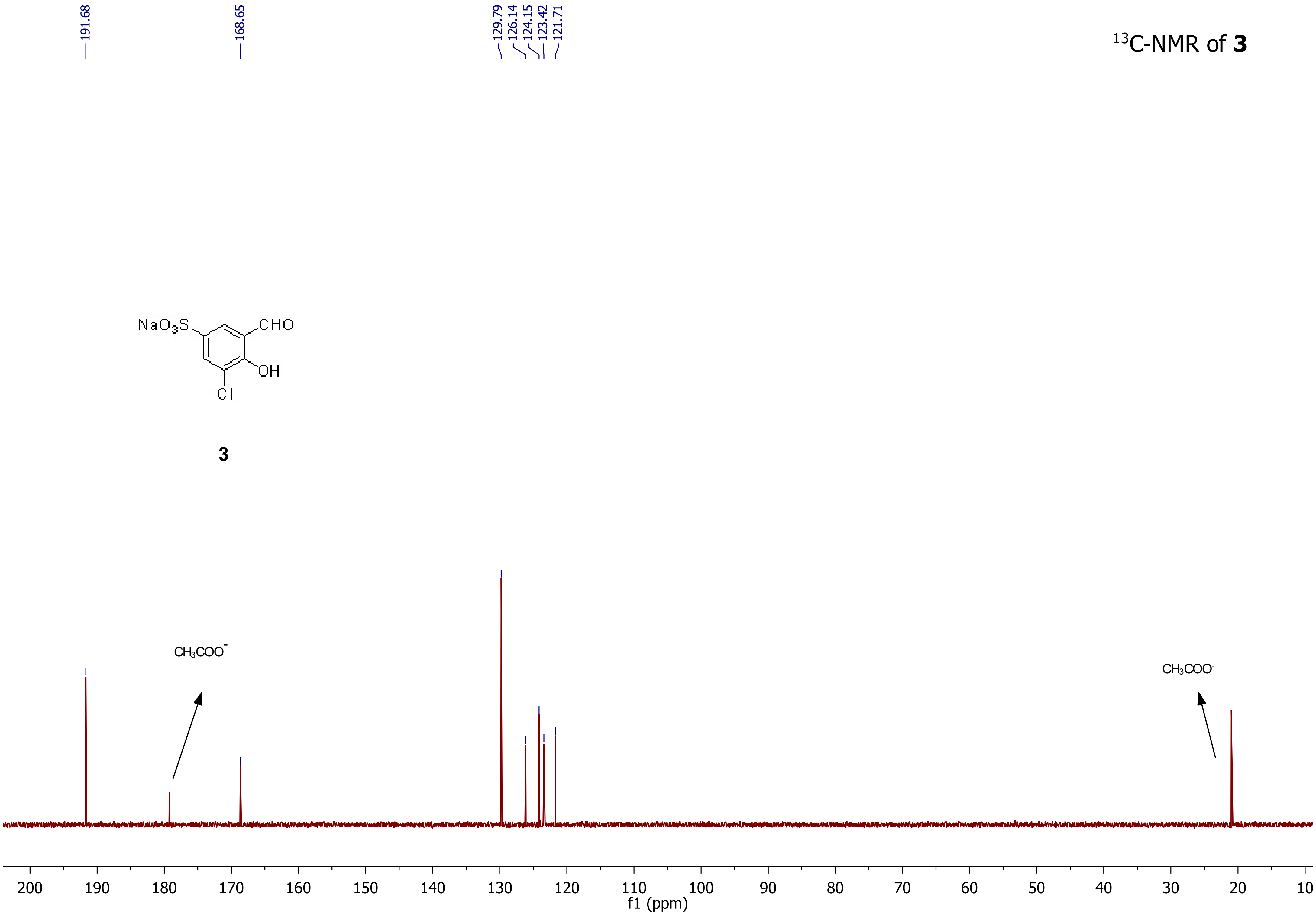
191.68
168.65
129.79
126.14
124.15
123.42
121.71
NaO3S
CHO
OH
Cl
3
CH3COO-
CH3COO-
200 190 180 170 160 150 140 130 120 110 100 90 80 70 60 50 40 30 20 10
f1 (ppm)

^{1}H-NMR of **4**

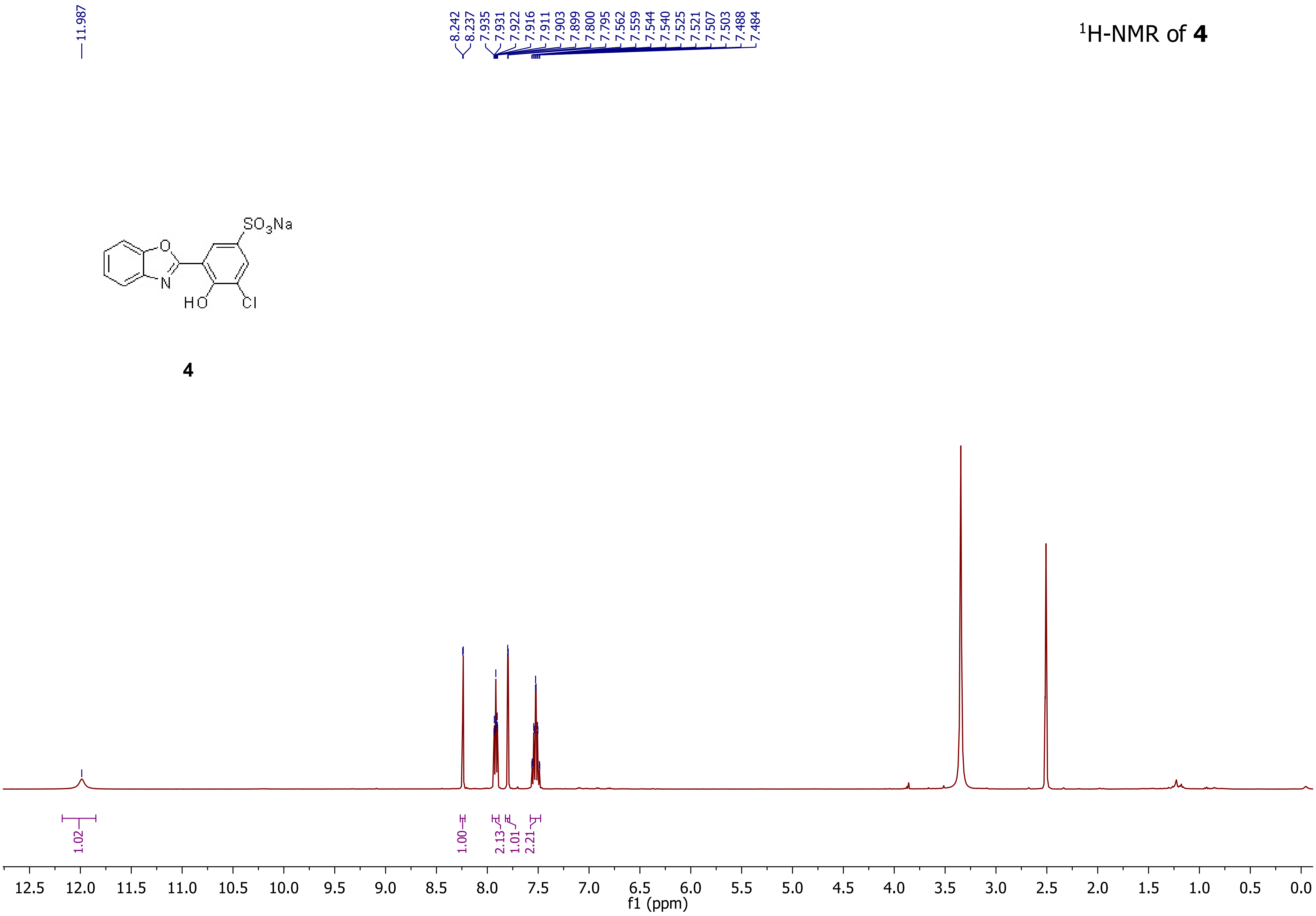
SO3Na
O
N
HO
Cl
4
11.987
8.242
8.237
7.935
7.931
7.922
7.916
7.911
7.903
7.899
7.800
7.795
7.562
7.559
7.544
7.540
7.525
7.521
7.507
7.503
7.488
7.484
1.02
1.00
2.13
1.01
2.21
12.5 12.0 11.5 11.0 10.5 10.0 9.5 9.0 8.5 8.0 7.5 7.0 6.5 6.0 5.5 5.0 4.5 4.0 3.5 3.0 2.5 2.0 1.5 1.0 0.5 0.0
f1 (ppm)

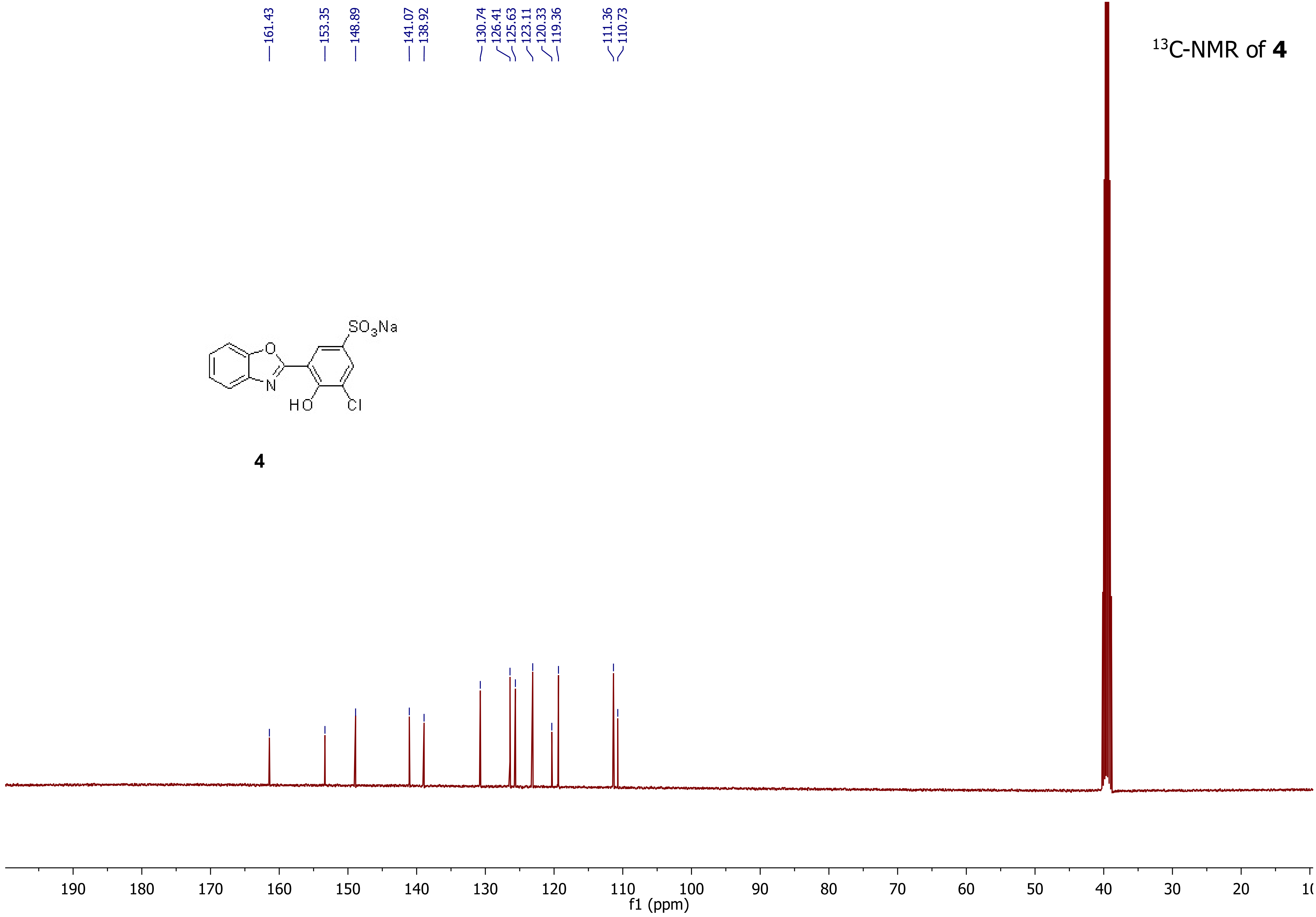

161.43
153.35
148.89
141.07
138.92
130.74
126.41
125.63
123.11
120.33
119.36
111.36
110.73
13C-NMR of 4
SO3Na
O
N
HO
Cl
4
190 180 170 160 150 140 130 120 110 100 90 80 70 60 50 40 30 20
f1 (ppm)

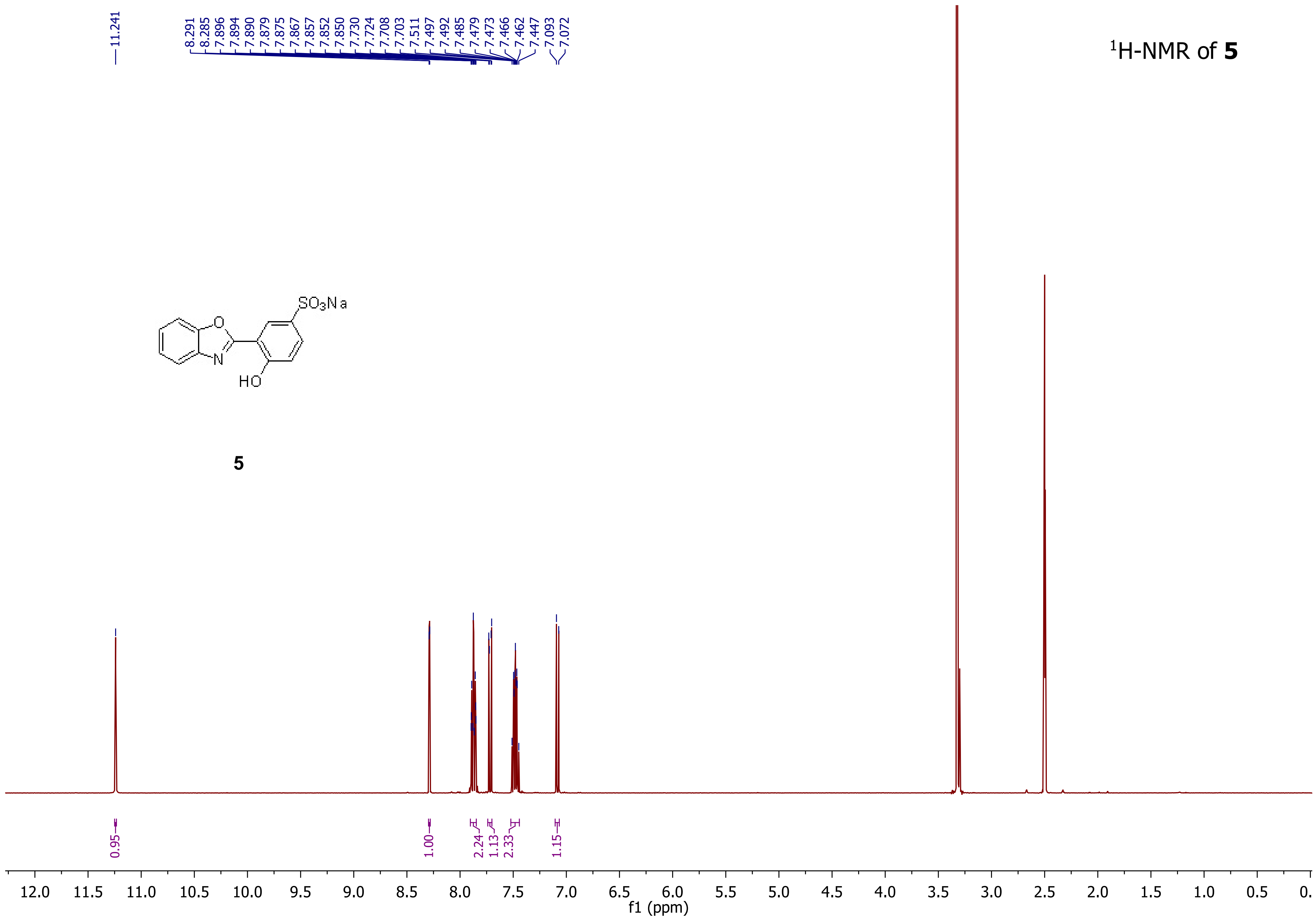

^{1}H-NMR of **5**
11.241
8.291
8.285
7.896
7.894
7.890
7.879
7.875
7.867
7.857
7.852
7.850
7.730
7.724
7.708
7.703
7.511
7.497
7.492
7.485
7.479
7.473
7.466
7.462
7.447
7.093
7.072
SO3Na
O
N
HO
5
0.95
1.00
2.24
1.13
2.33
1.15
12.0 11.5 11.0 10.5 10.0 9.5 9.0 8.5 8.0 7.5 7.0 6.5 6.0 5.5 5.0 4.5 4.0 3.5 3.0 2.5 2.0 1.5 1.0 0.5 0.
f1 (ppm)

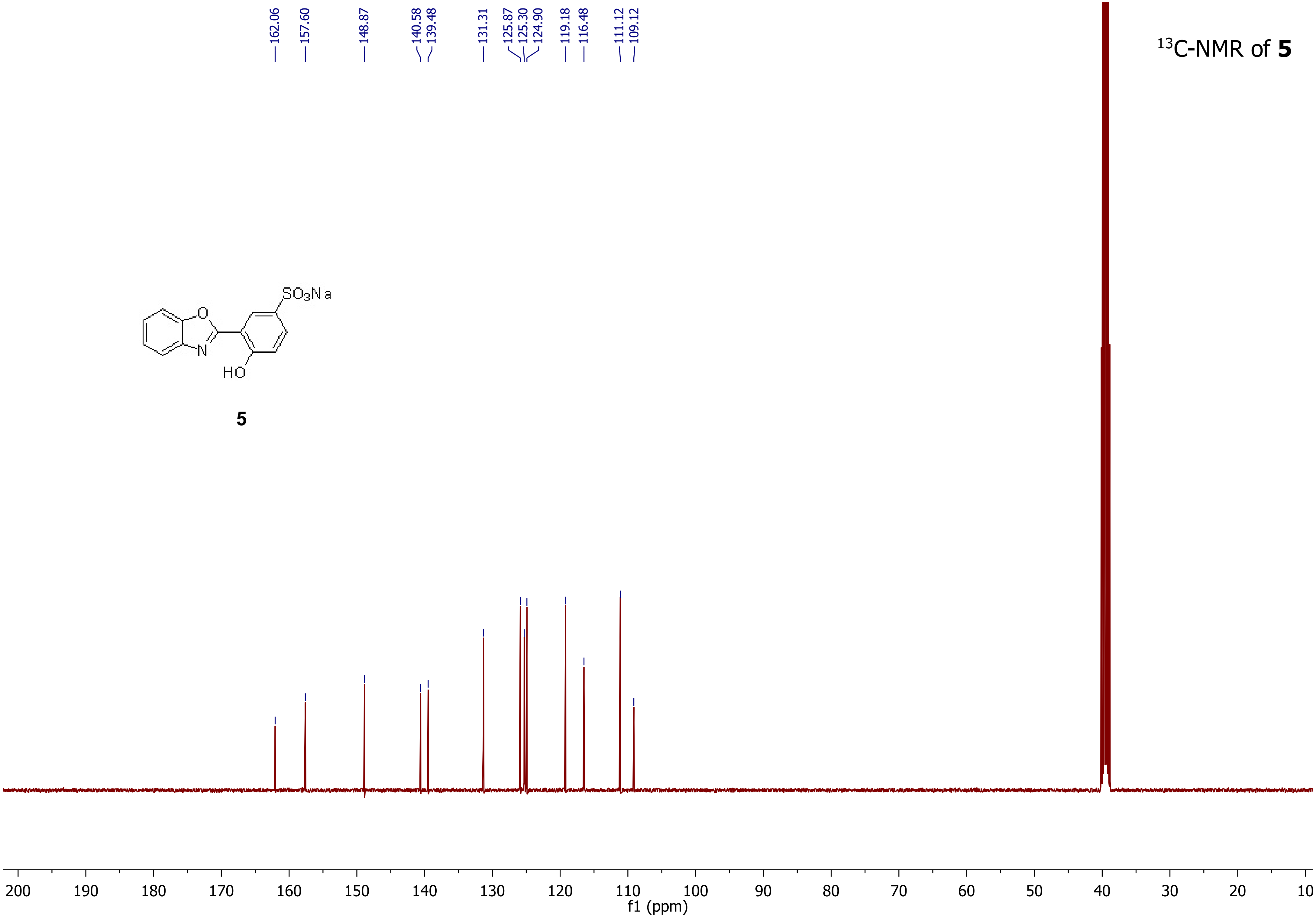
162.06
157.60
148.87
140.58
139.48
131.31
125.87
125.30
124.90
119.18
116.48
111.12
109.12
^{13}C-NMR of **5**
SO_3Na
O
N
HO
5
200 190 180 170 160 150 140 130 120 110 100 90 80 70 60 50 40 30 20 10
f1 (ppm)

^{31}P-NMR of **polyP4**

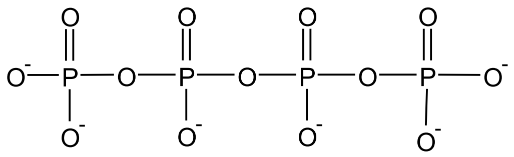

ammonium salt

polyP4

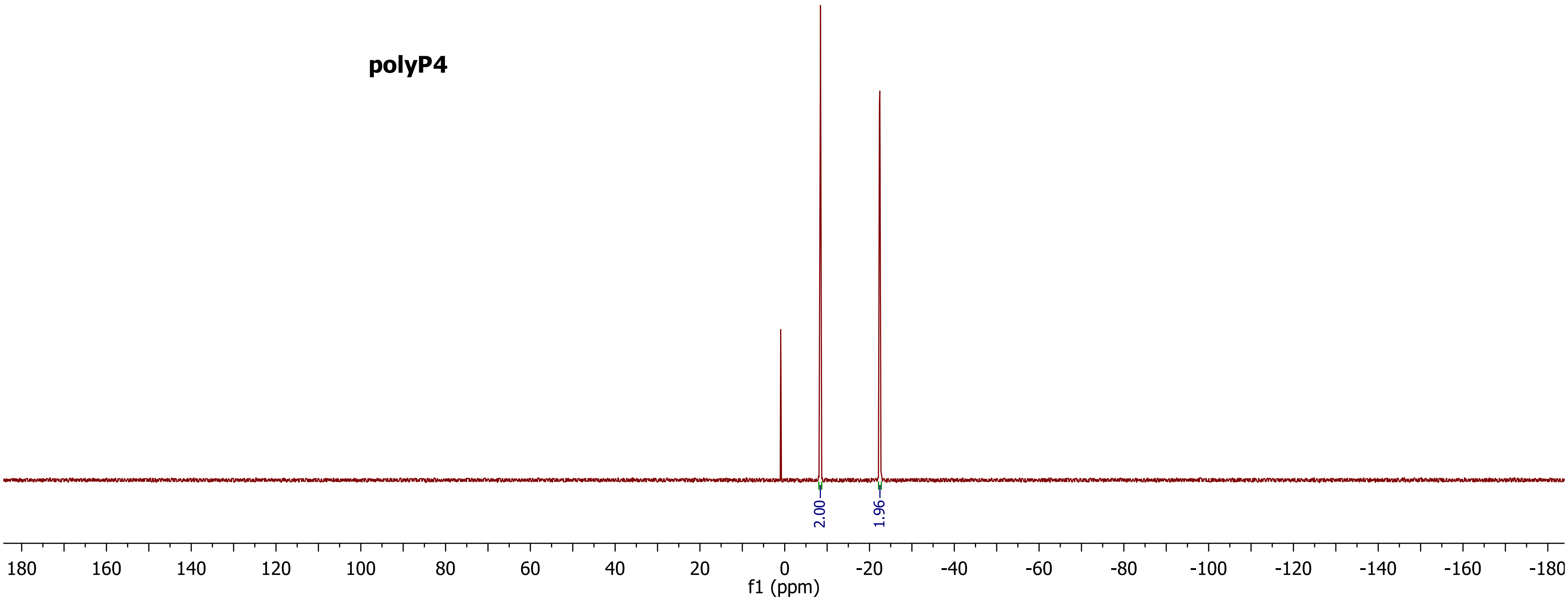

^{31}P-NMR of **polyP5**

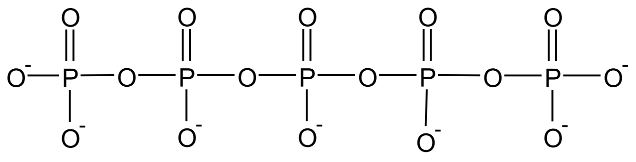

sodium salt

polyP5

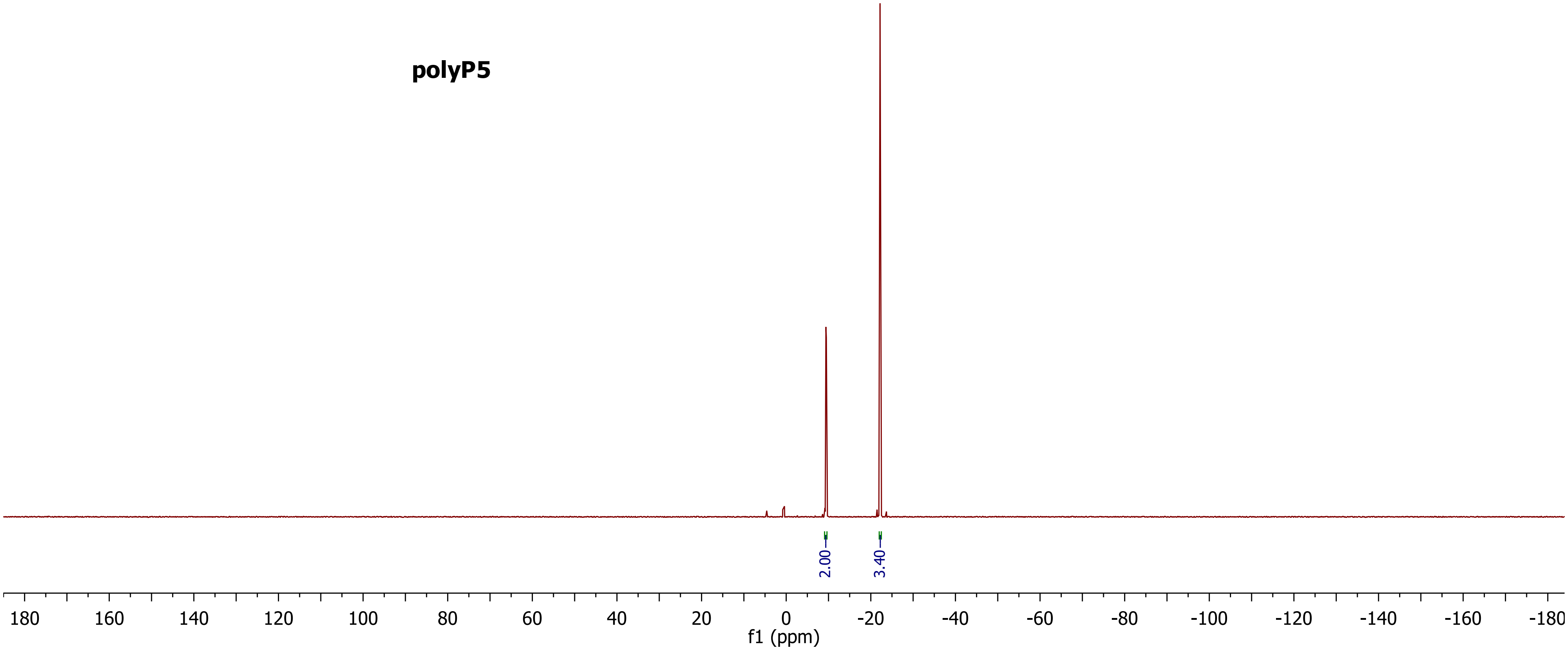

^{31}P-NMR of **polyP8**

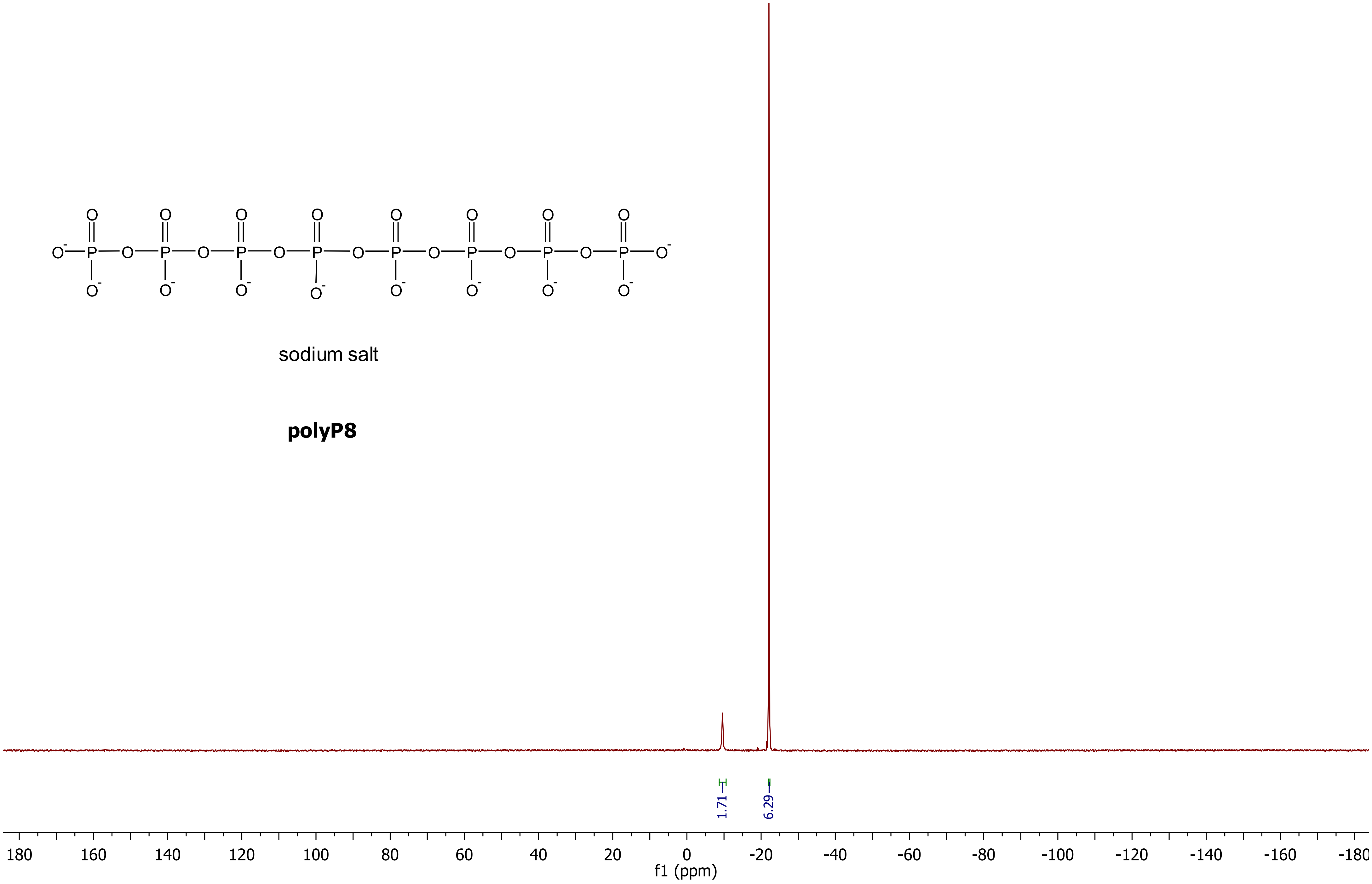

HRMS Spectra:

HRMS spectrum of **1**:

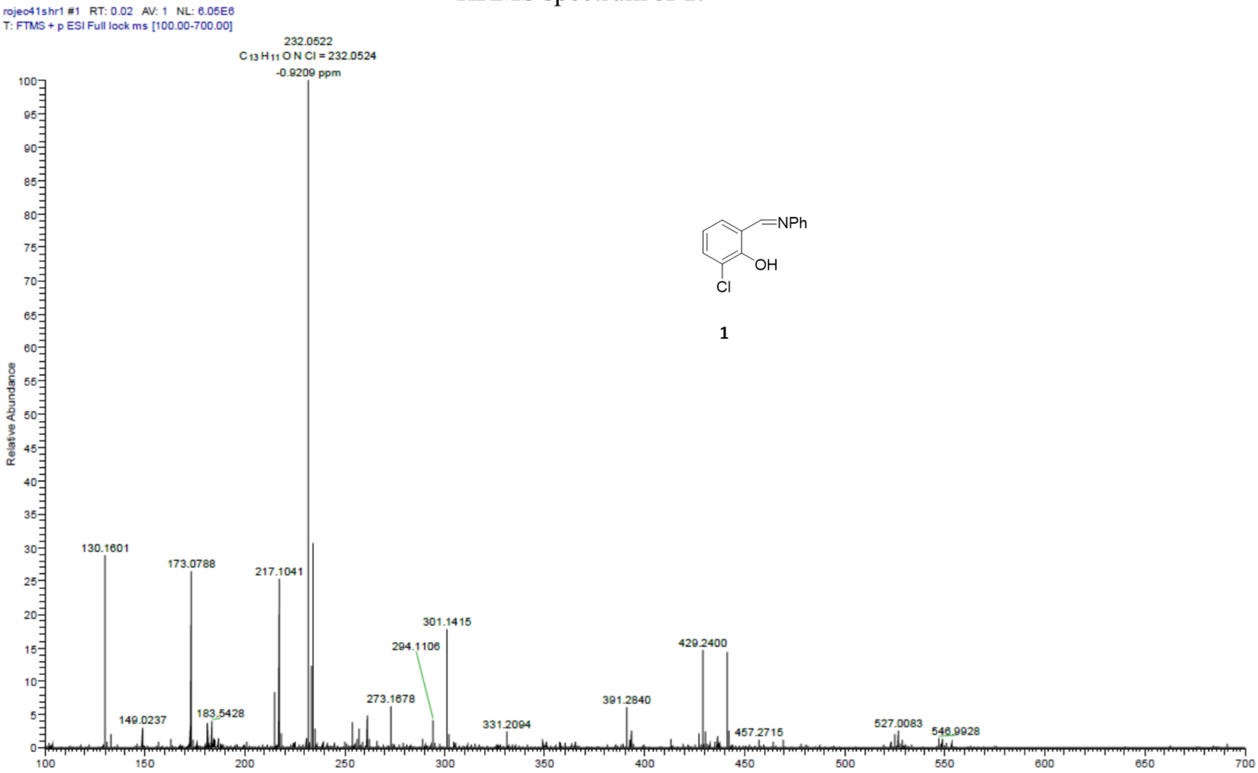

HRMS spectrum of **3**:

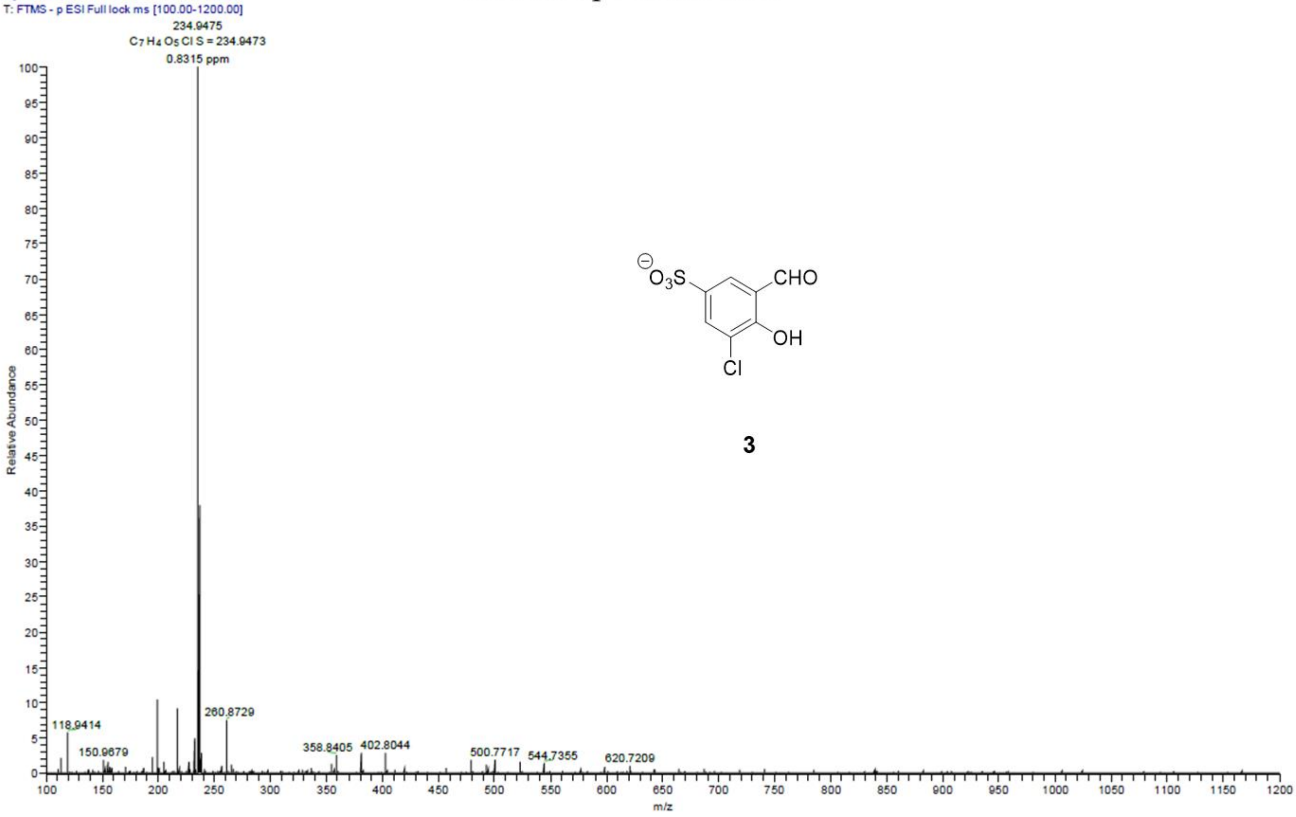

HRMS spectrum of **4**:

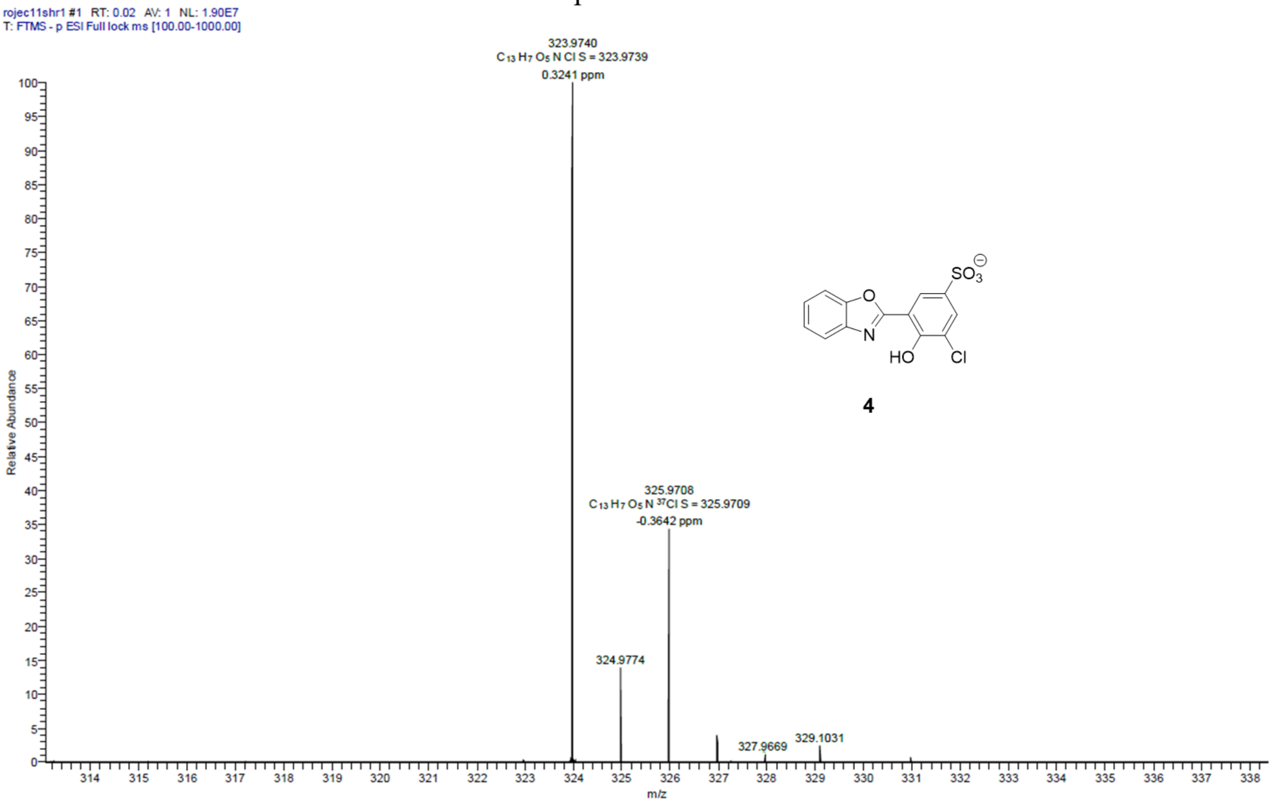

HRMS spectrum of **5**:

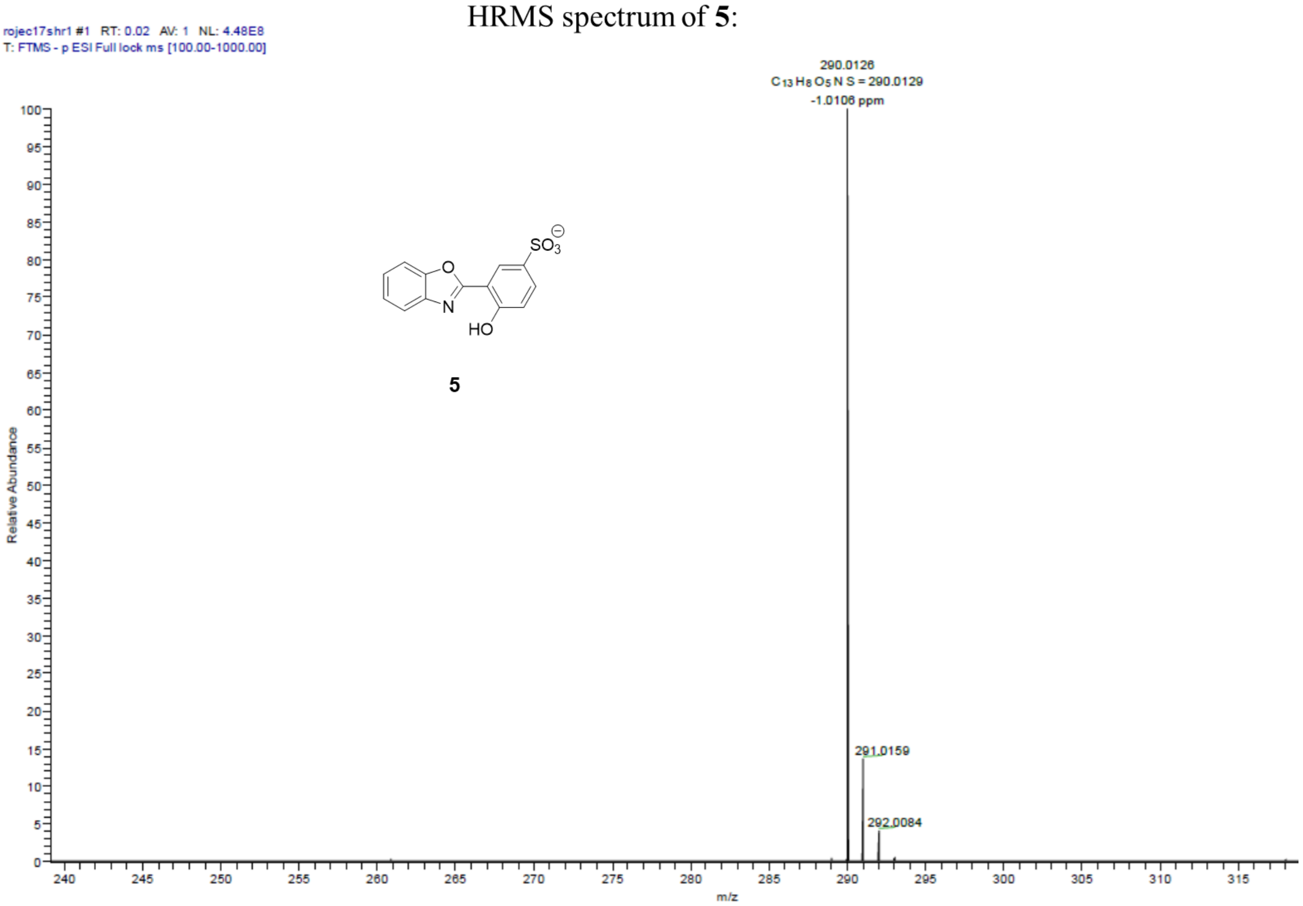

Supplement: OB-023-D4OB01926A-s001 [file OB-023-D4OB01926A-s001.pdf]
